# Economic Process Evaluation and Environmental Life-Cycle Assessment of Bio-Aromatics Production

**DOI:** 10.3389/fbioe.2020.00403

**Published:** 2020-05-13

**Authors:** Jens O. Krömer, Rafael G. Ferreira, Demetri Petrides, Norbert Kohlheb

**Affiliations:** ^1^Systems Biotechnology, Department of Solar Materials, Helmholtz Centre for Environmental Research – UFZ, Leipzig, Germany; ^2^Intelligen Brazil, São Paulo, Brazil; ^3^Intelligen Inc., Scotch Plains, NJ, United States; ^4^Department Environmental and Biotechnology Centre, Helmholtz Centre for Environmental Research – UFZ, Leipzig, Germany

**Keywords:** life-cycle assessment, process development, aromatics, fermentation, pHBA

## Abstract

The bio-based production of aromatics is experiencing a renaissance with systems and synthetic biology approaches promising to deliver bio-catalysts that will reach yields, rates, and titers comparable to already existing bulk bio-processes for the production of amino acids for instance. However, aromatic building blocks derived from petrochemical routes have a huge economic advantage, they are cheap, and very cheap in fact. In this article, we are trying to shed light on an important aspect of biocatalyst development that is frequently overlooked when working on strain development: economic and environmental impact of the production process. We estimate the production cost and environmental impact of a microbial fermentation process depending on culture pH, carbon source and process scale. As a model molecule we use *para*-hydroxybenzoic acid (pHBA), but the results are readily transferrable to other shikimate derived aromatics with similar carbon yields and production rates.

## Introduction

Aromatic chemicals are very important building blocks for the fiber, coating, resin, and packaging industries as well as precursors for the pharmaceutical and cosmetic industries (Krömer et al., 2013; Averesch and Krömer, 2018). Currently aromatics are almost exclusively derived from fossil fuels, despite the fact that biology potentially offers greener and more sustainable alternatives (Averesch and Krömer, 2018).

Despite initial work on the metabolic engineering of microbes for the production of aromatics starting in the mid 1990s (Barker and Frost, 2001), bio-production of aromatics has not yet been commercialized to the best of our knowledge. However, in recent years the interest in strain development for aromatics production has surged for a large variety of molecules (Averesch and Krömer, 2018) and one can only assume that this is also driven by revived commercial interest. The scope of molecules currently in focus can be clearly distinguished into high-value low-volume and low-value high-volume applications. A common assumption is that competitive production of lower volume higher price chemicals is easier to accomplish biotechnologically. This ignores the fact that a higher price also benefits the chemical synthesis for such molecules and in the end biotechnology always has to compete on production costs, no matter how expensive the molecule is. In addition, the unit production costs (UPC) will also sharply increase as the production volume decreases. So margins could be limited. A high price may not only be a reflection of the production cost but also a question of supply and demand. In general, only for molecules that cannot be chemically synthesized due to their complexity (monoclonal antibodies, proteins, and antibiotics, etc.) do biotechnological processes find a niche, without chemical competition, and here usually very high prices can be achieved. But for most aromatics chemical competition has to be faced with the low end of the price range probably covered by purified terephthalic acid (PTA) that has an estimated market volume of over 60 million metric tons globally and a price of around 0.65 $ per kg (09/2019).

Another common assumption is that a biotechnological process is greener and more sustainable (Mojsov, 2015), because the carbon sources are usually considered renewable (Harding, 2008). This assumption has to be carefully evaluated, since the production of renewable carbon sources for biotech processes has an ecological footprint as well. In addition, the biotechnological production of chemicals usually happens in very dilute aqueous solutions requiring energy, and/or resource intensive downstream processing, which quite often produces considerable waste streams (Stottmeister et al., 2005).

When looking at the biotechnological production of aromatics, the key metabolic pathway is the shikimate pathway (Averesch and Krömer, 2018), which is responsible in bacteria, fungi and plants for providing the precursors for the synthesis of aromatic amino acids, folate, and quinones. But, as demonstrated in the articles of this special issue, this pathway is a central step toward many interesting compounds such as cinnamic acid, dehydroshikimate, melanin, vanillin, and para-hydroxy benzoic acid (pHBA). The latter can be considered a mid-range molecule, which currently has an estimated world market of 50,000 t p.a. at a price of around 2,600 US$/t. pHBA is an essential component in liquid crystal polymers which find widespread use in electronics. pHBA is also a precursor for parabens, a class of preservatives in the pharma (Ma et al., 2016) and cosmetics industries (Matwiejczuk et al., 2020). For our early-stage process and sustainability assessment, we use pHBA as a model molecule. This molecule has been produced in lab scale in microbial systems from sugars, achieving titers (*T*) of 37 g/L, productivities (*R*) of over 1.5 g/l/h, and carbon yields (*Y*) of 66% (Kitade et al., 2018). While this is still too low for commercialization, the necessary TRY parameters could be achieved with further strain and process optimization.

In principle, higher value aromatics can also be derived from chemical synthesis and unless a cost benefit compared to the existing technology can be realized with a biological route, the bio-process cannot be implemented. The problem at this point is two-fold: On the one hand, researchers usually do not know the true production costs of existing processes and in the case of specialty molecules also do not easily get access to real market prices. On the other hand, researchers also lack information about the anticipated production costs of a newly developed bio-based building block and the impact on market price, if additional (bio-based) product volume is added to the market.

To the best of our knowledge, the bio-production of pHBA has not been realized commercially at large scale, and hence the presented model rests on a range of assumptions. Nevertheless, we hope to provide a starting point for researchers and bio-process developers placing their research agenda for developing bio-based aromatics on a firmer footing. At the same time, we strive to define some cost boundaries for the production of one of the simplest bio-based aromatics molecule and also provide an environmental impact assessment. We compare this to a reference scenario, which is the Kolbe–Schmitt carboxylation of potassium phenolate to pHBA. The model should be transferrable to other aromatic building blocks derived from the microbial shikimate pathway when adjusting to the respective theoretical carbon yields and assuming similar downstream processing.

## Process Modeling

We established a fermentation process in SuperPro Designer v10 (Intelligen Inc., Scotch Plains, NJ, United States). Due to the absence of a current commercial operation for pHBA bio-production, we developed the model under the core assumption that comparable production rates, yields, and downstream processing options that have been described for amino acids and organic acids could be achieved in the future (explained in detail below). The environmental impact assessment was done using the material and energy streams from SuperPro Designer as inputs into the life-cycle assessment (LCA) model. The SuperPro Designer models can be found online under “Resources”^1^. All inputs and detailed results are provided in the Supplementary Material.

Our LCA model is based on the ISO 14040:2006 standard (Finnveden et al., 2009; Guinee et al., 2011; Gargalo et al., 2016), and applies a cradle-to-gate LCA approach to capture the input material and energy flows from the cultivation and down-stream process. The production of 1 kg pHBA was determined to be the functional unit, and all necessary inputs were referenced to it. Using the CML2001–January 2016 characterization model, all the environmental impacts in this model were considered. The calculation was made using the LCA software, GaBi8© with GaBi Professional, GaBi Construction Materials, GaBi Food and Feed, and Ecoinvent databases.

We gained the economic indicator of UPC from the SuperPro Designer model which was calculated for all scenarios.

As the final step, the environmental and economic results were aggregated. Since the indicators fall into different categories, their aggregation is only possible when they are converted to the same dimension. In our case, we used a ratio scale approach; the simple internal normalization method where the highest number of categories was used as the normalization factor (Tugnoli et al., 2008; Patel et al., 2012). Then, the normalized value of the three sustainability dimensions is totaled and the alternatives ranked according to their overall value. The alternative with the lowest overall value is the most sustainable option relative to all other options.

In order to perform the simulations, a range of assumptions had to be made:

### Substrates

The carbon source assessed in this example is sucrose, which is a readily available carbon source that can be obtained at various purities, which helps with down-stream processing. For our environmental analysis we used two possible sources of sugar: from sugar beet and from sugar cane. This way we could capture the differences of the two production lines. Adapting the model to other carbon sources could be easily done. For the economic evaluation we assumed the average May 2019 spot price for white sugar of about 0.12 US$ per lb or 0.264 US$ per kg.^2^ For all other required media components and process chemicals we assume the prices given in the Supplementary Material. We sourced the prices from US-import statistics.^3^ These prices indicate the dutiable value on the US border prior to applying tariffs and in our opinion reflect the bulk prices for chemicals robustly. We assume that a mineral medium is based on a previously published yeast-based process (Averesch et al., 2017). For the bacterial process, it is assumed that sucrose metabolism is as active as glucose metabolism, which was previously demonstrated for certain strains of *Escherichia coli* (Arifin et al., 2014), for instance.

### Biocatalysts

For this simulation we assume a production either with a recombinant strain of baker’s yeast as described previously (Averesch et al., 2017) or an optimized strain of *Corynebacterium glutamicum* (Kitade et al., 2018). The yeast-based process could be run in an acidic pH range and potentially under anaerobic conditions, while the bacterial process needs to be controlled around pH 7.0 with sufficient aeration. The reported yields, rates, and titers are still sub-optimal (especially for the yeast scenario), therefore we assumed that we can achieve 90% of the theoretical maximum carbon yield as predicted with elementary flux mode analysis previously (Krömer et al., 2013). Another essential assumption is a minimum productivity of 4 gg_CDW_^–1^ h^–1^. The productivity will determine the fermentation time, which directly determines the number of batches per year and hence the required fermentation vessels for a given annual production. It will also determine running costs (energy etc.) per kg product produced. This assumed productivity is realistic, as it has been demonstrated for example for lysine production in *C. glutamicum* (Becker et al., 2011). Since the shikimate pathway also provides the essential amino acids Phenylalanine, Tyrosine, and Tryptophan and their combined anabolic demand exceeds the demand for Lysine (Stephanopoulos et al., 1998), it is a reasonable assumption that at least a comparable flux capacity exists in both the lysine and the shikimate pathways.

### Fermentation Section

The Fermentation section encompasses the mixing and sterilization of media components, seed fermentation steps to generate a sizable inoculum, and the main fermentation process that actually produces pHBA. Solutions of sucrose (50% w/w), sodium di-hydrogen phosphate (34.3 g/L), di-ammonium sulfate (3.14 g/L), and ammonium chloride (129.9 g/L) are prepared, heat-sterilized and stored in separate tanks. Each tank feeds the corresponding nutrient to the seed fermenters and to the main fermenter. The seed train is composed of three seed fermenters of increasing size: 40 L, 1 m^3^, and 10 m^3^; the latter is connected to the main fermenter, of 196 m^3^. In the first seed reactor biomass was expanded to 50 g/L, while we assumed that the subsequent larger reactors would reach 30 g/L of biomass. The whole broth was transferred as inoculum to the next stage. The seed fermenters operate in batch mode; the microbial growth in each one of them is represented by the following stoichiometric equation:

0.05(NH)4O2+41.99NHCl4+0.54NaHPO24

$$\;+17.42\mathrm{Sucrose}\to 7.84\mathrm{Biomass}+12.16\mathrm{CO}{}_{2}$$

Note that these coefficients are mass-based.

The main fermenter operates in fed-batch mode, in order to obtain a high level of pHBA. The cells first go through a growth phase, reaching a biomass concentration of 30 g/L, followed by a production phase which is triggered by the feed of carbon source under nitrogen limitation. The biosynthesis of pHBA is modeled by the following equation:

250.86(NH)4SO2+49951.81NHCl4+2695.02NaHPO24

  +204172.62Sucrose→22912.16Biomass

  +114158.15CO+280000pHBA(aq)

Again, these coefficients are mass-based. It is worth noting that the solubility of pHBA in pure water is rather low, equal to 5.79 g/L at 25°C (Nordstrom and Rasmuson, 2006). However, buffering pH around 7 will allow for higher concentrations of the deprotonated (anionic) form of pHBA in the solution; in fact, this is likely the reason why titers of over 36 g/L pHBA in *C. glutamicum* cultivation have been reported (Kitade et al., 2018). For this reason, in the bacterial case, calcium hydroxide is continuously added to the broth, in order to neutralize pHBA and therefore stabilize the pH. In the yeast case, however, no base is added; instead, an *in situ* product crystallization system was implemented. This strategy is described in further detail in the next section. Moreover, in both cases we assumed that a final titer of 100 g/L of pHBA, or the equivalent of pHBA calcium salt, could be achieved.

### Downstream Section

**FIGURE 1:**
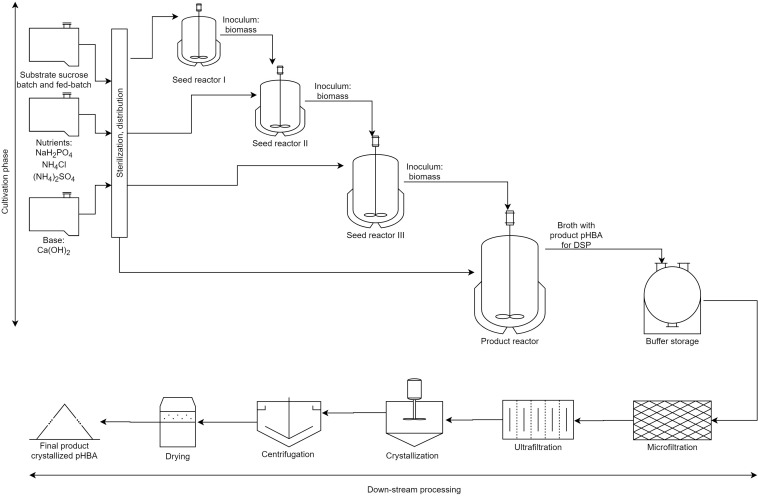
Simplified bacterial bioprocess where pH regulation is included (note that aeration is not detailed). The first part of the process contains the cultivation phase, where the biomass is produced in consecutive three seed reactors and then the fermentation is done. The second part presents the DSP with filtrations, crystallization, and centrifugation and drying. In the DSP of the yeast-based alternative no microfiltration is made.

In the bacterial scenario, we assumed that cells are first separated by microfiltration, and then the broth is concentrated threefold by ultrafiltration. Next, the pHBA salt is converted into the acid (uncharged) form by neutralization with nitric acid (70%), and the resulting product, which is highly insoluble in water, is isolated by crystallization at 5°C in an appropriate vessel. After that, the pHBA crystals are separated from the mother liquor with the aid of a basket centrifuge. Finally, the crystals are dried in a fluid-bed dryer with air in order to obtain a pHBA product with a purity of 99.5%. The complete bacterial process is schematically presented in Figure 1.

In the yeast scenario, on the other hand, we assume an *in situ* product removal system as described by Buque-Taboada et al. (2006). In this system, the broth is continuously removed from the fermenter; firstly, it passes through an ultrafilter that separates the cells and large contaminants from the pHBA solution, and those are recycled to the fermenter (approximately 15% in volume). Then, the clarified solution is cooled to 5°C in a crystallizer, and the resulting pHBA crystals are separated from the mother liquor using a basket centrifuge, similarly to the bacterial case. However, in the yeast case the liquid phase from the basket centrifuge is returned to the fermenter, while the pHBA crystals are dried in a fluid-bed dryer to 99.5% purity.

### Scenarios

In total we have considered 17 scenarios: As a reference chemical scenario (i) we use the LCA model for potassium phenolate production followed by the Kolbe–Schmitt carboxylation (5 atm CO_2_, 220; Markovic et al., 2006; Elvers, 2014). We assume a total conversion yield of 50% for the phenolate (Iijima and Yamaguchi, 2007; Elvers, 2014). The ratio of ortho-hydroxy benzoic acid to pHBA is at best 1:2.3 meaning that around a third of the product is salicylic acid (Markovic et al., 2006; Iijima and Yamaguchi, 2007), but for simplicity reasons we here assume that pHBA is exclusively made. Due to the lack of knowledge about the chemical downstream processing, we assume the same energy, and environmental footprint as we have for the biotech system.

The base scenarios are the aforementioned bacterial process (ii) and the yeast process (viii), based on sucrose derived from sugar beet. For the bacterial case we then developed scenarios in which it would be possible to recycle 90% of the water (iv) or to recycle 75% of the biomass as active catalyst (iii). In an optimized scenario both recycling strategies are combined and the sucrose is sourced from cane sugar (v). With this best case scenario of the bacterial process, the impact of scale-up was investigated. In the case of scale-up the plant produced 50,000 t p.a. instead of 10,000 t p.a. Both production with cane sugar (vi) and beet sugar (vii) was analyzed for the bacterial case. In the case of cane sugar based scenarios, labor costs were also adjusted to the average in Brazil (about 20% of the salaries assumed in SuperPro for US-based production). Finally, the respective scaled-up processes with cane and beet sugar were also analyzed for the yeast case (ix and x, respectively). While the recycling of biomass as active catalyst might be questionable in the real world, this provided an easy way to explore the economic impact of such a process. In reality maybe cell extracts could be recycled back into the seed reactors, but here many additional assumptions would have to be taken, especially around the problems in DSP arising from feeding bacterial or yeast extracts in the seed reactors.

Based on published titers, rates, and yields (TRY) in *C. glutamicum* (Kitade et al., 2018) one needs to achieve approximate improvements of 2.7, 43.6, and 1.3 fold, respectively, showing that production rate is still the most limiting factor amongst TRY parameters. We therefore performed a sensitivity analysis by also developing additional scenarios with longer fermentation durations. Fermentation time is determined by the time we need to achieve the target concentration and hence extended time equals a decreased production rate. All time dependent costs in the process will scale accordingly. We assumed a fermentation time of 54 and 72 h, equaling production rates of 2.67 gg_CDW_^–1^ h^–1^ and 2 gg_CDW_^–1^ h^–1^, respectively. To analyze the impact of fermentation duration and with this the impacts of productivity we developed 7 scenarios in addition. For the bacterial process this was a base case with 54 h (xi) and two best cases with cane sugar lasting for 54 and 72 h (xii and xiii, respectively). For the yeast process two base cases with 54 and 72 h duration (xiv and xv) and two best cases with cane sugar for 54 and 72 h (xvi and xvii) were developed. Inventory tables for scenarios are given in the Supplementary Material.

The environmental impact of electricity generation in our analysis assumes the current electricity mix in the US for the sugar beet cases and the electricity mix of Brazil for the cane sugar cases.

## Results

Generating a process model and then performing a LCA allowed us to study the largest drivers in price and environmental impact of a sugar based bio-production for aromatics.

### Economic Impact

**FIGURE 2:**
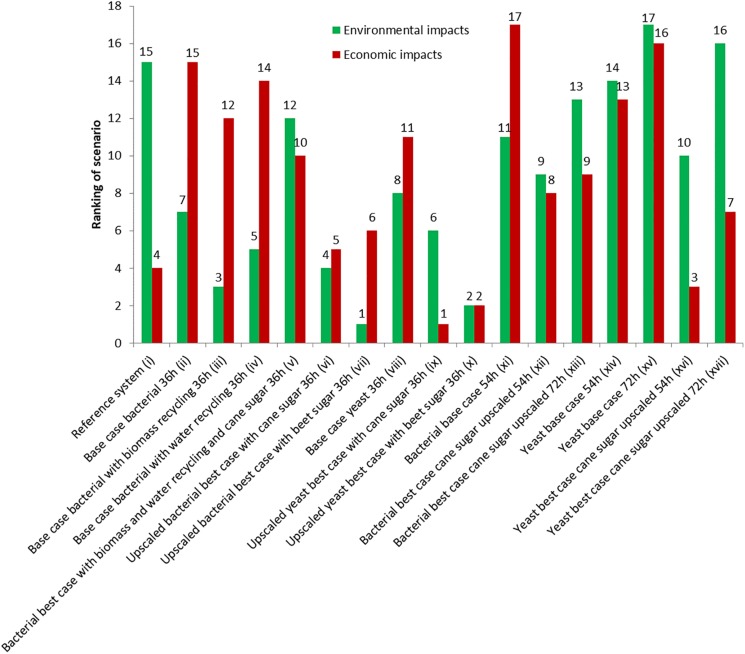
Overall ranking of the different scenarios based on their economic and environmental impacts.

The production cost for pHBA was assessed for our 17 scenarios (for details please refer to the Supplementary Information). This includes operating costs (incl. depreciation over 10 years) and investment costs (payback-time of around 6 years). For the reference case of petrochemical production (scenario i), we assume 2 $/kg. The implemented bio-process models predicted prices of between 1.61 and 3.59 $/kg, which is well above current world prices and most likely due to weak assumptions around the used equipment, rather than the raw material streams, and which are defined through the stoichiometry. The bio-production in the bacterial (ii) and the yeast base (viii) scenarios differed significantly, with 3.25 $/kg, and 2.65 $/kg. The reasons for this difference are the lower material costs, for the yeast case, since pH regulation is not required and the downstream processing is simpler. However, the yeast case employed downstream processing steps, which involved more labor dampening the impact on product cost. In both cases we assume a production scale of 10,000 t p.a., which is a significant share (20%) of the current world market. One of the largest by-products is biomass and the most important process stream besides sugar is water. In scenarios iii and iv we therefore explored the impact of the theoretical possibilities of recycling 75% of the biomass as active catalyst and of recycling 90% of the water. We found that water recycling only had a minor impact on cost (3.24 $/kg) while biomass recycling caused a drop to 3.07 $/kg. In a best-case scenario for the bacterial process (scenario v), we combined both recycling loops, and moved production to Brazil. Here the raw material would be sucrose from cane [same raw sugar price, but different environmental impact, and compared to sugar beet (scenario vii)] and labor costs would be significantly lower (∼20% of the default US labor costs assigned in SuperPro Designer). This lowered the production cost to 2.55 $/kg in the bacterial case and when upscaling this plant to 50,000 t p.a., which would be feasible under the parameters assumed for the bioprocess, the cost will drop to 2.01 $/kg. Making the same assumptions for the yeast process (scenario ix) will reduce the production cost even further to 1.61 $/kg. Ranking the first ten scenarios in terms of economic impact showed that based on our assumption a production based on yeast in large scale would be price competitive with the current chemical process (Figure 2).

Among the seven additional scenarios with different fermentation durations base cases for bacterial (xi–3.59 $/kg) and yeast (xv–3.4 $/kg) are obviously worse than their more effective counterparts (ii and viii). On the other hand it is important to underline that changes in fermentation duration have only minor effect on the economic performance, in this case, however, more main fermenters are needed which will drive investment costs up. Much more important are scale and substrate which are able to outperform the adverse effects of longer durations. For example in scenarios xvi and xvii, where upscaling, and switching to cane sugar considerably dropped the costs to 1.94 and 2.14 $/kg, respectively.

### Environmental Impact

**FIGURE 3:**
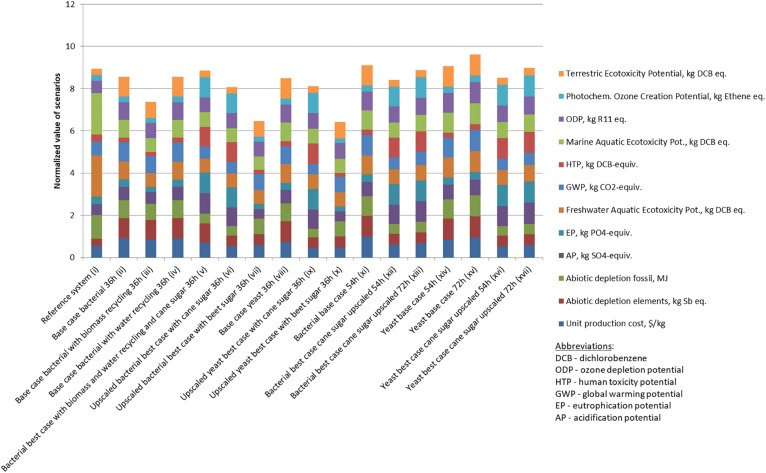
Contribution of different impact factors to the total impact of the different scenarios.

Overall, the scenarios were also ranked based on their total environmental impact (Figure 2). Biomass recycling had a much higher impact than the impact of water recycling (scenarios ii, iii, and iv). However, the highest impact was induced by switching the substrates (scenarios vi, vii, ix, and x): the overall trend for the bio-tech scenarios was that the cane-sugar based scenarios had a worse environmental impact than the processes based on sugar beet. In addition, upscaling induced some further environmental improvements. Consequently, a large scale production based on bacteria, and sugar from beets being the overall best process. When analyzing the patterns within the eleven different ecological criteria, a more complex pattern emerged (Figure 3). The processes based on sugar cane were in fact better than the scenarios based on sugar beet in the observed global warming potential (GWP) expressed as CO_2_ equivalents per kg product, ecotoxicology potential, and the fossil abiotic depletion potential, while the sugar-beet processes achieved similar or better values in the remaining criteria. Interestingly, the yeast and bacterial base cases were almost identical (e.g., scenarios ii and viii in Figure 3), highlighting that the alternative down-stream processing has minor impact on the ecology, but more on the economy.

The chemical production as our reference case (scenario i) here showed an overall comparable performance to the biotechnological processes, but it ranked only 15th in the overall ranking (Figure 2). Individual criteria are worse in the chemical case, while others are surprisingly good (e.g., abiotic depletion potential, acidification, and eutrophication potential, GWP) given in Figure 3. This is because the bioprocesses use chemicals such as nitric acid and sodium hydroxide that have a high GWP. In addition, the estimated electricity demand of the reference scenario is an order of magnitude less than of the bioprocesses which is the third most important factor producing greenhouse gases in the bioprocess.

**FIGURE 4:**
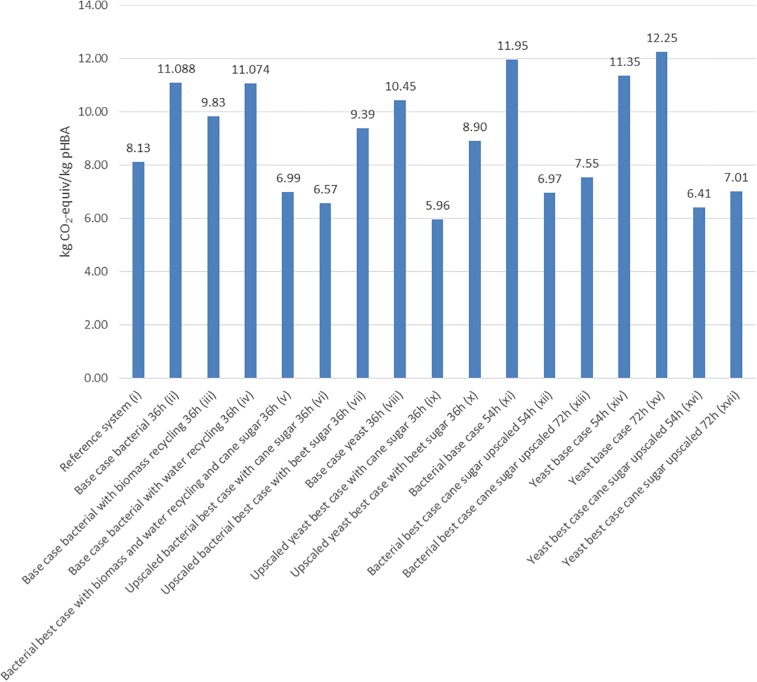
Global warming potential of the different scenarios given in kg CO_2_-equivalent per kg pHBA produced.

When looking at GWP only, the petrochemical route is actually amongst the better options (Figure 4). When comparing the ii–x biological scenarios amongst themselves, the two up-scaled processes (scenario vi and ix) lead to a reduction of GWP. In addition, shifting from sugar beets to sugar cane as substrate is highly beneficial (scenarios vi–vii and ix–x) as sugar cane has a much better yield per hectare and invested materials and hence has a smaller environmental footprint than sugar beet. In GWP the acidic yeast process slightly outcompetes the bacterial process. This is because increasing batch time raised GWP, e.g., for scenarios ii and viii, but again, differences are rather minor compared to changes in substrate.

An increase of fermentation time from 36 h to 54 h and 72 h obviously deteriorates environmental performance shown by scenarios xi–xvii in Figures 2, 3. Interestingly, up-scaled scenarios with fermentation time of 54 h (xii and xvi) are still better than the reference scenario (i), however, scenarios with longer fermentation time (xiii and xvii) are worse or equal (Figure 3).

## Discussion

The economic analysis clearly shows that the bio-based production of aromatics could be competitive with the current petrochemical route. As with most processes, the scale will have a dramatic impact on price and similar to the production of other weak acids, such as lactic acid for instance (Miller et al., 2017), a production at acidic pH is also a key target for pHBA production. It is obvious that producing 50,000 t p.a. of pHBA in a single fermentation facility would supply the entire current world demand in one location and this seems as such not a viable option from a market perspective. But the advantage of the bio-process would be that the same large-scale facility could sequentially produce different aromatic molecules for different markets, as long as the down-stream processing could be adapted. Upstream processes, such as inoculum preparation and substrate supply would be easily adjusted for the production of different aromatics with different strains of the same organism. That way, the beneficial economy of scale can be achieved, without flooding the market.

We assume here that the production cost in the petrochemical route is around the 2 $/kg mark. Obviously, this is an estimate, but considering the current market price of 2.6 $/kg, and the interest of a pHBA manufacturer (personal communication) in bio-based production around the benchmark of 1.5 $/kg suggests that this is a reasonable guess. This means that production of bio-based aromatics is within reach from a commercial perspective, if the titers, rates, and yields assumed above can be achieved.

From an environmental perspective it is clear that the chemical reference process does not seem to be generally performing worse than bio-based production. One has to weigh up the different criteria which will also depend on the location of a production facility and the available resources. It is obvious that the source for the sugar has a major impact. Here beet sugar or cane sugar have different advantages and disadvantages. Depending on the location, these impacts would weigh in differently. For instance, cane sugar will be better from resource depletion and global warming perspective, while beet-sugar is much better in terms of eutrophication, acidification, and toxicity potentials. A careful analysis for the respective location of the plant is needed. The choice of production host and hence DSP seems to be first of all an economic decision. Overall the production in yeast with beet sugar seems to perform best (scenario x – second both in economic and environmental terms). Finally, moving to biotechnology might become more of an economic than an environmental decision, especially when a reduction in oil production due to decarbonization of the energy markets might lead to increased barrel prices and hence an increase in chemical prices, long before we actually run out of oil.

## Data Availability Statement

All datasets generated for this study are included in the article/Supplementary Material.

## Author Contributions

NK and RF implemented the SuperPro Designer models. NK implemented the LCA models and conducted the assessment. DP provided expertise on bioprocess synthesis and cost analysis. JK designed the study and drafted the manuscript. All authors edited and approved the final manuscript.

## Conflict of Interest

RF and DP are employed by Intelligen Inc., the company that develops and markets SuperPro Designer, the process simulator used for the analysis of the various scenarios presented in this article. The remaining authors declare that the research was conducted in the absence of any commercial or financial relationships that could be construed as a potential conflict of interest. The handling Editor declared a past collaboration with one of the authors, JK.

## Footnotes

1 https://www.ufz.de/index.php?en=42204

2 https://www.isosugar.org

3 dataweb.usitc.gov
