# Supplementary material for: Economic Process Evaluation and Environmental Life-Cycle Assessment of Bio-Aromatics Production
Source: Front Bioeng Biotechnol. 2020 May 13;8:403. doi: 10.3389/fbioe.2020.00403 (PMC7237583; doi:10.3389/fbioe.2020.00403)
Supplement: Supplementary file 1 [file Data_Sheet_1.zip › Sc_1.pdf]

Materials & Streams Report

März 20, 2020

for Supplementary_1_reference_system_chemical_synthesis

1. OVERALL PROCESS DATA

Annual Operating Time	7.904,38 h
Unit Production Ref. Rate	10.000.000,00 kg MP/yr
Batch Size	25.839,79 kg MP
Recipe Batch Time	55,67 h
Recipe Cycle Time	20,33 h
Number of Batches per Year	387,00

MP = Total Flow of Stream 'Final Product'

2.1 STARTING MATERIAL REQUIREMENTS (per Section)

Section	Starting Material	Active Product	Amount Needed (kg Sin/kg MP)	Molar Yield (%)	Mass Yield (%)	Gross Mass Yield (%)
Main Section	(none)	(none)	0,00	Unknown	Unknown	Unknown

Sin = Section Starting Material, Aout = Section Active Product

2.2 BULK MATERIALS (Entire Process)

Material	kg/yr	kg/batch	kg/kg MP
Air	198.121.858	511.942,79	19,81
Carb. Dioxide	7.601.642	19.642,49	0,76
HCl (37% w/w)	8.026.215	20.739,57	0,80
KOH Solution	23.116.408	59.732,32	2,31
Phenol	15.713.844	40.604,25	1,57
Water	130.293.743	336.676,34	13,03
TOTAL	382.873.710	989.337,75	38,29

2.3 BULK MATERIALS (per Section)

SECTIONS IN: Main Branch

Main Section

Material	kg/yr	kg/batch	kg/kg MP
Air	198.121.858	511.942,79	19,81
Carb. Dioxide	7.601.642	19.642,49	0,76
HCl (37% w/w)	8.026.215	20.739,57	0,80
KOH Solution	23.116.408	59.732,32	2,31
Phenol	15.713.844	40.604,25	1,57
Water	130.293.743	336.676,34	13,03
TOTAL	382.873.710	989.337,75	38,29

2.4 BULK MATERIALS (per Material)

Air				
**Procedure**	**% Total**	**kg/yr**	**kg/batch**	**kg/kg MP**
Main Section (Main Branch)				
P-27	100,00	198.121.858	511.942,79	19,81
TOTAL	100,00	198.121.858	511.942,79	19,81

Carb. Dioxide				
**Procedure**	**% Total**	**kg/yr**	**kg/batch**	**kg/kg MP**
Main Section (Main Branch)				
P-1	100,00	7.601.642	19.642,49	0,76
TOTAL	100,00	7.601.642	19.642,49	0,76

HCl (37% w/w)				
**Procedure**	**% Total**	**kg/yr**	**kg/batch**	**kg/kg MP**
Main Section (Main Branch)				
P-3	100,00	8.026.215	20.739,57	0,80
TOTAL	100,00	8.026.215	20.739,57	0,80

KOH Solution				
**Procedure**	**% Total**	**kg/yr**	**kg/batch**	**kg/kg MP**
Main Section (Main Branch)				
P-1	100,00	23.116.408	59.732,32	2,31
TOTAL	100,00	23.116.408	59.732,32	2,31

Phenol				
**Procedure**	**% Total**	**kg/yr**	**kg/batch**	**kg/kg MP**
Main Section (Main Branch)				
P-1	100,00	15.713.844	40.604,25	1,57
TOTAL	100,00	15.713.844	40.604,25	1,57

Water				
**Procedure**	**% Total**	**kg/yr**	**kg/batch**	**kg/kg MP**
Main Section (Main Branch)				
P-1	84,21	109.719.519	283.512,97	10,97
P-11	15,79	20.574.225	53.163,37	2,06
TOTAL	100,00	130.293.743	336.676,34	13,03

2.5 BULK MATERIALS: SECTION TOTALS (kg/kg MP)

Raw Material	Main Section
Air	19,81
Carb. Dioxide	0,76
HCl (37% w/w)	0,80
KOH Solution	2,31
Phenol	1,57
Water	13,03
TOTAL	38,29

2.6 BULK MATERIALS: SECTION TOTALS (kg/batch)

Raw Material	Main Section
Air	511.942,79
Carb. Dioxide	19.642,49
HCl (37% w/w)	20.739,57
KOH Solution	59.732,32
Phenol	40.604,25
Water	336.676,34
TOTAL	989.337,75

2.7 BULK MATERIALS: SECTION TOTALS (kg/yr)

Raw Material	Main Section
Air	198.121.858
Carb. Dioxide	7.601.642
HCl (37% w/w)	8.026.215
KOH Solution	23.116.408
Phenol	15.713.844
Water	130.293.743
TOTAL	382.873.710

3. STREAM DETAILS

Stream Name	Phenol	KOH 40%	CO2	Water
**Source**	**INPUT**	**INPUT**	**INPUT**	**INPUT**
**Destination**	**P-1**	**P-1**	**P-1**	**P-1**
Stream Properties				
Activity (U/ml)	0,00	0,00	0,00	0,00
Temperature (°C)	25,00	60,00	25,00	25,00
Pressure (bar)	1,01	1,01	5,07	1,01
Density (g/L)	1.071,78	1.335,85	8,99	994,70
Total Enthalpy (kW-h)	588,44	3.091,64	1.353,86	8.273,51
Specific Enthalpy (kcal/kg)	12,47	44,53	59,30	25,11
Heat Capacity (kcal/kg-°C)	0,50	0,74	0,20	1,00
Component Flowrates (kg/batch)				
Carb. Dioxide	0,00	0,00	19.642,49	0,00
KOH	0,00	23.892,93	0,00	0,00
Phenol	40.604,25	0,00	0,00	0,00
Water	0,00	35.839,39	0,00	283.512,97
TOTAL (kg/batch)	40.604,25	59.732,32	19.642,49	283.512,97
TOTAL (L/batch)	37.884,83	44.714,97	2.183.857,07	285.022,35

Stream Name	Vent	S-104	S-107	S-109
Source	P-1	P-1	P-31	P-13
Destination	OUTPUT	P-31	P-3	P-11
Stream Properties				
Activity (U/ml)	0,00	0,00	0,00	0,00
Temperature (°C)	117,17	25,80	25,80	5,00
Pressure (bar)	0,03	1,01	1,01	1,01
Density (g/L)	0,02	1.081,46	1.081,46	702,57
Total Enthalpy (kW-h)	34.613,36	9.009,84	9.009,84	1.877,32
Specific Enthalpy (kcal/kg)	540,93	22,24	22,24	4,37
Heat Capacity (kcal/kg-°C)	0,41	0,86	0,86	0,86
Component Flowrates (kg/batch)				
Carb. Dioxide	10.215,69	0,00	0,00	0,00
HCl	0,00	0,00	0,00	295,14
KCl	0,00	0,00	0,00	15.085,04
KOH	0,00	238,93	238,93	238,93
Nitrogen	478,53	0,00	0,00	0,00
Oxygen	145,27	0,00	0,00	0,00
pHBA (aq)	0,00	0,00	0,00	1.715,84
pHBA (solid)	0,00	0,00	0,00	26.235,29
pHBA Salt	0,00	35.661,11	35.661,11	0,00
Phenol	799,94	126,47	126,47	126,47
PhO-K+	0,00	28.982,32	28.982,32	28.982,32
Water	43.418,04	283.529,40	283.529,40	296.595,33
TOTAL (kg/batch)	55.057,47	348.538,23	348.538,23	369.274,36
TOTAL (L/batch)	2.828.228.581,34	322.284,66	322.284,66	525.604,61

Stream Name	Water for Cake Wash	Wastewater	S-106	Air for Drying
**Source**	**INPUT**	**P-11**	**P-11**	**INPUT**
**Destination**	**P-11**	**OUTPUT**	**P-14**	**P-27**
Stream Properties				
Activity (U/ml)	0,00	0,00	0,00	0,00
Temperature (°C)	25,00	7,65	22,70	25,00
Pressure (bar)	1,01	1,01	2,12	1,01
Density (g/L)	994,70	1.050,24	1.350,53	1,18
Total Enthalpy (kW-h)	1.551,42	3.151,95	457,05	3.599,47
Specific Enthalpy (kcal/kg)	25,11	7,02	10,90	6,05
Heat Capacity (kcal/kg-°C)	1,00	0,92	0,48	0,24
Component Flowrates (kg/batch)				
KCl	0,00	15.085,04	0,00	0,00
KOH	0,00	238,93	0,00	0,00
Nitrogen	0,00	0,00	0,00	392.720,46
Oxygen	0,00	0,00	0,00	119.222,32
pHBA (aq)	0,00	1.715,84	0,00	0,00
pHBA (solid)	0,00	524,71	25.710,58	0,00
Phenol	0,00	126,47	0,00	0,00
PhO-K+	0,00	28.982,31	0,01	0,00
Water	53.163,37	339.390,65	10.368,05	0,00
TOTAL (kg/batch)	53.163,37	386.063,94	36.078,65	511.942,79
TOTAL (L/batch)	53.446,41	367.597,28	26.714,44	434.136.197,77

Stream Name	S-116	Humid Air	Final Product	HCl 37%
Source	P-27	P-14	P-14	INPUT
Destination	P-14	OUTPUT	OUTPUT	P-3
Stream Properties				
Activity (U/ml)	0,00	0,00	0,00	0,00
Temperature (°C)	37,66	50,00	50,00	25,00
Pressure (bar)	1,21	1,01	1,01	1,01
Density (g/L)	1,35	1,08	1.572,62	4,02
Total Enthalpy (kW-h)	5.422,66	14.738,22	408,77	1.097,31
Specific Enthalpy (kcal/kg)	9,11	24,28	13,61	45,52
Heat Capacity (kcal/kg-°C)	0,24	0,25	0,27	0,70
Component Flowrates (kg/batch)				
HCl	0,00	0,00	0,00	7.673,64
KCl	0,00	0,00	0,00	0,00
KOH	0,00	0,00	0,00	0,00
Nitrogen	392.720,46	392.720,46	0,00	0,00
Oxygen	119.222,32	119.222,32	0,00	0,00
pHBA (aq)	0,00	0,00	0,00	0,00
pHBA (solid)	0,00	0,00	25.710,58	0,00
Phenol	0,00	0,00	0,00	0,00
PhO-K+	0,00	0,00	0,01	0,00
Water	0,00	10.238,86	129,20	13.065,93
TOTAL (kg/batch)	511.942,79	522.181,64	25.839,79	20.739,57
TOTAL (L/batch)	377.960.346,66	485.609.472,46	16.431,04	5.162.125,66

Stream Name	S-108
Source	P-3
Destination	P-13
Stream Properties	
Activity (U/ml)	0,00
Temperature (°C)	25,67
Pressure (bar)	1,01
Density (g/L)	677,90
Total Enthalpy (kW-h)	9.491,85
Specific Enthalpy (kcal/kg)	22,12
Heat Capacity (kcal/kg-°C)	0,86
Component Flowrates (kg/batch)	
HCl	295,14
KCl	15.085,04
KOH	238,93
pHBA (aq)	27.951,13
Phenol	126,47
PhO-K+	28.982,32
Water	296.595,33
TOTAL (kg/batch)	369.274,36
TOTAL (L/batch)	544.735,44

4. OVERALL COMPONENT BALANCE (kg/batch)

COMPONENT	INITIAL	INPUT	OUTPUT	FINAL	IN-OUT
Carb. Dioxide	0,00	19.642,49	10.215,69	520,63	8.906,17
HCl	0,00	7.673,64	0,00	295,14	7.378,50
KCl	0,00	0,00	15.085,04	0,00	- 15.085,04
KOH	0,00	23.892,93	238,93	0,00	23.654,00
Nitrogen	532,28	392.720,46	393.198,99	269,62	- 215,87
Oxygen	161,59	119.222,32	119.367,60	81,85	- 65,53
pHBA (aq)	0,00	0,00	1.715,84	0,00	- 1.715,84
pHBA (solid)	0,00	0,00	26.235,29	0,00	- 26.235,29
Phenol	0,00	40.604,25	926,41	0,00	39.677,84
PhO-K+	0,00	0,00	28.982,32	0,00	- 28.982,32
Water	0,00	385.581,66	393.176,75	0,00	- 7.595,08
TOTAL	693,87	989.337,75	989.142,85	1.167,23	278,47
				Overall Error:	0,028%

5. EQUIPMENT CONTENTS

R-101				
Procedure	Operation	Time (in h)	Volume (in L)	Vapor (in kg)
P-1	START	0,00	0,00	422,27
P-1	CHARGE-PHENOL (Charge)	0,50	37.884,83	422,27
P-1	CHARGE-KOH (Charge)	1,00	87.734,86	422,27
P-1	AGITATE (Agitation)	15,33	87.734,85	422,27
P-1	HEAT-1 (Batch Heating)	2,27	53.954,66	169,85(*)
P-1	EVACUATE-1 (Evacuation)	2,85	53.954,66	5,14(*)
P-1	HEAT-2 (Batch Heating)	2,85	55.130,44	5,14(*)
P-1	SYNTHESIZE-PHENOXIDE-FORM (Batch Stoich. Reaction)	6,00	37.965,74	188,34(*)
P-1	COOL-1 (Batch Cooling)	6,25	38.151,71	211,80
P-1	CHARGE-CO2 (Charge)	7,25	38.140,62	19.854,28(*)
P-1	HEAT-3 (Batch Heating)	8,52	52.457,50	20.969,04(*)
P-1	SYNTHESIZE-pHBA-SALT (Batch Stoich. Reaction)	13,25	52.457,50	1.677,32(*)
P-1	COOL-2 (Batch Cooling)	14,83	37.180,93	530,90(*)
P-1	DISSOLVE-SALT-IN-WATER (Charge)	15,33	322.284,66	530,90(*)
P-1	TRANSFER-TO-FILTER (Transfer Out)	20,33	0,00	600,50(*)

(*) Contains material in vapor phase other than Oxygen & Nitrogen

BCFBD-101				
Procedure	Operation	Time (in h)	Volume (in L)	Vapor (in kg)
P-11	START	20,33	0,00	45,27
P-11	FILTER-1 (Cloth Filtration)	37,83	4.453,87	94,46(*)
P-11	CAKE-WASH-1 (Cake Wash)	38,08	4.452,41	94,46(*)
P-11	TRANSFER-OUT-1 (Transfer Out)	38,33	0,00	94,46(*)

(*) Contains material in vapor phase other than Oxygen & Nitrogen

DE-101				
Procedure	Operation	Time (in h)	Volume (in L)	Vapor (in kg)
P-31	START	15,33	0,00	0,00
P-31	FILTER-1 (Dead-End Filtration)	35,33	0,00	0,00
