# Supplementary material for: Economic Process Evaluation and Environmental Life-Cycle Assessment of Bio-Aromatics Production
Source: Front Bioeng Biotechnol. 2020 May 13;8:403. doi: 10.3389/fbioe.2020.00403 (PMC7237583; doi:10.3389/fbioe.2020.00403)
Supplement: Supplementary file 1 [file Data_Sheet_1.zip › Sc_10.pdf]

Materials & Streams Report

März 25, 2020

for Supplementary_10_yeast_best_case_beet_sugar_upscaled

1. OVERALL PROCESS DATA

Annual Operating Time	7,918.35 h
Unit Production Ref. Rate	50,000,000.00 kg MP/yr
Batch Size	76,452.60 kg MP
Recipe Batch Time	82.35 h
Recipe Cycle Time	12.00 h
Number of Batches per Year	654.00

MP = Total Flow of Stream 'Final Product'

2.1 STARTING MATERIAL REQUIREMENTS (per Section)

Section	Starting Material	Active Product	Amount Needed (kg Sin/kg MP)	Molar Yield (%)	Mass Yield (%)	Gross Mass Yield (%)
Fermentation Section	(none)	(none)	0.00	Unknown	Unknown	Unknown
Downstream Section	(none)	(none)	0.00	Unknown	Unknown	Unknown

Sin = Section Starting Material, Aout = Section Active Product

2.2 BULK MATERIALS (Entire Process)

Material	kg/yr	kg/batch	kg/kg MP
Air	1,563,868,771	2,391,236.65	31.28
Amm. Sulfate	186,097	284.55	0.00
Ammonium Chlori	7,382,592	11,288.37	0.15
H3PO4 (2%)	13,587,321	20,775.72	0.27
NaH2PO4	1,999,241	3,056.94	0.04
NaOH (0.5 M)	21,143,056	32,328.83	0.42
Sucrose	145,424,816	222,362.10	2.91
Water	624,185,640	954,412.29	12.48
TOTAL	2,377,777,534	3,635,745.47	47.56

2.3 BULK MATERIALS (per Section)

SECTIONS IN: Main Branch

Fermentation Section

Material	kg/yr	kg/batch	kg/kg MP
Air	521,745,521	797,776.03	10.43
Amm. Sulfate	186,097	284.55	0.00
Ammonium Chlori	7,382,592	11,288.37	0.15
H3PO4 (2%)	13,587,321	20,775.72	0.27
NaH2PO4	1,999,241	3,056.94	0.04
NaOH (0.5 M)	21,143,056	32,328.83	0.42
Sucrose	145,424,816	222,362.10	2.91
Water	508,769,811	777,935.49	10.18
TOTAL	1,220,238,455	1,865,808.03	24.40

Downstream Section

Material	kg/yr	kg/batch	kg/kg MP
Air	1,042,123,250	1,593,460.63	20.84
Water	115,415,829	176,476.80	2.31
TOTAL	1,157,539,080	1,769,937.43	23.15

2.4 BULK MATERIALS (per Material)

Air

Procedure	% Total	kg/yr	kg/batch	kg/kg MP
Fermentation Section (Main Branch)				
P-51	33.36	521,745,521	797,776.03	10.43
Downstream Section (Main Branch)				
P-3	66.64	1,042,123,250	1,593,460.63	20.84
TOTAL	100.00	1,563,868,771	2,391,236.65	31.28

Amm. Sulfate

Procedure	% Total	kg/yr	kg/batch	kg/kg MP
Fermentation Section (Main Branch)				
P-36	100.00	186,097	284.55	0.00
TOTAL	100.00	186,097	284.55	0.00

Ammonium Chlori

Procedure	% Total	kg/yr	kg/batch	kg/kg MP
Fermentation Section (Main Branch)				
P-38	100.00	7,382,592	11,288.37	0.15
TOTAL	100.00	7,382,592	11,288.37	0.15

H3PO4 (2%)

Procedure	% Total	kg/yr	kg/batch	kg/kg MP
Fermentation Section (Main Branch)				
P-4	54.42	7,394,535	11,306.63	0.15
P-1	10.11	1,374,183	2,101.20	0.03
P-15	32.02	4,350,039	6,651.44	0.09
P-16	3.45	468,564	716.46	0.01
TOTAL	100.00	13,587,321	20,775.72	0.27

NaH2PO4

Procedure	% Total	kg/yr	kg/batch	kg/kg MP
Fermentation Section (Main Branch)				
P-34	100.00	1,999,241	3,056.94	0.04
TOTAL	100.00	1,999,241	3,056.94	0.04

NaOH (0.5 M)

Procedure	% Total	kg/yr	kg/batch	kg/kg MP
Fermentation Section (Main Branch)				
P-4	82.31	17,403,803	26,611.32	0.35
P-1	6.56	1,386,121	2,119.45	0.03
P-15	8.89	1,880,498	2,875.38	0.04
P-16	2.24	472,634	722.68	0.01
TOTAL	100.00	21,143,056	32,328.83	0.42

Sucrose

Procedure	% Total	kg/yr	kg/batch	kg/kg MP
Fermentation Section (Main Branch)				
P-9	100.00	145,424,816	222,362.10	2.91
TOTAL	100.00	145,424,816	222,362.10	2.91

Water

Procedure	% Total	kg/yr	kg/batch	kg/kg MP
Fermentation Section (Main Branch)				
P-4	2.62	16,350,015	25,000.02	0.33
P-34	9.19	57,347,067	87,686.65	1.15
P-36	9.48	59,160,435	90,459.38	1.18
P-38	8.33	51,963,715	79,455.22	1.04
P-9	23.30	145,424,816	222,362.10	2.91
P-18	0.01	58,323	89.18	0.00
P-21	2.25	14,040,025	21,467.93	0.28
P-23	0.25	1,563,936	2,391.34	0.03
P-25	24.78	154,664,845	236,490.59	3.09
P-1	0.49	3,038,449	4,645.95	0.06

P-15	0.66	4,122,149	6,302.98	0.08
P-16	0.17	1,036,039	1,584.16	0.02
Downstream Section (Main Branch)				
P-11	18.49	115,415,829	176,476.80	2.31
TOTAL	100.00	624,185,640	954,412.29	12.48

2.5 BULK MATERIALS: SECTION TOTALS (kg/kg MP)

Raw Material	Fermentation Section	Downstream Section
Air	10.43	20.84
Amm. Sulfate	0.00	0.00
Ammonium Chlori	0.15	0.00
H3PO4 (2%)	0.27	0.00
NaH2PO4	0.04	0.00
NaOH (0.5 M)	0.42	0.00
Sucrose	2.91	0.00
Water	10.18	2.31
TOTAL	24.40	23.15

2.6 BULK MATERIALS: SECTION TOTALS (kg/batch)

Raw Material	Fermentation Section	Downstream Section
Air	797,776.03	1,593,460.63
Amm. Sulfate	284.55	0.00
Ammonium Chlori	11,288.37	0.00
H3PO4 (2%)	20,775.72	0.00
NaH2PO4	3,056.94	0.00
NaOH (0.5 M)	32,328.83	0.00
Sucrose	222,362.10	0.00
Water	777,935.49	176,476.80
TOTAL	1,865,808.03	1,769,937.43

2.7 BULK MATERIALS: SECTION TOTALS (kg/yr)

Raw Material	Fermentation Section	Downstream Section
Air	521,745,521	1,042,123,250
Amm. Sulfate	186,097	0
Ammonium Chlori	7,382,592	0
H3PO4 (2%)	13,587,321	0
NaH2PO4	1,999,241	0
NaOH (0.5 M)	21,143,056	0
Sucrose	145,424,816	0
Water	508,769,811	115,415,829
TOTAL	1,220,238,455	1,157,539,080

3. STREAM DETAILS

Stream Name	Air for Drying	S-104	Water for NH4Cl	NH4Cl
Source	INPUT	P-3	INPUT	INPUT
Destination	P-3	P-14	P-38	P-38
Stream Properties				
Activity (U/ml)	0.00	0.00	0.00	0.00
Temperature (°C)	25.00	37.66	10.00	20.00
Pressure (bar)	1.01	1.21	1.01	1.01
Density (g/L)	1.18	1.35	1,000.17	1,519.00
Total Enthalpy (kW-h)	11,222.63	16,886.13	930.36	98.60
Specific Enthalpy (kcal/kg)	6.06	9.12	10.07	7.52
Heat Capacity (kcal/kg-°C)	0.24	0.24	1.01	0.38
Component Flowrates (kg/batch)				
Ammonium Chlori	0.00	0.00	0.00	11,288.37
Argon	14,659.84	14,659.84	0.00	0.00
Carb. Dioxide	637.38	637.38	0.00	0.00
Nitrogen	1,244,333.40	1,244,333.40	0.00	0.00
Oxygen	333,830.00	333,830.00	0.00	0.00
Water	0.00	0.00	79,455.22	0.00
TOTAL (kg/batch)	1,593,460.63	1,593,460.63	79,455.22	11,288.37
TOTAL (L/batch)	1,351,307,636.87	1,176,452,701.93	79,441.57	7,431.45

Stream Name	Cl-Solution	S-129	NH4Cl to SFR-1	NH4Cl to SFR-2
Source	P-38	P-37	P-5	P-5
Destination	P-37	P-5	P-16	P-64
Stream Properties				
Activity (U/ml)	0.00	0.00	0.00	0.00
Temperature (°C)	10.50	35.00	35.00	35.00
Pressure (bar)	1.01	1.01	1.01	1.01
Density (g/L)	1,044.38	1,035.84	1,035.84	1,035.84
Total Enthalpy (kW-h)	1,028.95	3,413.98	0.65	16.18
Specific Enthalpy (kcal/kg)	9.76	32.37	32.37	32.37
Heat Capacity (kcal/kg-°C)	0.93	0.92	0.92	0.92
Component Flowrates (kg/batch)				
Ammonium Chlori	11,288.37	11,288.37	2.14	53.48
Water	79,455.22	79,455.22	15.10	376.46
TOTAL (kg/batch)	90,743.59	90,743.59	17.24	429.94
TOTAL (L/batch)	86,887.60	87,603.46	16.64	415.07

Stream Name	NH4Cl to SFR-3	NH4Cl to FR-1	Water for NH4SO4	NH4SO4
**Source**	**P-5**	**P-5**	**INPUT**	**INPUT**
**Destination**	**P-65**	**P-4**	**P-36**	**P-36**
Stream Properties				
Activity (U/ml)	0.00	0.00	0.00	0.00
Temperature (°C)	35.00	35.00	10.00	20.00
Pressure (bar)	1.01	1.01	1.01	1.01
Density (g/L)	1,035.84	1,035.84	1,000.17	1,769.00
Total Enthalpy (kW-h)	161.77	3,235.39	1,059.21	2.25
Specific Enthalpy (kcal/kg)	32.37	32.37	10.07	6.80
Heat Capacity (kcal/kg-°C)	0.92	0.92	1.01	0.34
Component Flowrates (kg/batch)				
Amm. Sulfate	0.00	0.00	0.00	284.55
Ammonium Chlori	534.89	10,697.85	0.00	0.00
Water	3,764.91	75,298.76	90,459.38	0.00
TOTAL (kg/batch)	4,299.79	85,996.61	90,459.38	284.55
TOTAL (L/batch)	4,151.00	83,020.75	90,443.84	160.85

Stream Name	SO4-Solution	S-138	Sulfate to SFR-1	Sulfate to SFR-2
**Source**	**P-36**	**P-35**	**P-6**	**P-6**
**Destination**	**P-35**	**P-6**	**P-16**	**P-64**
Stream Properties				
Activity (U/ml)	0.00	0.00	0.00	0.00
Temperature (°C)	10.01	35.00	35.00	35.00
Pressure (bar)	1.01	1.01	1.01	1.01
Density (g/L)	1,001.53	992.43	992.43	992.43
Total Enthalpy (kW-h)	1,061.45	3,694.29	0.70	17.50
Specific Enthalpy (kcal/kg)	10.06	35.03	35.03	35.03
Heat Capacity (kcal/kg-°C)	1.00	1.00	1.00	1.00
Component Flowrates (kg/batch)				
Amm. Sulfate	284.55	284.55	0.05	1.35
Water	90,459.38	90,459.38	17.19	428.60
TOTAL (kg/batch)	90,743.93	90,743.93	17.24	429.94
TOTAL (L/batch)	90,605.05	91,436.30	17.37	433.23

Stream Name	Sulfate to SFR-3	Sulfate to FR-1	Water for NaH2PO4	NaH2PO4
**Source**	**P-6**	**P-6**	**INPUT**	**INPUT**
**Destination**	**P-65**	**P-4**	**P-34**	**P-34**
Stream Properties				
Activity (U/ml)	0.00	0.00	0.00	0.00
Temperature (°C)	35.00	35.00	10.00	20.00
Pressure (bar)	1.01	1.01	1.01	1.01
Density (g/L)	992.43	992.43	1,000.17	2,040.00
Total Enthalpy (kW-h)	175.05	3,501.03	1,026.74	10.65
Specific Enthalpy (kcal/kg)	35.03	35.03	10.07	3.00
Heat Capacity (kcal/kg-°C)	1.00	1.00	1.01	0.15
Component Flowrates (kg/batch)				
Amm. Sulfate	13.48	269.67	0.00	0.00
NaH2PO4	0.00	0.00	0.00	3,056.94
Water	4,286.33	85,727.27	87,686.65	0.00
TOTAL (kg/batch)	4,299.81	85,996.94	87,686.65	3,056.94
TOTAL (L/batch)	4,332.62	86,653.08	87,671.58	1,498.50

Stream Name	PO4-Solution	S-108	Phosphate to SFR-1	Phosphate to SFR-2
**Source**	**P-34**	**P-33**	**P-2**	**P-2**
**Destination**	**P-33**	**P-2**	**P-16**	**P-64**
Stream Properties				
Activity (U/ml)	0.00	0.00	0.00	0.00
Temperature (°C)	10.05	35.00	35.00	35.00
Pressure (bar)	1.01	1.01	1.01	1.01
Density (g/L)	1,017.63	1,008.53	1,008.53	1,008.53
Total Enthalpy (kW-h)	1,037.39	3,595.88	0.68	17.04
Specific Enthalpy (kcal/kg)	9.84	34.10	34.10	34.10
Heat Capacity (kcal/kg-°C)	0.98	0.97	0.97	0.97
Component Flowrates (kg/batch)				
NaH2PO4	3,056.94	3,056.94	0.58	14.48
Water	87,686.65	87,686.65	16.66	415.46
TOTAL (kg/batch)	90,743.59	90,743.59	17.24	429.94
TOTAL (L/batch)	89,171.72	89,976.20	17.10	426.31

Stream Name	Phosphate to SFR-3	Phosphate to FR-1	Salts to SFR-3	Salts to SFR-2
**Source**	**P-2**	**P-2**	**P-65**	**P-64**
**Destination**	**P-65**	**P-4**	**P-15**	**P-1**
Stream Properties				
Activity (U/ml)	0.00	0.00	0.00	0.00
Temperature (°C)	35.00	35.00	35.00	35.00
Pressure (bar)	1.01	1.01	1.01	1.01
Density (g/L)	1,008.53	1,008.53	1,011.95	1,011.95
Total Enthalpy (kW-h)	170.39	3,407.77	507.21	50.72
Specific Enthalpy (kcal/kg)	34.10	34.10	33.83	33.83
Heat Capacity (kcal/kg-°C)	0.97	0.97	0.96	0.96
Component Flowrates (kg/batch)				
Amm. Sulfate	0.00	0.00	13.48	1.35
Ammonium Chlori	0.00	0.00	534.89	53.48
NaH2PO4	144.85	2,897.03	144.85	14.48
Water	4,154.94	83,099.58	12,206.18	1,220.51
TOTAL (kg/batch)	4,299.79	85,996.61	12,899.40	1,289.83
TOTAL (L/batch)	4,263.43	85,269.36	12,747.05	1,274.60

Stream Name	S-123	S-125	S-112	S-118
**Source**	**INPUT**	**P-25**	**INPUT**	**P-21**
**Destination**	**P-25**	**P-24**	**P-21**	**P-20**
Stream Properties				
Activity (U/ml)	0.00	0.00	0.00	0.00
Temperature (°C)	25.00	35.00	25.00	35.00
Pressure (bar)	1.01	1.01	1.01	1.01
Density (g/L)	994.70	991.06	994.70	991.06
Total Enthalpy (kW-h)	6,901.30	9,647.80	626.48	875.80
Specific Enthalpy (kcal/kg)	25.11	35.10	25.11	35.10
Heat Capacity (kcal/kg-°C)	1.00	1.00	1.00	1.00
Component Flowrates (kg/batch)				
Water	236,490.59	236,490.59	21,467.93	21,467.93
TOTAL (kg/batch)	236,490.59	236,490.59	21,467.93	21,467.93
TOTAL (L/batch)	237,749.63	238,624.05	21,582.22	21,661.60

Stream Name	S-120	S-122	Water for 50% Sucrose	Process Sucrose
**Source**	**INPUT**	**P-23**	**INPUT**	**INPUT**
**Destination**	**P-23**	**P-22**	**P-9**	**P-9**
Stream Properties				
Activity (U/ml)	0.00	0.00	0.00	0.00
Temperature (°C)	25.00	35.00	25.00	25.00
Pressure (bar)	1.01	1.01	1.01	1.01
Density (g/L)	994.70	991.06	994.70	1,509.84
Total Enthalpy (kW-h)	69.78	97.56	6,489.00	1,934.83
Specific Enthalpy (kcal/kg)	25.11	35.10	25.11	7.49
Heat Capacity (kcal/kg-°C)	1.00	1.00	1.00	0.30
Component Flowrates (kg/batch)				
Sucrose	0.00	0.00	0.00	222,362.10
Water	2,391.34	2,391.34	222,362.10	0.00
TOTAL (kg/batch)	2,391.34	2,391.34	222,362.10	222,362.10
TOTAL (L/batch)	2,404.07	2,412.91	223,545.93	147,275.60

Stream Name	S-144	S-106	Batch Sucrose	Fed-Batch Sucrose
**Source**	**P-9**	**P-8**	**Sucrose Storage**	**Sucrose Storage**
**Destination**	**P-8**	**Sucrose Storage**	**P-7**	**P-10**
Stream Properties				
Activity (U/ml)	0.00	0.00	0.00	0.00
Temperature (°C)	25.00	35.00	35.00	35.00
Pressure (bar)	1.01	1.01	1.01	1.01
Density (g/L)	1,199.29	1,195.13	1,195.13	1,195.13
Total Enthalpy (kW-h)	8,423.83	11,780.18	961.48	10,818.71
Specific Enthalpy (kcal/kg)	16.30	22.79	22.79	22.79
Heat Capacity (kcal/kg-°C)	0.65	0.65	0.65	0.65
Component Flowrates (kg/batch)				
Sucrose	222,362.10	222,362.10	18,148.75	204,213.35
Water	222,362.10	222,362.10	18,148.75	204,213.35
TOTAL (kg/batch)	444,724.21	444,724.21	36,297.50	408,426.71
TOTAL (L/batch)	370,821.53	372,112.67	30,371.09	341,741.58

Stream Name	Fed-batch Sugar > SFR-1	Fed-Batch Sugar > SFR-2	Fed-Batch Sugar > SFR-3	Fed-Batch Sugar > FR-1
**Source**	**P-10**	**P-10**	**P-10**	**P-10**
**Destination**	**P-16**	**P-1**	**P-15**	**P-4**
Stream Properties				
Activity (U/ml)	0.00	0.00	0.00	0.00
Temperature (°C)	35.00	35.00	35.00	35.00
Pressure (bar)	1.01	1.01	1.01	1.01
Density (g/L)	1,195.13	1,195.13	1,195.13	1,195.13
Total Enthalpy (kW-h)	0.81	9.64	92.11	10,716.15
Specific Enthalpy (kcal/kg)	22.79	22.79	22.79	22.79
Heat Capacity (kcal/kg-°C)	0.65	0.65	0.65	0.65
Component Flowrates (kg/batch)				
Sucrose	15.32	181.95	1,738.67	202,277.41
Water	15.32	181.95	1,738.67	202,277.41
TOTAL (kg/batch)	30.63	363.91	3,477.34	404,554.82
TOTAL (L/batch)	25.63	304.49	2,909.59	338,501.87

Stream Name	S-110	S-124	S-121	S-127
**Source**	**P-7**	**P-7**	**P-7**	**P-7**
**Destination**	**P-12**	**P-22**	**P-20**	**P-24**
Stream Properties				
Activity (U/ml)	0.00	0.00	0.00	0.00
Temperature (°C)	35.00	35.00	35.00	35.00
Pressure (bar)	1.01	1.01	1.01	1.01
Density (g/L)	1,195.13	1,195.13	1,195.13	1,195.13
Total Enthalpy (kW-h)	0.18	4.56	45.56	911.18
Specific Enthalpy (kcal/kg)	22.79	22.79	22.79	22.79
Heat Capacity (kcal/kg-°C)	0.65	0.65	0.65	0.65
Component Flowrates (kg/batch)				
Sucrose	3.45	85.99	859.96	17,199.35
Water	3.45	85.99	859.96	17,199.35
TOTAL (kg/batch)	6.90	171.98	1,719.92	34,398.71
TOTAL (L/batch)	5.77	143.90	1,439.10	28,782.32

Stream Name	Initial Sugar to FR-1	Initial Sugar to SFR-3	Initial Sugar to SFR-2	S-114
**Source**	**P-24**	**P-20**	**P-22**	**INPUT**
**Destination**	**P-4**	**P-15**	**P-1**	**P-18**
Stream Properties				
Activity (U/ml)	0.00	0.00	0.00	0.00
Temperature (°C)	35.00	35.00	35.00	25.00
Pressure (bar)	1.01	1.01	1.01	1.01
Density (g/L)	1,013.02	1,003.77	1,002.54	994.70
Total Enthalpy (kW-h)	10,558.98	921.36	102.11	2.60
Specific Enthalpy (kcal/kg)	33.54	34.19	34.28	25.11
Heat Capacity (kcal/kg-°C)	0.95	0.97	0.98	1.00
Component Flowrates (kg/batch)				
Sucrose	17,199.35	859.96	85.99	0.00
Water	253,689.94	22,327.89	2,477.33	89.18
TOTAL (kg/batch)	270,889.29	23,187.85	2,563.32	89.18
TOTAL (L/batch)	267,406.37	23,100.70	2,556.81	89.65

Stream Name	S-115	Initial Sugar to SFR-1	Air input	S-153
**Source**	**P-18**	**P-12**	**INPUT**	**P-51**
**Destination**	**P-12**	**P-16**	**P-51**	**P-50**
Stream Properties				
Activity (U/ml)	0.00	0.00	0.00	0.00
Temperature (°C)	35.00	35.00	20.00	40.00
Pressure (bar)	1.01	1.01	1.01	6.01
Density (g/L)	991.06	1,003.36	1.20	6.66
Total Enthalpy (kW-h)	3.64	3.82	4,498.75	8,979.58
Specific Enthalpy (kcal/kg)	35.10	34.22	4.85	9.68
Heat Capacity (kcal/kg-°C)	1.00	0.97	0.24	0.24
Component Flowrates (kg/batch)				
Argon	0.00	0.00	7,339.54	7,339.54
Carb. Dioxide	0.00	0.00	319.11	319.11
Nitrogen	0.00	0.00	622,983.30	622,983.30
Oxygen	0.00	0.00	167,134.08	167,134.08
Sucrose	0.00	3.45	0.00	0.00
Water	89.18	92.63	0.00	0.00
TOTAL (kg/batch)	89.18	96.07	797,776.03	797,776.03
TOTAL (L/batch)	89.98	95.75	665,194,975.60	119,734,369.11

Stream Name	S-139	S-148	S-147	S-146
**Source**	**P-50**	**P-41**	**P-41**	**P-41**
**Destination**	**P-41**	**P-16**	**P-1**	**P-15**
Stream Properties				
Activity (U/ml)	0.00	0.00	0.00	0.00
Temperature (°C)	40.00	40.00	40.00	40.00
Pressure (bar)	6.01	6.01	6.01	6.01
Density (g/L)	6.66	6.66	6.66	6.66
Total Enthalpy (kW-h)	8,979.58	0.89	20.58	206.12
Specific Enthalpy (kcal/kg)	9.68	9.68	9.68	9.68
Heat Capacity (kcal/kg-°C)	0.24	0.24	0.24	0.24
Component Flowrates (kg/batch)				
Argon	7,339.54	0.73	16.82	168.47
Carb. Dioxide	319.11	0.03	0.73	7.32
Nitrogen	622,983.30	61.67	1,427.64	14,300.03
Oxygen	167,134.08	16.54	383.01	3,836.41
TOTAL (kg/batch)	797,776.03	78.97	1,828.19	18,312.24
TOTAL (L/batch)	119,734,369.11	11,852.56	274,384.84	2,748,395.72

Stream Name	S-143	Vent SFR-1	Inoculum to SFR-2	Vent FR-1
**Source**	**P-41**	**P-16**	**P-16**	**P-4**
**Destination**	**P-4**	**OUTPUT**	**P-1**	**P-49**
Stream Properties				
Activity (U/ml)	0.00	0.00	0.00	0.00
Temperature (°C)	40.00	35.00	35.00	34.98
Pressure (bar)	6.01	1.01	1.01	1.01
Density (g/L)	6.66	1.20	993.91	1.20
Total Enthalpy (kW-h)	8,751.99	1.71	6.74	16,206.57
Specific Enthalpy (kcal/kg)	9.68	15.98	35.10	15.51
Heat Capacity (kcal/kg-°C)	0.24	0.24	1.00	0.24
Component Flowrates (kg/batch)				
Amm. Sulfate	0.00	0.00	0.00	0.00
Argon	7,153.52	0.73	0.00	7,172.27
Biomass	0.00	0.00	8.44	0.00
Carb. Dioxide	311.02	13.12	0.00	120,003.83
NaH2PO4	0.00	0.00	0.00	0.00
Nitrogen	607,193.97	61.83	0.00	608,785.58
Oxygen	162,898.11	16.59	0.00	163,325.11
Sucrose	0.00	0.00	0.00	0.00
Water	0.00	0.00	156.89	0.00
TOTAL (kg/batch)	777,556.62	92.26	165.33	899,286.80
TOTAL (L/batch)	116,699,735.99	76,915.63	166.35	752,009,974.42

Stream Name	Emissions	Vent SFR-2	Inoculum to SFR-3	Vent SFR-3
**Source**	**P-49**	**P-1**	**P-1**	**P-15**
**Destination**	**OUTPUT**	**OUTPUT**	**P-15**	**OUTPUT**
Stream Properties				
Activity (U/ml)	0.00	0.00	0.00	0.00
Temperature (°C)	34.98	35.00	35.00	35.00
Pressure (bar)	1.01	1.01	1.01	1.01
Density (g/L)	1.20	1.18	992.80	1.18
Total Enthalpy (kW-h)	16,206.57	33.49	169.92	332.11
Specific Enthalpy (kcal/kg)	15.51	14.06	35.10	13.95
Heat Capacity (kcal/kg-°C)	0.24	0.24	1.00	0.24
Component Flowrates (kg/batch)				
Ammonium Chlori	0.00	0.00	0.03	0.00
Argon	7,172.27	16.87	0.00	168.94
Biomass	0.00	0.00	128.94	0.00
Carb. Dioxide	120,003.83	217.16	0.00	2,128.53
NaH2PO4	0.00	0.00	0.01	0.00
Nitrogen	608,785.58	1,431.60	0.00	14,339.59
Oxygen	163,325.11	384.07	0.00	3,847.03
Sucrose	0.00	0.00	0.17	0.00
Water	0.00	0.00	4,036.68	0.00
TOTAL (kg/batch)	899,286.80	2,049.69	4,165.83	20,484.09
TOTAL (L/batch)	752,009,974.42	1,731,162.84	4,196.04	17,313,369.52

Stream Name	Inoculum to FR-1	Mother Liquor	S-116	S-128
**Source**	**P-15**	**P-11**	**P-27**	**P-4**
**Destination**	**P-4**	**P-4**	**P-4**	**OUTPUT**
Stream Properties				
Activity (U/ml)	0.00	0.00	0.00	0.00
Temperature (°C)	35.00	9.00	35.31	12.55
Pressure (bar)	1.01	1.01	1.01	1.01
Density (g/L)	992.80	1,003.90	1,012.42	1,004.90
Total Enthalpy (kW-h)	1,697.24	8,324.82	5,087.67	13,417.76
Specific Enthalpy (kcal/kg)	35.10	9.00	34.37	12.50
Heat Capacity (kcal/kg-°C)	1.00	1.00	0.97	0.99
Component Flowrates (kg/batch)				
Amm. Sulfate	0.00	6.27	0.37	6.64
Ammonium Chlori	0.02	248.83	14.68	263.51
Biomass	1,298.33	0.00	25,321.47	25,321.47
NaH2PO4	0.00	67.36	3.97	71.34
pHBA (aq)	0.00	1,584.14	4,672.17	6,256.31
pHBA (solid)	0.00	1,552.46	0.00	1,552.46
Sucrose	0.18	5,103.23	301.02	5,404.25
Water	40,309.42	787,593.98	97,033.73	884,627.71
TOTAL (kg/batch)	41,607.95	796,156.27	127,347.41	923,503.69
TOTAL (L/batch)	41,909.70	793,061.60	125,784.87	919,002.18

Stream Name	S-113	S-105	Vent R-101	S-101
**Source**	**P-4**	**P-27**	**P-28**	**P-28**
**Destination**	**P-27**	**P-28**	**OUTPUT**	**P-11**
Stream Properties				
Activity (U/ml)	0.00	0.00	0.00	0.00
Temperature (°C)	35.00	35.31	5.00	5.00
Pressure (bar)	1.01	1.01	1.01	1.01
Density (g/L)	1,020.11	1,021.32	1.26	1,033.72
Total Enthalpy (kW-h)	32,221.46	27,418.87	1.18	3,904.73
Specific Enthalpy (kcal/kg)	32.41	32.41	1.23	4.61
Heat Capacity (kcal/kg-°C)	0.92	0.91	0.24	0.92
Component Flowrates (kg/batch)				
Amm. Sulfate	6.64	6.27	0.00	6.27
Ammonium Chlori	263.51	248.83	0.00	248.83
Argon	0.00	0.00	7.57	0.00
Biomass	25,321.47	0.00	0.00	0.00
Carb. Dioxide	0.00	0.00	0.33	0.00
NaH2PO4	71.34	67.36	0.00	67.36
Nitrogen	0.00	0.00	642.69	0.00
Oxygen	0.00	0.00	172.42	0.00
pHBA (aq)	83,879.10	79,206.93	0.00	1,584.14
pHBA (solid)	0.00	0.00	0.00	77,622.79
Sucrose	5,404.25	5,103.23	0.00	5,103.23
Water	740,402.39	643,368.66	0.00	643,368.66
TOTAL (kg/batch)	855,348.69	728,001.28	823.01	728,001.28
TOTAL (L/batch)	838,485.37	712,801.57	651,125.15	704,252.88

Stream Name	Wash Water	S-102	Humid Air	Final Product
Source	INPUT	P-11	P-14	P-14
Destination	P-11	P-14	OUTPUT	OUTPUT
Stream Properties				
Activity (U/ml)	0.00	0.00	0.00	0.00
Temperature (°C)	25.00	22.93	50.00	50.00
Pressure (bar)	1.01	1.86	1.01	1.01
Density (g/L)	994.70	1,211.06	1.08	1,303.70
Total Enthalpy (kW-h)	5,149.97	1,407.79	45,870.09	1,209.42
Specific Enthalpy (kcal/kg)	25.11	11.18	24.28	13.61
Heat Capacity (kcal/kg-°C)	1.00	0.49	0.25	0.27
Component Flowrates (kg/batch)				
Amm. Sulfate	0.00	0.00	0.00	0.00
Ammonium Chlori	0.00	0.00	0.00	0.00
Argon	0.00	0.00	14,659.84	0.00
Carb. Dioxide	0.00	0.00	637.38	0.00
NaH2PO4	0.00	0.00	0.00	0.00
Nitrogen	0.00	0.00	1,244,333.40	0.00
Oxygen	0.00	0.00	333,830.00	0.00
pHBA (aq)	0.00	0.00	0.00	0.00
pHBA (solid)	0.00	76,070.33	0.00	76,070.33
Sucrose	0.00	0.00	0.00	0.00
Water	176,476.80	32,251.48	31,869.21	382.26
TOTAL (kg/batch)	176,476.80	108,321.81	1,625,329.84	76,452.60
TOTAL (L/batch)	177,416.34	89,443.43	1,511,524,267.60	58,642.66

4. OVERALL COMPONENT BALANCE (kg/batch)

COMPONENT	INITIAL	INPUT	OUTPUT	FINAL	IN-OUT
Amm. Sulfate	0.00	284.55	6.64	0.00	277.91
Ammonium Chlori	0.00	11,288.37	263.51	0.00	11,024.86
Argon	21.98	21,999.38	22,026.21	14.35	- 19.21
Biomass	0.00	0.00	25,321.47	0.00	- 25,321.47
Carb. Dioxide	0.96	956.49	123,000.36	3.58	- 122,046.49
NaH2PO4	0.00	3,056.94	71.34	0.00	2,985.61
Nitrogen	1,865.54	1,867,316.70	1,869,594.69	1,217.73	- 1,630.18
Oxygen	500.49	500,964.08	501,575.22	326.69	- 437.34
pHBA (aq)	0.00	0.00	6,256.31	0.00	- 6,256.31
pHBA (solid)	0.00	0.00	77,622.79	0.00	- 77,622.79
Phosphoric Acid	0.00	415.51	415.51	0.00	0.00
Sodium Hydroxid	0.00	633.65	633.65	0.00	0.00
Sucrose	0.00	222,362.10	5,404.25	0.00	216,957.85
Water	0.00	1,006,467.69	1,006,467.69	0.00	0.00
TOTAL	2,388.96	3,635,745.47	3,638,659.64	1,562.35	2,087.56
				Overall Error:	0,057%

5. EQUIPMENT CONTENTS

SFR-3

Procedure	Operation	Time (in h)	Volume (in L)	Vapor (in kg)
P-15	START	25.61	0.00	61.77(*)
P-15	TRANSFER-IN-SALTS (Transfer In)	26.61	12,747.01	61.77(*)
P-15	TRANSFER-IN-INITIAL-SUGAR (Transfer In)	27.61	35,847.69	61.77(*)
P-15	TRANSFER-IN-INOCULUM (Transfer In)	28.11	40,043.76	61.77(*)
P-15	FERMENT-2 (Batch Stoich. Fermentation)	40.11	41,909.70	12.40(*)
P-15	TRANSFER-OUT-1 (Transfer Out)	41.11	0.00	12.40(*)
P-15	CIP-1 (In-Place-Cleaning)	43.19	0.00	12.40(*)
P-15	SIP-1 (In-Place-Steaming)	45.19	0.00	12.40(*)

(*) Contains material in vapor phase other than Oxygen & Nitrogen

SFR-2

Procedure	Operation	Time (in h)	Volume (in L)	Vapor (in kg)
P-1	START	14.11	0.00	6.18(*)
P-1	TRANSFER-IN-SALTS (Transfer In)	14.61	1,274.59	6.18(*)
P-1	TRANSFER-IN-INITIAL-SUGAR (Transfer In)	15.11	3,831.40	6.18(*)
P-1	TRANSFER-IN-INOCULUM (Transfer In)	15.61	3,997.75	6.18(*)
P-1	FERMENT-1 (Batch Stoich. Fermentation)	27.61	4,196.04	1.24(*)
P-1	TRANSFER-OUT-1 (Transfer Out)	28.11	0.00	1.24(*)
P-1	CIP-1 (In-Place-Cleaning)	30.19	0.00	1.24(*)
P-1	SIP-1 (In-Place-Steaming)	31.19	0.00	1.24(*)

(*) Contains material in vapor phase other than Oxygen & Nitrogen

SFR-1

Procedure	Operation	Time (in h)	Volume (in L)	Vapor (in kg)
P-16	START	0.00	0.00	0.25(*)
P-16	TRANSFER-IN-PHOSPHATE (Transfer In)	0.25	17.10	0.25(*)
P-16	TRANSFER-IN-SULFATE (Transfer In)	0.50	34.47	0.25(*)
P-16	TRANSFER-IN-NH4Cl (Transfer In)	0.75	51.11	0.25(*)
P-16	TRANSFER-IN-INITIAL-SUGAR (Transfer In)	1.00	146.87	0.25(*)
P-16	FERMENT (Batch Stoich. Fermentation)	15.11	166.35	0.05(*)
P-16	TRANSFER-OUT (Transfer Out)	15.61	0.00	0.05(*)
P-16	CIP-1 (In-Place-Cleaning)	17.69	0.00	0.05(*)
P-16	SIP-1 (In-Place-Steaming)	18.19	0.00	0.05(*)

(*) Contains material in vapor phase other than Oxygen & Nitrogen

FR-1				
**Procedure**	**Operation**	**Time (in h)**	**Volume (in L)**	**Vapor (in kg)**
P-4	START	39.11	0.00	1,156.42(*)
P-4	TRANSFER-IN-SULFATE (Transfer In)	40.11	86,652.16	1,156.42(*)
P-4	TRANSFER-IN-NH4Cl (Transfer In)	40.11	169,672.96	1,156.42(*)
P-4	TRANSFER-IN-PHOSPHATE (Transfer In)	40.11	254,942.14	1,156.42(*)
P-4	TRANSFER-IN-INITIAL-SUGAR (Transfer In)	40.11	522,348.47	1,156.42(*)
P-4	TRANSFER-IN-INOCULUM (Transfer In)	41.11	564,258.68	1,156.42(*)
P-4	FERMENT-1 (Batch Stoich. Fermentation)	76.27	838,485.37	170.04(*)
P-4	TRANSFER-OUT-2 (Transfer Out)	76.11	0.00	1,126.70(*)
P-4	TRANSFER-IN-1 (Transfer In)	76.11	125,784.86	981.20(*)
P-4	TRANSFER-IN-2 (Transfer In)	76.11	919,002.18	76.43(*)
P-4	TRANSFER-OUT-1 (Transfer Out)	78.27	0.00	1,207.34(*)
P-4	CIP-1 (In-Place-Cleaning)	80.35	0.00	1,207.34(*)
P-4	SIP-1 (In-Place-Steaming)	82.35	0.00	1,207.34(*)

(*) Contains material in vapor phase other than Oxygen & Nitrogen

R-102				
**Procedure**	**Operation**	**Time (in h)**	**Volume (in L)**	**Vapor (in kg)**
P-28	START	40.11	0.00	155.65(*)
	AFTER AUTO-INIT	40.11	118,800.26	155.65(*)
P-28	REACT-1 (Batch Stoich. Reaction)	76.11	117,375.48	18.49(*)
P-28	END	76.11	0.00	18.49(*)

(*) Contains material in vapor phase other than Oxygen & Nitrogen

BCFBD-101				
**Procedure**	**Operation**	**Time (in h)**	**Volume (in L)**	**Vapor (in kg)**
P-11	START	40.11	0.00	19.20(*)
P-11	FILTER-1 (Cloth Filtration)	75.61	7,392.35	19.20(*)
P-11	CAKE-WASH-1 (Cake Wash)	75.86	7,453.62	19.20(*)
P-11	TRANSFER-OUT-1 (Transfer Out)	76.11	0.00	19.20(*)

(*) Contains material in vapor phase other than Oxygen & Nitrogen
