# Supplementary material for: Economic Process Evaluation and Environmental Life-Cycle Assessment of Bio-Aromatics Production
Source: Front Bioeng Biotechnol. 2020 May 13;8:403. doi: 10.3389/fbioe.2020.00403 (PMC7237583; doi:10.3389/fbioe.2020.00403)
Supplement: Supplementary file 1 [file Data_Sheet_1.zip › Sc_13.pdf]

Materials & Streams Report

März 21, 2020

for Supplementary_13_bacterial_best_case_cane_sugar_upscaled

1. OVERALL PROCESS DATA

Annual Operating Time	7,916.60	h
Unit Production Ref. Rate	50,000,000.00	kg MP/yr
Batch Size	77,041.60	kg MP
Recipe Batch Time	140.60	h
Recipe Cycle Time	12.00	h
Number of Batches per Year	649.00	

MP = Total Flow of Stream 'Final Product'

2.1 STARTING MATERIAL REQUIREMENTS (per Section)

Section	Starting Material	Active Product	Amount Needed (kg Sin/kg MP)	Molar Yield (%)	Mass Yield (%)	Gross Mass Yield (%)
Fermentation Section	(none)	(none)	0.00	Unknown	Unknown	Unknown
Downstream Section	(none)	(none)	0.00	Unknown	Unknown	Unknown

Sin = Section Starting Material, Aout = Section Active Product

2.2 BULK MATERIALS (Entire Process)

Material	kg/yr	kg/batch	kg/kg MP
Air	1,966,535,144	3,030,100.38	39.33
Amm. Sulfate	178,208	274.59	0.00
Ammonium Chlori	7,069,643	10,893.13	0.14
Ca Hydroxide	14,164,607	21,825.28	0.28
H3PO4 (2%)	13,449,960	20,724.13	0.27
HNO3 (70%)	35,237,580	54,295.19	0.70
NaH2PO4	1,914,493	2,949.91	0.04
NaOH (0.5 M)	20,983,951	32,332.74	0.42
Sucrose	139,260,239	214,576.64	2.79
Water	724,222,479	1,115,905.21	14.48
TOTAL	2,923,016,304	4,503,877.20	58.46

2.3 BULK MATERIALS (per Section)

SECTIONS IN: Main Branch

Fermentation Section

Material	kg/yr	kg/batch	kg/kg MP
Air	1,003,519,172	1,546,254.50	20.07
Amm. Sulfate	178,208	274.59	0.00
Ammonium Chlori	7,069,643	10,893.13	0.14
Ca Hydroxide	14,164,607	21,825.28	0.28
H3PO4 (2%)	13,449,960	20,724.13	0.27
NaH2PO4	1,914,493	2,949.91	0.04
NaOH (0.5 M)	20,983,951	32,332.74	0.42
Sucrose	139,260,239	214,576.64	2.79
Water	488,022,730	751,961.06	9.76
TOTAL	1,688,563,002	2,601,791.99	33.77

Downstream Section

Material	kg/yr	kg/batch	kg/kg MP
Air	963,015,972	1,483,845.87	19.26
HNO3 (70%)	35,237,580	54,295.19	0.70
Water	236,199,750	363,944.14	4.72
TOTAL	1,234,453,302	1,902,085.21	24.69

2.4 BULK MATERIALS (per Material)

Air

Procedure	% Total	kg/yr	kg/batch	kg/kg MP
Fermentation Section (Main Branch)				
P-51	51.03	1,003,519,172	1,546,254.50	20.07
Downstream Section (Main Branch)				
P-27	48.97	963,015,972	1,483,845.87	19.26
TOTAL	100.00	1,966,535,144	3,030,100.38	39.33

Amm. Sulfate

Procedure	% Total	kg/yr	kg/batch	kg/kg MP
Fermentation Section (Main Branch)				
P-36	100.00	178,208	274.59	0.00
TOTAL	100.00	178,208	274.59	0.00

Ammonium Chlori

Procedure	% Total	kg/yr	kg/batch	kg/kg MP
Fermentation Section (Main Branch)				
P-38	100.00	7,069,643	10,893.13	0.14
TOTAL	100.00	7,069,643	10,893.13	0.14

Ca Hydroxide

Procedure	% Total	kg/yr	kg/batch	kg/kg MP
Fermentation Section (Main Branch)				
P-4	94.38	13,368,456	20,598.54	0.27
P-1	0.51	72,115	111.12	0.00
P-15	5.09	721,154	1,111.18	0.01
P-16	0.02	2,881	4.44	0.00
TOTAL	100.00	14,164,607	21,825.28	0.28

H3PO4 (2%)

Procedure	% Total	kg/yr	kg/batch	kg/kg MP
Fermentation Section (Main Branch)				
P-4	54.66	7,351,129	11,326.85	0.15
P-1	10.06	1,353,104	2,084.91	0.03
P-15	31.85	4,284,409	6,601.55	0.09
P-16	3.43	461,318	710.81	0.01
TOTAL	100.00	13,449,960	20,724.13	0.27

HNO3 (70%)

Procedure	% Total	kg/yr	kg/batch	kg/kg MP
Downstream Section (Main Branch)				
P-3	100.00	35,237,580	54,295.19	0.70
TOTAL	100.00	35,237,580	54,295.19	0.70

NaH2PO4

Procedure	% Total	kg/yr	kg/batch	kg/kg MP
Fermentation Section (Main Branch)				
P-34	100.00	1,914,493	2,949.91	0.04
TOTAL	100.00	1,914,493	2,949.91	0.04

NaOH (0.5 M)

Procedure	% Total	kg/yr	kg/batch	kg/kg MP
Fermentation Section (Main Branch)				
P-4	82.45	17,301,641	26,658.92	0.35
P-1	6.50	1,364,858	2,103.02	0.03
P-15	8.83	1,852,126	2,853.82	0.04
P-16	2.22	465,326	716.99	0.01
TOTAL	100.00	20,983,951	32,332.74	0.42

**Sucrose**				
**Procedure**	**% Total**	**kg/yr**	**kg/batch**	**kg/kg MP**
Fermentation Section (Main Branch)				
P-9	100.00	139,260,239	214,576.64	2.79
TOTAL	100.00	139,260,239	214,576.64	2.79

**Water**				
**Procedure**	**% Total**	**kg/yr**	**kg/batch**	**kg/kg MP**
Fermentation Section (Main Branch)				
P-4	2.24	16,254,039	25,044.74	0.33
P-34	7.58	54,916,117	84,616.51	1.10
P-36	7.82	56,652,616	87,292.17	1.13
P-38	6.87	49,760,966	76,673.29	1.00
P-9	19.23	139,260,239	214,576.64	2.79
P-18	0.01	55,850	86.06	0.00
P-21	1.86	13,444,866	20,716.28	0.27
P-23	0.21	1,497,640	2,307.61	0.03
P-25	20.45	148,108,582	228,210.45	2.96
P-1	0.41	2,991,840	4,609.92	0.06
P-15	0.56	4,059,957	6,255.71	0.08
P-16	0.14	1,020,018	1,571.68	0.02
Downstream Section (Main Branch)				
P-26	17.11	123,948,117	190,983.23	2.48
P-11	15.50	112,251,633	172,960.91	2.25
TOTAL	100.00	724,222,479	1,115,905.21	14.48

2.5 BULK MATERIALS: SECTION TOTALS (kg/kg MP)

**Raw Material**	**Fermentation Section**	**Downstream Section**
Air	20.07	19.26
Amm. Sulfate	0.00	0.00
Ammonium Chlori	0.14	0.00
Ca Hydroxide	0.28	0.00
H3PO4 (2%)	0.27	0.00
HNO3 (70%)	0.00	0.70
NaH2PO4	0.04	0.00
NaOH (0.5 M)	0.42	0.00
Sucrose	2.79	0.00
Water	9.76	4.72
TOTAL	33.77	24.69

2.6 BULK MATERIALS: SECTION TOTALS (kg/batch)

Raw Material	Fermentation Section	Downstream Section
Air	1,546,254.50	1,483,845.87
Amm. Sulfate	274.59	0.00
Ammonium Chlori	10,893.13	0.00
Ca Hydroxide	21,825.28	0.00
H3PO4 (2%)	20,724.13	0.00
HNO3 (70%)	0.00	54,295.19
NaH2PO4	2,949.91	0.00
NaOH (0.5 M)	32,332.74	0.00
Sucrose	214,576.64	0.00
Water	751,961.06	363,944.14
TOTAL	2,601,791.99	1,902,085.21

2.7 BULK MATERIALS: SECTION TOTALS (kg/yr)

Raw Material	Fermentation Section	Downstream Section
Air	1,003,519,172	963,015,972
Amm. Sulfate	178,208	0
Ammonium Chlori	7,069,643	0
Ca Hydroxide	14,164,607	0
H3PO4 (2%)	13,449,960	0
HNO3 (70%)	0	35,237,580
NaH2PO4	1,914,493	0
NaOH (0.5 M)	20,983,951	0
Sucrose	139,260,239	0
Water	488,022,730	236,199,750
TOTAL	1,688,563,002	1,234,453,302

3. STREAM DETAILS

Stream Name	Air for Drying	S-116	Water for NH4Cl	NH4Cl
Source	INPUT	P-27	INPUT	INPUT
Destination	P-27	P-14	P-38	P-38
Stream Properties				
Activity (U/ml)	0.00	0.00	0.00	0.00
Temperature (°C)	25.00	37.66	10.00	20.00
Pressure (bar)	1.01	1.21	1.01	1.01
Density (g/L)	1.18	1.35	1,000.17	1,519.00
Total Enthalpy (kW-h)	10,450.62	15,724.53	897.78	95.15
Specific Enthalpy (kcal/kg)	6.06	9.12	10.07	7.52
Heat Capacity (kcal/kg-°C)	0.24	0.24	1.01	0.38
Component Flowrates (kg/batch)				
Ammonium Chlori	0.00	0.00	0.00	10,893.13
Argon	13,651.38	13,651.38	0.00	0.00
Carb. Dioxide	593.54	593.54	0.00	0.00
Nitrogen	1,158,735.24	1,158,735.24	0.00	0.00
Oxygen	310,865.71	310,865.71	0.00	0.00
Water	0.00	0.00	76,673.29	0.00
TOTAL (kg/batch)	1,483,845.87	1,483,845.87	76,673.29	10,893.13
TOTAL (L/batch)	1,258,350,678.60	1,095,524,080.10	76,660.12	7,171.25

Stream Name	Cl-Solution	S-129	NH4Cl to SFR-1	NH4Cl to SFR-2
Source	P-38	P-37	P-5	P-5
Destination	P-37	P-5	P-16	P-64
Stream Properties				
Activity (U/ml)	0.00	0.00	0.00	0.00
Temperature (°C)	10.50	35.00	35.00	35.00
Pressure (bar)	1.01	1.01	1.01	1.01
Density (g/L)	1,044.38	1,035.84	1,035.84	1,035.84
Total Enthalpy (kW-h)	992.93	3,294.45	0.63	15.61
Specific Enthalpy (kcal/kg)	9.76	32.37	32.37	32.37
Heat Capacity (kcal/kg-°C)	0.93	0.92	0.92	0.92
Component Flowrates (kg/batch)				
Ammonium Chlori	10,893.13	10,893.13	2.07	51.61
Water	76,673.29	76,673.29	14.57	363.28
TOTAL (kg/batch)	87,566.42	87,566.42	16.64	414.89
TOTAL (L/batch)	83,845.45	84,536.24	16.06	400.53

Stream Name	NH4Cl to SFR-3	NH4Cl to FR-1	Water for NH4SO4	NH4SO4
**Source**	**P-5**	**P-5**	**INPUT**	**INPUT**
**Destination**	**P-65**	**P-4**	**P-36**	**P-36**
Stream Properties				
Activity (U/ml)	0.00	0.00	0.00	0.00
Temperature (°C)	35.00	35.00	10.00	20.00
Pressure (bar)	1.01	1.01	1.01	1.01
Density (g/L)	1,035.84	1,035.84	1,000.17	1,769.00
Total Enthalpy (kW-h)	156.10	3,122.11	1,022.12	2.17
Specific Enthalpy (kcal/kg)	32.37	32.37	10.07	6.80
Heat Capacity (kcal/kg-°C)	0.92	0.92	1.01	0.34
Component Flowrates (kg/batch)				
Amm. Sulfate	0.00	0.00	0.00	274.59
Ammonium Chlori	516.16	10,323.29	0.00	0.00
Water	3,633.09	72,662.36	87,292.17	0.00
TOTAL (kg/batch)	4,149.25	82,985.65	87,292.17	274.59
TOTAL (L/batch)	4,005.67	80,113.98	87,277.17	155.22

Stream Name	SO4-Solution	S-138	Sulfate to SFR-1	Sulfate to SFR-2
**Source**	**P-36**	**P-35**	**P-6**	**P-6**
**Destination**	**P-35**	**P-6**	**P-16**	**P-64**
Stream Properties				
Activity (U/ml)	0.00	0.00	0.00	0.00
Temperature (°C)	10.01	35.00	35.00	35.00
Pressure (bar)	1.01	1.01	1.01	1.01
Density (g/L)	1,001.53	992.43	992.43	992.43
Total Enthalpy (kW-h)	1,024.29	3,564.94	0.68	16.89
Specific Enthalpy (kcal/kg)	10.06	35.03	35.03	35.03
Heat Capacity (kcal/kg-°C)	1.00	1.00	1.00	1.00
Component Flowrates (kg/batch)				
Amm. Sulfate	274.59	274.59	0.05	1.30
Water	87,292.17	87,292.17	16.59	413.59
TOTAL (kg/batch)	87,566.76	87,566.76	16.64	414.89
TOTAL (L/batch)	87,432.74	88,234.88	16.76	418.06

Stream Name	Sulfate to SFR-3	Sulfate to FR-1	Water for NaH2PO4	NaH2PO4
**Source**	**P-6**	**P-6**	**INPUT**	**INPUT**
**Destination**	**P-65**	**P-4**	**P-34**	**P-34**
Stream Properties				
Activity (U/ml)	0.00	0.00	0.00	0.00
Temperature (°C)	35.00	35.00	10.00	20.00
Pressure (bar)	1.01	1.01	1.01	1.01
Density (g/L)	992.43	992.43	1,000.17	2,040.00
Total Enthalpy (kW-h)	168.92	3,378.45	990.79	10.28
Specific Enthalpy (kcal/kg)	35.03	35.03	10.07	3.00
Heat Capacity (kcal/kg-°C)	1.00	1.00	1.01	0.15
Component Flowrates (kg/batch)				
Amm. Sulfate	13.01	260.22	0.00	0.00
NaH2PO4	0.00	0.00	0.00	2,949.91
Water	4,136.25	82,725.74	84,616.51	0.00
TOTAL (kg/batch)	4,149.26	82,985.96	84,616.51	2,949.91
TOTAL (L/batch)	4,180.92	83,619.14	84,601.98	1,446.04

Stream Name	PO4-Solution	S-108	Phosphate to SFR-1	Phosphate to SFR-2
**Source**	**P-34**	**P-33**	**P-2**	**P-2**
**Destination**	**P-33**	**P-2**	**P-16**	**P-64**
Stream Properties				
Activity (U/ml)	0.00	0.00	0.00	0.00
Temperature (°C)	10.05	35.00	35.00	35.00
Pressure (bar)	1.01	1.01	1.01	1.01
Density (g/L)	1,017.63	1,008.53	1,008.53	1,008.53
Total Enthalpy (kW-h)	1,001.07	3,469.98	0.66	16.44
Specific Enthalpy (kcal/kg)	9.84	34.10	34.10	34.10
Heat Capacity (kcal/kg-°C)	0.98	0.97	0.97	0.97
Component Flowrates (kg/batch)				
NaH2PO4	2,949.91	2,949.91	0.56	13.98
Water	84,616.51	84,616.51	16.08	400.91
TOTAL (kg/batch)	87,566.42	87,566.42	16.64	414.89
TOTAL (L/batch)	86,049.59	86,825.90	16.50	411.38

Stream Name	Phosphate to SFR-3	Phosphate to FR-1	Salts to SFR-3	Salts to SFR-2
**Source**	**P-2**	**P-2**	**P-65**	**P-64**
**Destination**	**P-65**	**P-4**	**P-15**	**P-1**
Stream Properties				
Activity (U/ml)	0.00	0.00	0.00	0.00
Temperature (°C)	35.00	35.00	35.00	35.00
Pressure (bar)	1.01	1.01	1.01	1.01
Density (g/L)	1,008.53	1,008.53	1,011.95	1,011.95
Total Enthalpy (kW-h)	164.42	3,288.46	489.45	48.94
Specific Enthalpy (kcal/kg)	34.10	34.10	33.83	33.83
Heat Capacity (kcal/kg-°C)	0.97	0.97	0.96	0.96
Component Flowrates (kg/batch)				
Amm. Sulfate	0.00	0.00	13.01	1.30
Ammonium Chlori	0.00	0.00	516.16	51.61
NaH2PO4	139.78	2,795.60	139.78	13.98
Water	4,009.47	80,190.05	11,778.81	1,177.78
TOTAL (kg/batch)	4,149.25	82,985.65	12,447.76	1,244.67
TOTAL (L/batch)	4,114.16	82,283.86	12,300.75	1,229.97

Stream Name	S-123	S-125	S-112	S-118
**Source**	**INPUT**	**P-25**	**INPUT**	**P-21**
**Destination**	**P-25**	**P-24**	**P-21**	**P-20**
Stream Properties				
Activity (U/ml)	0.00	0.00	0.00	0.00
Temperature (°C)	25.00	35.00	25.00	35.00
Pressure (bar)	1.01	1.01	1.01	1.01
Density (g/L)	994.70	991.06	994.70	991.06
Total Enthalpy (kW-h)	6,659.66	9,310.01	604.54	845.14
Specific Enthalpy (kcal/kg)	25.11	35.10	25.11	35.10
Heat Capacity (kcal/kg-°C)	1.00	1.00	1.00	1.00
Component Flowrates (kg/batch)				
Water	228,210.45	228,210.45	20,716.28	20,716.28
TOTAL (kg/batch)	228,210.45	228,210.45	20,716.28	20,716.28
TOTAL (L/batch)	229,425.41	230,269.21	20,826.57	20,903.17

Stream Name	S-120	S-122	Water for 50% Sucrose	Process Sucrose
**Source**	**INPUT**	**P-23**	**INPUT**	**INPUT**
**Destination**	**P-23**	**P-22**	**P-9**	**P-9**
Stream Properties				
Activity (U/ml)	0.00	0.00	0.00	0.00
Temperature (°C)	25.00	35.00	25.00	25.00
Pressure (bar)	1.01	1.01	1.01	1.01
Density (g/L)	994.70	991.06	994.70	1,509.84
Total Enthalpy (kW-h)	67.34	94.14	6,261.80	1,867.09
Specific Enthalpy (kcal/kg)	25.11	35.10	25.11	7.49
Heat Capacity (kcal/kg-°C)	1.00	1.00	1.00	0.30
Component Flowrates (kg/batch)				
Sucrose	0.00	0.00	0.00	214,576.64
Water	2,307.61	2,307.61	214,576.64	0.00
TOTAL (kg/batch)	2,307.61	2,307.61	214,576.64	214,576.64
TOTAL (L/batch)	2,319.90	2,328.43	215,719.02	142,119.11

Stream Name	S-144	S-106	Batch Sucrose	Fed-Batch Sucrose
**Source**	**P-9**	**P-8 Sucrose Storage**	**Sucrose Storage**	**Sucrose Storage**
**Destination**	**P-8 Sucrose Storage**		**P-7**	**P-10**
Stream Properties				
Activity (U/ml)	0.00	0.00	0.00	0.00
Temperature (°C)	25.00	35.00	35.00	35.00
Pressure (bar)	1.01	1.01	1.01	1.01
Density (g/L)	1,199.29	1,195.13	1,195.13	1,195.13
Total Enthalpy (kW-h)	8,128.89	11,367.73	927.81	10,439.92
Specific Enthalpy (kcal/kg)	16.30	22.79	22.79	22.79
Heat Capacity (kcal/kg-°C)	0.65	0.65	0.65	0.65
Component Flowrates (kg/batch)				
Sucrose	214,576.64	214,576.64	17,513.32	197,063.32
Water	214,576.64	214,576.64	17,513.32	197,063.32
TOTAL (kg/batch)	429,153.28	429,153.28	35,026.63	394,126.65
TOTAL (L/batch)	357,838.13	359,084.06	29,307.72	329,776.33

Stream Name	Fed-batch Sugar > SFR-1	Fed-Batch Sugar > SFR-2	Fed-Batch Sugar > SFR-3	Fed-Batch Sugar > FR-1
**Source**	**P-10**	**P-10**	**P-10**	**P-10**
**Destination**	**P-16**	**P-1**	**P-15**	**P-4**
Stream Properties				
Activity (U/ml)	0.00	0.00	0.00	0.00
Temperature (°C)	35.00	35.00	35.00	35.00
Pressure (bar)	1.01	1.01	1.01	1.01
Density (g/L)	1,195.13	1,195.13	1,195.13	1,195.13
Total Enthalpy (kW-h)	0.78	9.30	88.89	10,340.95
Specific Enthalpy (kcal/kg)	22.79	22.79	22.79	22.79
Heat Capacity (kcal/kg-°C)	0.65	0.65	0.65	0.65
Component Flowrates (kg/batch)				
Sucrose	14.78	175.58	1,677.80	195,195.16
Water	14.78	175.58	1,677.80	195,195.16
TOTAL (kg/batch)	29.56	351.17	3,355.59	390,390.32
TOTAL (L/batch)	24.73	293.83	2,807.72	326,650.05

Stream Name	S-110	S-124	S-121	S-127
**Source**	**P-7**	**P-7**	**P-7**	**P-7**
**Destination**	**P-12**	**P-22**	**P-20**	**P-24**
Stream Properties				
Activity (U/ml)	0.00	0.00	0.00	0.00
Temperature (°C)	35.00	35.00	35.00	35.00
Pressure (bar)	1.01	1.01	1.01	1.01
Density (g/L)	1,195.13	1,195.13	1,195.13	1,195.13
Total Enthalpy (kW-h)	0.18	4.40	43.96	879.28
Specific Enthalpy (kcal/kg)	22.79	22.79	22.79	22.79
Heat Capacity (kcal/kg-°C)	0.65	0.65	0.65	0.65
Component Flowrates (kg/batch)				
Sucrose	3.33	82.98	829.85	16,597.16
Water	3.33	82.98	829.85	16,597.16
TOTAL (kg/batch)	6.66	165.96	1,659.70	33,194.32
TOTAL (L/batch)	5.57	138.86	1,388.72	27,774.58

Stream Name	Initial Sugar to FR-1	Initial Sugar to SFR-3	Initial Sugar to SFR-2	S-114
**Source**	**P-24**	**P-20**	**P-22**	**INPUT**
**Destination**	**P-4**	**P-15**	**P-1**	**P-18**
Stream Properties				
Activity (U/ml)	0.00	0.00	0.00	0.00
Temperature (°C)	35.00	35.00	35.00	25.00
Pressure (bar)	1.01	1.01	1.01	1.01
Density (g/L)	1,013.02	1,003.77	1,002.54	994.70
Total Enthalpy (kW-h)	10,189.28	889.10	98.54	2.51
Specific Enthalpy (kcal/kg)	33.54	34.19	34.28	25.11
Heat Capacity (kcal/kg-°C)	0.95	0.97	0.98	1.00
Component Flowrates (kg/batch)				
Sucrose	16,597.16	829.85	82.98	0.00
Water	244,807.61	21,546.13	2,390.59	86.06
TOTAL (kg/batch)	261,404.77	22,375.98	2,473.57	86.06
TOTAL (L/batch)	258,043.79	22,291.89	2,467.29	86.51

Stream Name	S-115	Initial Sugar to SFR-1	Air input	S-153
**Source**	**P-18**	**P-12**	**INPUT**	**P-51**
**Destination**	**P-12**	**P-16**	**P-51**	**P-50**
Stream Properties				
Activity (U/ml)	0.00	0.00	0.00	0.00
Temperature (°C)	35.00	35.00	20.00	40.00
Pressure (bar)	1.01	1.01	1.01	6.01
Density (g/L)	991.06	1,003.36	1.20	6.66
Total Enthalpy (kW-h)	3.51	3.69	8,719.51	17,404.28
Specific Enthalpy (kcal/kg)	35.10	34.22	4.85	9.68
Heat Capacity (kcal/kg-°C)	1.00	0.97	0.24	0.24
Component Flowrates (kg/batch)				
Argon	0.00	0.00	14,225.54	14,225.54
Carb. Dioxide	0.00	0.00	618.50	618.50
Nitrogen	0.00	0.00	1,207,470.14	1,207,470.14
Oxygen	0.00	0.00	323,940.32	323,940.32
Sucrose	0.00	3.33	0.00	0.00
Water	86.06	89.38	0.00	0.00
TOTAL (kg/batch)	86.06	92.71	1,546,254.50	1,546,254.50
TOTAL (L/batch)	86.83	92.40	1,289,285,077.65	232,069,905.87

Stream Name	S-139	S-148	S-147	S-146
**Source**	**P-50**	**P-41**	**P-41**	**P-41**
**Destination**	**P-41**	**P-16**	**P-1**	**P-15**
Stream Properties				
Activity (U/ml)	0.00	0.00	0.00	0.00
Temperature (°C)	40.00	40.00	40.00	40.00
Pressure (bar)	6.01	6.01	6.01	6.01
Density (g/L)	6.66	6.66	6.66	6.66
Total Enthalpy (kW-h)	17,404.28	0.86	19.87	199.16
Specific Enthalpy (kcal/kg)	9.68	9.68	9.68	9.68
Heat Capacity (kcal/kg-°C)	0.24	0.24	0.24	0.24
Component Flowrates (kg/batch)				
Argon	14,225.54	0.70	16.24	162.78
Carb. Dioxide	618.50	0.03	0.71	7.08
Nitrogen	1,207,470.14	59.51	1,378.36	13,817.22
Oxygen	323,940.32	15.97	369.79	3,706.89
TOTAL (kg/batch)	1,546,254.50	76.21	1,765.09	17,693.96
TOTAL (L/batch)	232,069,905.87	11,437.57	264,913.76	2,655,602.03

Stream Name	S-143	Base to SFR-1	S-133	Inoculum to SFR-2
**Source**	**P-41**	**INPUT**	**P-16**	**P-16**
**Destination**	**P-4**	**P-16**	**P-32**	**P-1**
Stream Properties				
Activity (U/ml)	0.00	0.00	0.00	0.00
Temperature (°C)	40.00	25.00	35.00	34.92
Pressure (bar)	6.01	1.01	1.01	1.06
Density (g/L)	6.66	2,329.54	1.20	1,009.48
Total Enthalpy (kW-h)	17,184.39	0.04	1.65	6.54
Specific Enthalpy (kcal/kg)	9.68	7.12	15.98	34.34
Heat Capacity (kcal/kg-°C)	0.24	0.28	0.24	0.98
Component Flowrates (kg/batch)				
Amm. Sulfate	0.00	0.00	0.00	0.00
Argon	14,045.82	0.00	0.70	0.00
Biomass	0.00	0.00	0.00	8.15
Ca Hydroxide	0.00	4.44	0.00	4.44
Carb. Dioxide	610.69	0.00	12.66	0.00
NaH2PO4	0.00	0.00	0.00	0.00
Nitrogen	1,192,215.06	0.00	59.66	0.00
Oxygen	319,847.68	0.00	16.01	0.00
Sucrose	0.00	0.00	0.00	0.00
Water	0.00	0.00	0.00	151.39
TOTAL (kg/batch)	1,526,719.24	4.44	89.03	163.98
TOTAL (L/batch)	229,137,952.50	1.91	74,222.68	162.44

Stream Name	Vent SFR-1	S-131	Vent SFR-2	S-119
**Source**	**P-32**	**P-1**	**P-29**	**P-15**
**Destination**	**OUTPUT**	**P-29**	**OUTPUT**	**P-28**
Stream Properties				
Activity (U/ml)	0.00	0.00	0.00	0.00
Temperature (°C)	35.00	35.00	35.00	35.00
Pressure (bar)	1.01	1.01	1.01	1.01
Density (g/L)	1.20	1.18	1.18	1.18
Total Enthalpy (kW-h)	1.65	32.32	32.32	320.70
Specific Enthalpy (kcal/kg)	15.98	14.05	14.05	13.94
Heat Capacity (kcal/kg-°C)	0.24	0.24	0.24	0.24
Component Flowrates (kg/batch)				
Argon	0.70	16.28	16.28	163.24
Carb. Dioxide	12.66	209.55	209.55	2,053.94
Nitrogen	59.66	1,382.19	1,382.19	13,855.50
Oxygen	16.01	370.81	370.81	3,717.16
TOTAL (kg/batch)	89.03	1,978.84	1,978.84	19,789.83
TOTAL (L/batch)	74,222.68	1,671,347.73	1,671,347.73	16,727,314.65

Stream Name	Vent SFR-3	Vent FR-1	Emissions	S-117
**Source**	**P-28**	**P-4**	**P-49**	**P-26**
**Destination**	**OUTPUT**	**P-49**	**OUTPUT**	**P-30**
Stream Properties				
Activity (U/ml)	0.00	0.00	0.00	0.00
Temperature (°C)	35.00	35.00	35.00	35.31
Pressure (bar)	1.01	1.01	1.01	1.01
Density (g/L)	1.18	1.17	1.17	995.45
Total Enthalpy (kW-h)	320.70	23,320.91	23,320.91	25,012.29
Specific Enthalpy (kcal/kg)	13.94	12.21	12.21	35.14
Heat Capacity (kcal/kg-°C)	0.24	0.24	0.24	0.99
Component Flowrates (kg/batch)				
Amm. Sulfate	0.00	0.00	0.00	4.73
Ammonium Chlori	0.00	0.00	0.00	187.51
Argon	163.24	14,054.54	14,054.54	0.00
Ca Hydroxide	0.00	0.00	0.00	10.56
Carb. Dioxide	2,053.94	116,645.03	116,645.03	0.00
NaH2PO4	0.00	0.00	0.00	50.76
Nitrogen	13,855.50	1,192,955.61	1,192,955.61	0.00
Oxygen	3,717.16	320,046.36	320,046.36	0.00
pHBA Salt	0.00	0.00	0.00	2,026.26
Sucrose	0.00	0.00	0.00	3,845.38
Water	0.00	0.00	0.00	606,255.15
TOTAL (kg/batch)	19,789.83	1,643,701.54	1,643,701.54	612,380.36
TOTAL (L/batch)	16,727,314.65	1,405,631,548.73	1,405,631,548.73	615,179.44

Stream Name	Purge	S-111	Nitric Acid 70%	S-103
Source	P-30	P-30	INPUT	P-31
Destination	OUTPUT	P-17	P-3	P-3
Stream Properties				
Activity (U/ml)	0.00	0.00	0.00	0.00
Temperature (°C)	35.31	35.31	25.00	35.31
Pressure (bar)	1.01	1.01	1.01	1.01
Density (g/L)	995.45	995.45	1,355.32	1,192.31
Total Enthalpy (kW-h)	14,122.24	10,890.05	936.08	11,680.29
Specific Enthalpy (kcal/kg)	35.14	35.14	14.83	27.50
Heat Capacity (kcal/kg-°C)	0.99	0.99	0.59	0.78
Component Flowrates (kg/batch)				
Amm. Sulfate	2.67	2.06	0.00	2.13
Ammonium Chlori	105.87	81.64	0.00	84.29
Ca Hydroxide	5.96	4.60	0.00	4.75
NaH2PO4	28.66	22.10	0.00	22.82
Nitric Acid	0.00	0.00	38,006.64	0.00
pHBA Salt	1,144.05	882.21	0.00	91,140.27
Sucrose	2,171.15	1,674.23	0.00	1,728.54
Water	342,298.94	263,956.21	16,288.56	272,517.35
TOTAL (kg/batch)	345,757.31	266,623.05	54,295.19	365,500.14
TOTAL (L/batch)	347,337.70	267,841.74	40,060.79	306,547.14

Stream Name	S-102	S-104	Base to SFR-2	Inoculum to SFR-3
**Source**	**P-3**	**P-13**	**INPUT**	**P-1**
**Destination**	**P-13**	**P-11**	**P-1**	**P-15**
Stream Properties				
Activity (U/ml)	0.00	0.00	0.00	0.00
Temperature (°C)	33.67	5.00	25.00	34.92
Pressure (bar)	1.01	1.01	1.01	1.06
Density (g/L)	1,114.42	1,128.18	2,329.54	1,008.87
Total Enthalpy (kW-h)	12,647.02	1,887.52	0.92	164.95
Specific Enthalpy (kcal/kg)	25.92	3.87	7.12	34.32
Heat Capacity (kcal/kg-°C)	0.77	0.77	0.28	0.98
Component Flowrates (kg/batch)				
Amm. Sulfate	2.13	2.13	0.00	0.00
Ammonium Chlori	84.29	84.29	0.00	0.02
Biomass	0.00	0.00	0.00	124.43
Ca Hydroxide	4.75	4.75	111.12	115.56
Calcium Nitrate	47,582.59	47,582.59	0.00	0.00
NaH2PO4	22.82	22.82	0.00	0.01
Nitric Acid	1,461.79	1,461.79	0.00	0.00
pHBA (aq)	80,104.27	1,883.47	0.00	0.00
pHBA (solid)	0.00	78,220.79	0.00	0.00
Sucrose	1,728.54	1,728.54	0.00	0.17
Water	288,805.91	288,805.91	0.00	3,895.35
TOTAL (kg/batch)	419,797.07	419,797.07	111.12	4,135.53
TOTAL (L/batch)	376,694.21	372,101.57	47.70	4,099.19

Stream Name	Base to SFR-3	Inoculum to FR-1	Base to FR-1	S-105
**Source**	**INPUT**	**P-15**	**INPUT**	**P-4**
**Destination**	**P-15**	**P-4**	**P-4**	**P-19**
Stream Properties				
Activity (U/ml)	0.00	0.00	0.00	0.00
Temperature (°C)	25.00	34.92	25.00	35.00
Pressure (bar)	1.01	1.06	1.01	1.01
Density (g/L)	2,329.54	1,009.86	2,329.54	1,073.42
Total Enthalpy (kW-h)	9.19	1,648.36	170.43	31,077.85
Specific Enthalpy (kcal/kg)	7.12	34.28	7.12	31.58
Heat Capacity (kcal/kg-°C)	0.28	0.98	0.28	0.90
Component Flowrates (kg/batch)				
Amm. Sulfate	0.00	0.00	0.00	5.21
Ammonium Chlori	0.00	0.02	0.00	206.55
Biomass	0.00	1,252.87	0.00	24,544.78
Ca Hydroxide	1,111.18	1,226.73	20,598.54	11.63
NaH2PO4	0.00	0.00	0.00	55.91
pHBA Salt	0.00	0.00	0.00	92,530.26
Sucrose	0.00	0.17	0.00	4,235.85
Water	0.00	38,898.09	0.00	725,086.28
TOTAL (kg/batch)	1,111.18	41,377.89	20,598.54	846,676.48
TOTAL (L/batch)	476.99	40,973.88	8,842.33	788,765.60

Stream Name	S-113	RVF Cake	S-107	S-126
**Source**	**P-19**	**P-17**	**P-17**	**P-26**
**Destination**	**P-17**	**OUTPUT**	**P-26**	**P-31**
Stream Properties				
Activity (U/ml)	0.00	0.00	0.00	0.00
Temperature (°C)	35.00	35.12	35.07	35.31
Pressure (bar)	10.47	1.01	1.01	1.01
Density (g/L)	1,073.42	1,003.01	1,061.01	1,191.83
Total Enthalpy (kW-h)	31,078.04	5,479.02	36,489.08	11,722.83
Specific Enthalpy (kcal/kg)	31.58	35.10	32.07	27.51
Heat Capacity (kcal/kg-°C)	0.90	1.00	0.91	0.78
Component Flowrates (kg/batch)				
Amm. Sulfate	5.21	0.41	6.85	2.13
Ammonium Chlori	206.55	16.33	271.87	84.35
Biomass	24,544.78	24,299.33	245.45	245.45
Ca Hydroxide	11.63	0.92	15.31	4.75
NaH2PO4	55.91	4.42	73.59	22.83
pHBA Salt	92,530.26	176.44	93,236.03	91,209.77
Sucrose	4,235.85	334.85	5,575.24	1,729.85
Water	725,086.28	109,489.46	879,553.03	273,297.88
TOTAL (kg/batch)	846,676.48	134,322.16	978,977.37	366,597.01
TOTAL (L/batch)	788,765.66	133,919.23	922,688.17	307,592.60

Stream Name	DEF Cake	Water for Cake Wash	Wastewater	S-101
**Source**	**P-31**	**INPUT**	**P-11**	**P-11**
**Destination**	**OUTPUT**	**P-11**	**OUTPUT**	**P-14**
Stream Properties				
Activity (U/ml)	0.00	0.00	0.00	0.00
Temperature (°C)	35.31	25.00	11.90	22.88
Pressure (bar)	1.01	1.01	1.01	1.77
Density (g/L)	1,049.17	994.70	1,058.55	1,217.13
Total Enthalpy (kW-h)	42.55	5,047.37	6,190.08	1,350.33
Specific Enthalpy (kcal/kg)	33.37	25.11	10.96	10.89
Heat Capacity (kcal/kg-°C)	0.94	1.00	0.92	0.47
Component Flowrates (kg/batch)				
Amm. Sulfate	0.00	0.00	2.13	0.00
Ammonium Chlori	0.06	0.00	84.29	0.00
Biomass	245.45	0.00	0.00	0.00
Ca Hydroxide	0.00	0.00	4.75	0.00
Calcium Nitrate	0.00	0.00	47,582.57	0.01
NaH2PO4	0.02	0.00	22.82	0.00
Nitric Acid	0.00	0.00	1,461.79	0.00
pHBA (aq)	0.00	0.00	1,883.47	0.00
pHBA (solid)	0.00	0.00	1,564.42	76,656.38
pHBA Salt	69.50	0.00	0.00	0.00
Sucrose	1.32	0.00	1,728.54	0.00
Water	780.52	172,960.91	431,704.70	30,062.13
TOTAL (kg/batch)	1,096.87	172,960.91	486,039.47	106,718.52
TOTAL (L/batch)	1,045.46	173,881.73	459,156.13	87,680.75

Stream Name	Humid Air	Final Product
**Source**	**P-14**	**P-14**
**Destination**	**OUTPUT**	**OUTPUT**
Stream Properties		
Activity (U/ml)	0.00	0.00
Temperature (°C)	50.00	50.00
Pressure (bar)	1.01	1.01
Density (g/L)	1.08	1,303.70
Total Enthalpy (kW-h)	42,714.66	1,218.74
Specific Enthalpy (kcal/kg)	24.28	13.61
Heat Capacity (kcal/kg-°C)	0.25	0.27
Component Flowrates (kg/batch)		
Amm. Sulfate	0.00	0.00
Ammonium Chlori	0.00	0.00
Argon	13,651.38	0.00
Ca Hydroxide	0.00	0.00
Calcium Nitrate	0.00	0.01
Carb. Dioxide	593.54	0.00
NaH2PO4	0.00	0.00
Nitric Acid	0.00	0.00
Nitrogen	1,158,735.24	0.00
Oxygen	310,865.71	0.00
pHBA (aq)	0.00	0.00
pHBA (solid)	0.00	76,656.38
Sucrose	0.00	0.00
Water	29,676.92	385.21
TOTAL (kg/batch)	1,513,522.79	77,041.60
TOTAL (L/batch)	1,407,545,947.32	59,094.45

4. OVERALL COMPONENT BALANCE (kg/batch)

COMPONENT	INITIAL	INPUT	OUTPUT	FINAL	IN-OUT
Amm. Sulfate	0.00	274.59	5.21	0.00	269.38
Ammonium Chlori	0.00	10,893.13	206.55	0.00	10,686.58
Argon	23.02	27,876.92	27,886.15	13.80	0.00
Biomass	0.00	0.00	24,544.78	0.00	- 24,544.78
Ca Hydroxide	0.00	21,825.28	11.63	0.00	21,813.65
Calcium Nitrate	0.00	0.00	47,582.59	0.00	- 47,582.59
Carb. Dioxide	1.00	1,212.04	119,514.73	18.33	- 118,320.02
NaH2PO4	0.00	2,949.91	55.91	0.00	2,894.00
Nitric Acid	0.00	38,006.64	1,461.79	0.00	36,544.84
Nitrogen	1,953.93	2,366,205.38	2,366,988.20	1,171.12	0.00
Oxygen	524.20	634,806.03	635,016.04	314.19	- 0.00
pHBA (aq)	0.00	0.00	1,883.47	0.00	- 1,883.47
pHBA (solid)	0.00	0.00	78,220.79	0.00	- 78,220.79
pHBA Salt	0.00	0.00	1,389.99	0.00	- 1,389.99
Phosphoric Acid	0.00	414.48	414.48	0.00	0.00
Sodium Hydroxid	0.00	633.72	633.72	0.00	0.00
Sucrose	0.00	214,576.64	4,235.85	0.00	210,340.79
Water	0.00	1,184,202.43	1,194,809.71	0.00	- 10,607.27
TOTAL	2,502.15	4,503,877.20	4,504,861.60	1,517.43	0.32

5. EQUIPMENT CONTENTS

SFR-3

Procedure	Operation	Time (in h)	Volume (in L)	Vapor (in kg)
P-15	START	25.61	0.00	60.40(*)
P-15	TRANSFER-IN-SALTS (Transfer In)	26.61	12,300.70	60.40(*)
P-15	TRANSFER-IN-INITIAL-SUGAR (Transfer In)	27.61	34,592.57	60.40(*)
P-15	TRANSFER-IN-INOCULUM (Transfer In)	28.11	38,691.76	60.40(*)
P-15	FERMENT-2 (Batch Stoich. Fermentation)	40.11	40,492.52	12.69(*)
P-15	CHARGE-1 (Charge)	40.11	40,973.88	12.69(*)
P-15	TRANSFER-OUT-1 (Transfer Out)	41.11	0.00	12.69(*)
P-15	CIP-1 (In-Place-Cleaning)	43.19	0.00	12.69(*)
P-15	SIP-1 (In-Place-Steaming)	45.19	0.00	12.69(*)

(*) Contains material in vapor phase other than Oxygen & Nitrogen

SFR-2

Procedure	Operation	Time (in h)	Volume (in L)	Vapor (in kg)
P-1	START	14.11	0.00	6.04(*)
P-1	TRANSFER-IN-SALTS (Transfer In)	14.61	1,229.97	6.04(*)
P-1	TRANSFER-IN-INITIAL-SUGAR (Transfer In)	15.11	3,697.25	6.04(*)
P-1	AGITATE-1 (Agitation)	15.11	3,697.32	6.04(*)
P-1	TRANSFER-IN-INOCULUM (Transfer In)	15.61	3,859.76	6.04(*)
P-1	FERMENT-1 (Batch Stoich. Fermentation)	27.61	4,051.05	1.27(*)
P-1	CHARGE-1 (Charge)	27.61	4,099.19	1.27(*)
P-1	TRANSFER-OUT-1 (Transfer Out)	28.11	0.00	1.27(*)
P-1	CIP-1 (In-Place-Cleaning)	30.19	0.00	1.27(*)
P-1	SIP-1 (In-Place-Steaming)	31.19	0.00	1.27(*)

(*) Contains material in vapor phase other than Oxygen & Nitrogen

SFR-1

Procedure	Operation	Time (in h)	Volume (in L)	Vapor (in kg)
P-16	START	0.00	0.00	0.24(*)
P-16	TRANSFER-IN-PHOSPHATE (Transfer In)	0.25	16.50	0.24(*)
P-16	TRANSFER-IN-SULFATE (Transfer In)	0.50	33.26	0.24(*)
P-16	TRANSFER-IN-NH4Cl (Transfer In)	0.75	49.32	0.24(*)
P-16	TRANSFER-IN-INITIAL-SUGAR (Transfer In)	1.00	141.72	0.24(*)
P-16	FERMENT (Batch Stoich. Fermentation)	15.11	160.52	0.05(*)
P-16	CHARGE-1 (Charge)	15.11	162.44	0.05(*)
P-16	TRANSFER-OUT (Transfer Out)	15.61	0.00	0.05(*)
P-16	CIP-1 (In-Place-Cleaning)	17.69	0.00	0.05(*)
P-16	SIP-1 (In-Place-Steaming)	18.19	0.00	0.05(*)

(*) Contains material in vapor phase other than Oxygen & Nitrogen

BCFBD-101

Procedure	Operation	Time (in h)	Volume (in L)	Vapor (in kg)
P-11	START	125.60	0.00	59.84(*)
P-11	FILTER-1 (Cloth Filtration)	137.10	21,735.22	59.84(*)
P-11	CAKE-WASH-1 (Cake Wash)	137.35	21,920.19	59.84(*)
P-11	TRANSFER-OUT-1 (Transfer Out)	137.60	0.00	59.84(*)

(*) Contains material in vapor phase other than Oxygen & Nitrogen

V-102

Procedure	Operation	Time (in h)	Volume (in L)	Vapor (in kg)
P-19	START	112.43	0.00	1,033.46(*)
P-19	TRANSFER-IN-1 (Transfer In)	114.43	788,765.66	1,033.46(*)
P-19	TRANSFER-OUT-1 (Transfer Out)	136.43	0.00	1,033.46(*)

(*) Contains material in vapor phase other than Oxygen & Nitrogen

DE-101

Procedure	Operation	Time (in h)	Volume (in L)	Vapor (in kg)
P-31	START	114.60	0.00	0.00
P-31	FILTER-1 (Dead-End Filtration)	125.60	522.73	0.00
P-31	TRANSFER-OUT-1 (Transfer Out)	126.60	0.00	0.00

FR-1

Procedure	Operation	Time (in h)	Volume (in L)	Vapor (in kg)
P-4	START	39.11	0.00	1,162.64(*)
P-4	TRANSFER-IN-SULFATE (Transfer In)	40.11	83,618.09	1,162.64(*)
P-4	TRANSFER-IN-NH4Cl (Transfer In)	40.11	163,732.14	1,162.64(*)
P-4	TRANSFER-IN-PHOSPHATE (Transfer In)	40.11	246,016.24	1,162.64(*)
P-4	TRANSFER-IN-INITIAL-SUGAR (Transfer In)	40.11	504,060.00	1,162.64(*)
P-4	TRANSFER-IN-INOCULUM (Transfer In)	41.11	545,033.49	1,162.64(*)
P-4	CHARGE-1 (Charge)	112.43	553,956.17	1,162.64(*)
P-4	FERMENT-1 (Batch Stoich. Fermentation)	112.43	788,765.60	230.59(*)
P-4	TRANSFER-OUT-1 (Transfer Out)	114.43	0.00	230.59(*)
P-4	CIP-1 (In-Place-Cleaning)	116.51	0.00	230.59(*)
P-4	SIP-1 (In-Place-Steaming)	118.51	0.00	230.59(*)

(*) Contains material in vapor phase other than Oxygen & Nitrogen
