# Supplementary material for: Economic Process Evaluation and Environmental Life-Cycle Assessment of Bio-Aromatics Production
Source: Front Bioeng Biotechnol. 2020 May 13;8:403. doi: 10.3389/fbioe.2020.00403 (PMC7237583; doi:10.3389/fbioe.2020.00403)
Supplement: Supplementary file 1 [file Data_Sheet_1.zip › Sc_15.pdf]

Materials & Streams Report

for Supplementary_15_yeast_base_case

März 21, 2020

1. OVERALL PROCESS DATA

Annual Operating Time	7,918.51	h
Unit Production Ref. Rate	10,000,000.00	kg MP/yr
Batch Size	15,360.98	kg MP
Recipe Batch Time	118.51	h
Recipe Cycle Time	12.00	h
Number of Batches per Year	651.00	

MP = Total Flow of Stream 'Final Product'

2.1 STARTING MATERIAL REQUIREMENTS (per Section)

Section	Starting Material	Active Product	Amount Needed (kg Sin/kg MP)	Molar Yield (%)	Mass Yield (%)	Gross Mass Yield (%)
Fermentation Section	(none)	(none)	0.00	Unknown	Unknown	Unknown
Downstream Section	(none)	(none)	0.00	Unknown	Unknown	Unknown

Sin = Section Starting Material, Aout = Section Active Product

2.2 BULK MATERIALS (Entire Process)

Material	kg/yr	kg/batch	kg/kg MP
Air	414,430,816	636,606.48	41.44
Amm. Sulfate	37,210	57.16	0.00
Ammonium Chlori	1,476,141	2,267.50	0.15
H3PO4 (2%)	5,928,240	9,106.36	0.59
NaH2PO4	399,746	614.05	0.04
NaOH (0.5 M)	8,170,291	12,550.37	0.82
Sucrose	29,077,524	44,665.94	2.91
Water	129,807,047	199,396.39	12.98
TOTAL	589,327,014	905,264.23	58.93

2.3 BULK MATERIALS (per Section)

SECTIONS IN: Main Branch

Fermentation Section

Material	kg/yr	kg/batch	kg/kg MP
Air	206,000,806	316,437.49	20.60
Amm. Sulfate	37,210	57.16	0.00
Ammonium Chlori	1,476,141	2,267.50	0.15
H3PO4 (2%)	5,928,240	9,106.36	0.59
NaH2PO4	399,746	614.05	0.04
NaOH (0.5 M)	8,170,291	12,550.37	0.82
Sucrose	29,077,524	44,665.94	2.91
Water	106,723,673	163,938.05	10.67
TOTAL	357,813,630	549,636.91	35.78

Downstream Section

Material	kg/yr	kg/batch	kg/kg MP
Air	208,430,010	320,168.99	20.84
Water	23,083,374	35,458.33	2.31
TOTAL	231,513,384	355,627.32	23.15

2.4 BULK MATERIALS (per Material)

Air

Procedure	% Total	kg/yr	kg/batch	kg/kg MP
Fermentation Section (Main Branch)				
P-51	49.71	206,000,806	316,437.49	20.60
Downstream Section (Main Branch)				
P-3	50.29	208,430,010	320,168.99	20.84
TOTAL	100.00	414,430,816	636,606.48	41.44

Amm. Sulfate

Procedure	% Total	kg/yr	kg/batch	kg/kg MP
Fermentation Section (Main Branch)				
P-36	100.00	37,210	57.16	0.00
TOTAL	100.00	37,210	57.16	0.00

Ammonium Chlori

Procedure	% Total	kg/yr	kg/batch	kg/kg MP
Fermentation Section (Main Branch)				
P-38	100.00	1,476,141	2,267.50	0.15
TOTAL	100.00	1,476,141	2,267.50	0.15

H3PO4 (2%)

Procedure	% Total	kg/yr	kg/batch	kg/kg MP
Fermentation Section (Main Branch)				
P-4	45.81	2,715,585	4,171.41	0.27
P-1	8.51	504,661	775.21	0.05
P-15	42.78	2,535,917	3,895.42	0.25
P-16	2.90	172,077	264.33	0.02
TOTAL	100.00	5,928,240	9,106.36	0.59

NaH2PO4

Procedure	% Total	kg/yr	kg/batch	kg/kg MP
Fermentation Section (Main Branch)				
P-34	100.00	399,746	614.05	0.04
TOTAL	100.00	399,746	614.05	0.04

NaOH (0.5 M)

Procedure	% Total	kg/yr	kg/batch	kg/kg MP
Fermentation Section (Main Branch)				
P-4	78.23	6,391,411	9,817.84	0.64
P-1	6.23	509,045	781.94	0.05
P-15	13.42	1,096,263	1,683.97	0.11
P-16	2.12	173,572	266.62	0.02
TOTAL	100.00	8,170,291	12,550.37	0.82

Sucrose

Procedure	% Total	kg/yr	kg/batch	kg/kg MP
Fermentation Section (Main Branch)				
P-9	100.00	29,077,524	44,665.94	2.91
TOTAL	100.00	29,077,524	44,665.94	2.91

Water

Procedure	% Total	kg/yr	kg/batch	kg/kg MP
Fermentation Section (Main Branch)				
P-4	4.63	6,004,415	9,223.37	0.60
P-34	8.83	11,466,480	17,613.64	1.15
P-36	9.11	11,829,061	18,170.60	1.18
P-38	8.00	10,390,085	15,960.19	1.04
P-9	22.40	29,077,524	44,665.94	2.91
P-18	0.01	11,662	17.91	0.00
P-21	2.16	2,807,287	4,312.27	0.28
P-23	0.24	312,707	480.35	0.03
P-25	23.82	30,925,057	47,503.93	3.09
P-1	0.86	1,115,853	1,714.06	0.11

P-15	1.85	2,403,065	3,691.34	0.24
P-16	0.29	380,479	584.45	0.04
Downstream Section (Main Branch)				
P-11	17.78	23,083,374	35,458.33	2.31
TOTAL	100.00	129,807,047	199,396.39	12.98

2.5 BULK MATERIALS: SECTION TOTALS (kg/kg MP)

Raw Material	Fermentation Section	Downstream Section
Air	20.60	20.84
Amm. Sulfate	0.00	0.00
Ammonium Chlori	0.15	0.00
H3PO4 (2%)	0.59	0.00
NaH2PO4	0.04	0.00
NaOH (0.5 M)	0.82	0.00
Sucrose	2.91	0.00
Water	10.67	2.31
TOTAL	35.78	23.15

2.6 BULK MATERIALS: SECTION TOTALS (kg/batch)

Raw Material	Fermentation Section	Downstream Section
Air	316,437.49	320,168.99
Amm. Sulfate	57.16	0.00
Ammonium Chlori	2,267.50	0.00
H3PO4 (2%)	9,106.36	0.00
NaH2PO4	614.05	0.00
NaOH (0.5 M)	12,550.37	0.00
Sucrose	44,665.94	0.00
Water	163,938.05	35,458.33
TOTAL	549,636.91	355,627.32

2.7 BULK MATERIALS: SECTION TOTALS (kg/yr)

Raw Material	Fermentation Section	Downstream Section
Air	206,000,806	208,430,010
Amm. Sulfate	37,210	0
Ammonium Chlori	1,476,141	0
H3PO4 (2%)	5,928,240	0
NaH2PO4	399,746	0
NaOH (0.5 M)	8,170,291	0
Sucrose	29,077,524	0
Water	106,723,673	23,083,374
TOTAL	357,813,630	231,513,384

3. STREAM DETAILS

Stream Name	Air for Drying	S-104	Water for NH4Cl	NH4Cl
Source	INPUT	P-3	INPUT	INPUT
Destination	P-3	P-14	P-38	P-38
Stream Properties				
Activity (U/ml)	0.00	0.00	0.00	0.00
Temperature (°C)	25.00	37.66	10.00	20.00
Pressure (bar)	1.01	1.21	1.01	1.01
Density (g/L)	1.18	1.35	1,000.17	1,519.00
Total Enthalpy (kW-h)	2,254.93	3,392.88	186.88	19.81
Specific Enthalpy (kcal/kg)	6.06	9.12	10.07	7.52
Heat Capacity (kcal/kg-°C)	0.24	0.24	1.01	0.38
Component Flowrates (kg/batch)				
Ammonium Chlori	0.00	0.00	0.00	2,267.50
Argon	2,945.55	2,945.55	0.00	0.00
Carb. Dioxide	128.07	128.07	0.00	0.00
Nitrogen	250,019.96	250,019.96	0.00	0.00
Oxygen	67,075.40	67,075.40	0.00	0.00
Water	0.00	0.00	15,960.19	0.00
TOTAL (kg/batch)	320,168.99	320,168.99	15,960.19	2,267.50
TOTAL (L/batch)	271,513,954.78	236,380,907.64	15,957.45	1,492.76

Stream Name	Cl-Solution	S-129	NH4Cl to SFR-1	NH4Cl to SFR-2
Source	P-38	P-37	P-5	P-5
Destination	P-37	P-5	P-16	P-64
Stream Properties				
Activity (U/ml)	0.00	0.00	0.00	0.00
Temperature (°C)	10.50	35.00	35.00	35.00
Pressure (bar)	1.01	1.01	1.01	1.01
Density (g/L)	1,044.38	1,035.84	1,035.84	1,035.84
Total Enthalpy (kW-h)	206.69	685.77	0.13	3.25
Specific Enthalpy (kcal/kg)	9.76	32.37	32.37	32.37
Heat Capacity (kcal/kg-°C)	0.93	0.92	0.92	0.92
Component Flowrates (kg/batch)				
Ammonium Chlori	2,267.50	2,267.50	0.43	10.74
Water	15,960.19	15,960.19	3.03	75.62
TOTAL (kg/batch)	18,227.69	18,227.69	3.46	86.36
TOTAL (L/batch)	17,453.14	17,596.93	3.34	83.37

Stream Name	NH4Cl to SFR-3	NH4Cl to FR-1	Water for NH4SO4	NH4SO4
**Source**	**P-5**	**P-5**	**INPUT**	**INPUT**
**Destination**	**P-65**	**P-4**	**P-36**	**P-36**
Stream Properties				
Activity (U/ml)	0.00	0.00	0.00	0.00
Temperature (°C)	35.00	35.00	10.00	20.00
Pressure (bar)	1.01	1.01	1.01	1.01
Density (g/L)	1,035.84	1,035.84	1,000.17	1,769.00
Total Enthalpy (kW-h)	32.49	649.89	212.76	0.45
Specific Enthalpy (kcal/kg)	32.37	32.37	10.07	6.80
Heat Capacity (kcal/kg-°C)	0.92	0.92	1.01	0.34
Component Flowrates (kg/batch)				
Amm. Sulfate	0.00	0.00	0.00	57.16
Ammonium Chlori	107.44	2,148.88	0.00	0.00
Water	756.26	15,125.28	18,170.60	0.00
TOTAL (kg/batch)	863.70	17,274.16	18,170.60	57.16
TOTAL (L/batch)	833.81	16,676.40	18,167.48	32.31

Stream Name	SO4-Solution	S-138	Sulfate to SFR-1	Sulfate to SFR-2
**Source**	**P-36**	**P-35**	**P-6**	**P-6**
**Destination**	**P-35**	**P-6**	**P-16**	**P-64**
Stream Properties				
Activity (U/ml)	0.00	0.00	0.00	0.00
Temperature (°C)	10.01	35.00	35.00	35.00
Pressure (bar)	1.01	1.01	1.01	1.01
Density (g/L)	1,001.53	992.43	992.43	992.43
Total Enthalpy (kW-h)	213.21	742.07	0.14	3.52
Specific Enthalpy (kcal/kg)	10.06	35.03	35.03	35.03
Heat Capacity (kcal/kg-°C)	1.00	1.00	1.00	1.00
Component Flowrates (kg/batch)				
Amm. Sulfate	57.16	57.16	0.01	0.27
Water	18,170.60	18,170.60	3.45	86.09
TOTAL (kg/batch)	18,227.76	18,227.76	3.46	86.36
TOTAL (L/batch)	18,199.86	18,366.83	3.49	87.02

Stream Name	Sulfate to SFR-3	Sulfate to FR-1	Water for NaH2PO4	NaH2PO4
**Source**	**P-6**	**P-6**	**INPUT**	**INPUT**
**Destination**	**P-65**	**P-4**	**P-34**	**P-34**
Stream Properties				
Activity (U/ml)	0.00	0.00	0.00	0.00
Temperature (°C)	35.00	35.00	10.00	20.00
Pressure (bar)	1.01	1.01	1.01	1.01
Density (g/L)	992.43	992.43	1,000.17	2,040.00
Total Enthalpy (kW-h)	35.16	703.25	206.24	2.14
Specific Enthalpy (kcal/kg)	35.03	35.03	10.07	3.00
Heat Capacity (kcal/kg-°C)	1.00	1.00	1.01	0.15
Component Flowrates (kg/batch)				
Amm. Sulfate	2.71	54.17	0.00	0.00
NaH2PO4	0.00	0.00	0.00	614.05
Water	861.00	17,220.06	17,613.64	0.00
TOTAL (kg/batch)	863.70	17,274.23	17,613.64	614.05
TOTAL (L/batch)	870.29	17,406.03	17,610.61	301.00

Stream Name	PO4-Solution	S-108	Phosphate to SFR-1	Phosphate to SFR-2
**Source**	**P-34**	**P-33**	**P-2**	**P-2**
**Destination**	**P-33**	**P-2**	**P-16**	**P-64**
Stream Properties				
Activity (U/ml)	0.00	0.00	0.00	0.00
Temperature (°C)	10.05	35.00	35.00	35.00
Pressure (bar)	1.01	1.01	1.01	1.01
Density (g/L)	1,017.63	1,008.53	1,008.53	1,008.53
Total Enthalpy (kW-h)	208.38	722.30	0.14	3.42
Specific Enthalpy (kcal/kg)	9.84	34.10	34.10	34.10
Heat Capacity (kcal/kg-°C)	0.98	0.97	0.97	0.97
Component Flowrates (kg/batch)				
NaH2PO4	614.05	614.05	0.12	2.91
Water	17,613.64	17,613.64	3.35	83.45
TOTAL (kg/batch)	18,227.69	18,227.69	3.46	86.36
TOTAL (L/batch)	17,911.95	18,073.54	3.43	85.63

Stream Name	Phosphate to SFR-3	Phosphate to FR-1	Salts to SFR-3	Salts to SFR-2
**Source**	**P-2**	**P-2**	**P-65**	**P-64**
**Destination**	**P-65**	**P-4**	**P-15**	**P-1**
Stream Properties				
Activity (U/ml)	0.00	0.00	0.00	0.00
Temperature (°C)	35.00	35.00	35.00	35.00
Pressure (bar)	1.01	1.01	1.01	1.01
Density (g/L)	1,008.53	1,008.53	1,011.95	1,011.95
Total Enthalpy (kW-h)	34.23	684.52	101.88	10.19
Specific Enthalpy (kcal/kg)	34.10	34.10	33.83	33.83
Heat Capacity (kcal/kg-°C)	0.97	0.97	0.96	0.96
Component Flowrates (kg/batch)				
Amm. Sulfate	0.00	0.00	2.71	0.27
Ammonium Chlori	0.00	0.00	107.44	10.74
NaH2PO4	29.10	581.93	29.10	2.91
Water	834.60	16,692.24	2,451.86	245.17
TOTAL (kg/batch)	863.70	17,274.16	2,591.11	259.09
TOTAL (L/batch)	856.40	17,128.08	2,560.50	256.03

Stream Name	S-123	S-125	S-112	S-118
**Source**	**INPUT**	**P-25**	**INPUT**	**P-21**
**Destination**	**P-25**	**P-24**	**P-21**	**P-20**
Stream Properties				
Activity (U/ml)	0.00	0.00	0.00	0.00
Temperature (°C)	25.00	35.00	25.00	35.00
Pressure (bar)	1.01	1.01	1.01	1.01
Density (g/L)	994.70	991.06	994.70	991.06
Total Enthalpy (kW-h)	1,386.27	1,937.96	125.84	175.92
Specific Enthalpy (kcal/kg)	25.11	35.10	25.11	35.10
Heat Capacity (kcal/kg-°C)	1.00	1.00	1.00	1.00
Component Flowrates (kg/batch)				
Water	47,503.93	47,503.93	4,312.27	4,312.27
TOTAL (kg/batch)	47,503.93	47,503.93	4,312.27	4,312.27
TOTAL (L/batch)	47,756.83	47,932.48	4,335.23	4,351.17

Stream Name	S-120	S-122	Water for 50% Sucrose	Process Sucrose
**Source**	**INPUT**	**P-23**	**INPUT**	**INPUT**
**Destination**	**P-23**	**P-22**	**P-9**	**P-9**
Stream Properties				
Activity (U/ml)	0.00	0.00	0.00	0.00
Temperature (°C)	25.00	35.00	25.00	25.00
Pressure (bar)	1.01	1.01	1.01	1.01
Density (g/L)	994.70	991.06	994.70	1,509.84
Total Enthalpy (kW-h)	14.02	19.60	1,303.45	388.65
Specific Enthalpy (kcal/kg)	25.11	35.10	25.11	7.49
Heat Capacity (kcal/kg-°C)	1.00	1.00	1.00	0.30
Component Flowrates (kg/batch)				
Sucrose	0.00	0.00	0.00	44,665.94
Water	480.35	480.35	44,665.94	0.00
TOTAL (kg/batch)	480.35	480.35	44,665.94	44,665.94
TOTAL (L/batch)	482.91	484.68	44,903.73	29,583.29

Stream Name	S-144	S-106	Batch Sucrose	Fed-Batch Sucrose
**Source**	**P-9**	**P-8**	**Sucrose Storage**	**Sucrose Storage**
**Destination**	**P-8**	**Sucrose Storage**	**P-7**	**P-10**
Stream Properties				
Activity (U/ml)	0.00	0.00	0.00	0.00
Temperature (°C)	25.00	35.00	35.00	35.00
Pressure (bar)	1.01	1.01	1.01	1.01
Density (g/L)	1,199.29	1,195.13	1,195.13	1,195.13
Total Enthalpy (kW-h)	1,692.10	2,366.29	193.13	2,173.16
Specific Enthalpy (kcal/kg)	16.30	22.79	22.79	22.79
Heat Capacity (kcal/kg-°C)	0.65	0.65	0.65	0.65
Component Flowrates (kg/batch)				
Sucrose	44,665.94	44,665.94	3,645.54	41,020.39
Water	44,665.94	44,665.94	3,645.54	41,020.39
TOTAL (kg/batch)	89,331.87	89,331.87	7,291.09	82,040.78
TOTAL (L/batch)	74,487.02	74,746.37	6,100.65	68,645.72

Stream Name	Fed-batch Sugar > SFR-1	Fed-Batch Sugar > SFR-2	Fed-Batch Sugar > SFR-3	Fed-Batch Sugar > FR-1
**Source**	**P-10**	**P-10**	**P-10**	**P-10**
**Destination**	**P-16**	**P-1**	**P-15**	**P-4**
Stream Properties				
Activity (U/ml)	0.00	0.00	0.00	0.00
Temperature (°C)	35.00	35.00	35.00	35.00
Pressure (bar)	1.01	1.01	1.01	1.01
Density (g/L)	1,195.13	1,195.13	1,195.13	1,195.13
Total Enthalpy (kW-h)	0.16	1.94	18.50	2,152.56
Specific Enthalpy (kcal/kg)	22.79	22.79	22.79	22.79
Heat Capacity (kcal/kg-°C)	0.65	0.65	0.65	0.65
Component Flowrates (kg/batch)				
Sucrose	3.08	36.55	349.25	40,631.52
Water	3.08	36.55	349.25	40,631.52
TOTAL (kg/batch)	6.15	73.10	698.50	81,263.03
TOTAL (L/batch)	5.15	61.16	584.45	67,994.96

Stream Name	S-110	S-124	S-121	S-127
**Source**	**P-7**	**P-7**	**P-7**	**P-7**
**Destination**	**P-12**	**P-22**	**P-20**	**P-24**
Stream Properties				
Activity (U/ml)	0.00	0.00	0.00	0.00
Temperature (°C)	35.00	35.00	35.00	35.00
Pressure (bar)	1.01	1.01	1.01	1.01
Density (g/L)	1,195.13	1,195.13	1,195.13	1,195.13
Total Enthalpy (kW-h)	0.04	0.92	9.15	183.03
Specific Enthalpy (kcal/kg)	22.79	22.79	22.79	22.79
Heat Capacity (kcal/kg-°C)	0.65	0.65	0.65	0.65
Component Flowrates (kg/batch)				
Sucrose	0.69	17.27	172.74	3,454.84
Water	0.69	17.27	172.74	3,454.84
TOTAL (kg/batch)	1.39	34.55	345.48	6,909.68
TOTAL (L/batch)	1.16	28.90	289.07	5,781.51

Stream Name	Initial Sugar to FR-1	Initial Sugar to SFR-3	Initial Sugar to SFR-2	S-114
**Source**	**P-24**	**P-20**	**P-22**	**INPUT**
**Destination**	**P-4**	**P-15**	**P-1**	**P-18**
Stream Properties				
Activity (U/ml)	0.00	0.00	0.00	0.00
Temperature (°C)	35.00	35.00	35.00	25.00
Pressure (bar)	1.01	1.01	1.01	1.01
Density (g/L)	1,013.02	1,003.77	1,002.54	994.70
Total Enthalpy (kW-h)	2,120.98	185.07	20.51	0.52
Specific Enthalpy (kcal/kg)	33.54	34.19	34.28	25.11
Heat Capacity (kcal/kg-°C)	0.95	0.97	0.98	1.00
Component Flowrates (kg/batch)				
Sucrose	3,454.84	172.74	17.27	0.00
Water	50,958.77	4,485.01	497.62	17.91
TOTAL (kg/batch)	54,413.60	4,657.75	514.89	17.91
TOTAL (L/batch)	53,713.99	4,640.24	513.59	18.01

Stream Name	S-115	Initial Sugar to SFR-1	Air input	S-153
**Source**	**P-18**	**P-12**	**INPUT**	**P-51**
**Destination**	**P-12**	**P-16**	**P-51**	**P-50**
Stream Properties				
Activity (U/ml)	0.00	0.00	0.00	0.00
Temperature (°C)	35.00	35.00	20.00	40.00
Pressure (bar)	1.01	1.01	1.01	6.01
Density (g/L)	991.06	1,003.36	1.20	6.66
Total Enthalpy (kW-h)	0.73	0.77	1,784.43	3,561.75
Specific Enthalpy (kcal/kg)	35.10	34.22	4.85	9.68
Heat Capacity (kcal/kg-°C)	1.00	0.97	0.24	0.24
Component Flowrates (kg/batch)				
Argon	0.00	0.00	2,911.22	2,911.22
Carb. Dioxide	0.00	0.00	126.57	126.57
Nitrogen	0.00	0.00	247,106.04	247,106.04
Oxygen	0.00	0.00	66,293.65	66,293.65
Sucrose	0.00	0.69	0.00	0.00
Water	17.91	18.61	0.00	0.00
TOTAL (kg/batch)	17.91	19.30	316,437.49	316,437.49
TOTAL (L/batch)	18.07	19.23	263,849,277.37	47,492,581.76

Stream Name	S-139	S-148	S-147	S-146
**Source**	**P-50**	**P-41**	**P-41**	**P-41**
**Destination**	**P-41**	**P-16**	**P-1**	**P-15**
Stream Properties				
Activity (U/ml)	0.00	0.00	0.00	0.00
Temperature (°C)	40.00	40.00	40.00	40.00
Pressure (bar)	6.01	6.01	6.01	6.01
Density (g/L)	6.66	6.66	6.66	6.66
Total Enthalpy (kW-h)	3,561.75	0.18	4.13	41.40
Specific Enthalpy (kcal/kg)	9.68	9.68	9.68	9.68
Heat Capacity (kcal/kg-°C)	0.24	0.24	0.24	0.24
Component Flowrates (kg/batch)				
Argon	2,911.22	0.15	3.38	33.84
Carb. Dioxide	126.57	0.01	0.15	1.47
Nitrogen	247,106.04	12.39	286.77	2,872.45
Oxygen	66,293.65	3.32	76.93	770.62
TOTAL (kg/batch)	316,437.49	15.86	367.23	3,678.38
TOTAL (L/batch)	47,492,581.76	2,380.83	55,115.76	552,070.98

Stream Name	S-143	Vent SFR-1	Inoculum to SFR-2	Vent FR-1
**Source**	**P-41**	**P-16**	**P-16**	**P-4**
**Destination**	**P-4**	**OUTPUT**	**P-1**	**P-49**
Stream Properties				
Activity (U/ml)	0.00	0.00	0.00	0.00
Temperature (°C)	40.00	35.00	35.00	34.99
Pressure (bar)	6.01	1.01	1.01	1.01
Density (g/L)	6.66	1.20	993.91	1.17
Total Enthalpy (kW-h)	3,516.03	0.34	1.35	4,794.47
Specific Enthalpy (kcal/kg)	9.68	15.98	35.10	12.25
Heat Capacity (kcal/kg-°C)	0.24	0.24	1.00	0.24
Component Flowrates (kg/batch)				
Amm. Sulfate	0.00	0.00	0.00	0.00
Argon	2,873.86	0.15	0.00	2,877.62
Biomass	0.00	0.00	1.70	0.00
Carb. Dioxide	124.95	2.64	0.00	24,173.35
NaH2PO4	0.00	0.00	0.00	0.00
Nitrogen	243,934.43	12.42	0.00	244,254.06
Oxygen	65,442.77	3.33	0.00	65,528.53
Sucrose	0.00	0.00	0.00	0.00
Water	0.00	0.00	31.51	0.00
TOTAL (kg/batch)	312,376.01	18.53	33.21	336,833.56
TOTAL (L/batch)	46,883,014.20	15,450.06	33.41	287,954,947.13

Stream Name	Emissions	Vent SFR-2	Inoculum to SFR-3	Vent SFR-3
**Source**	**P-49**	**P-1**	**P-1**	**P-15**
**Destination**	**OUTPUT**	**OUTPUT**	**P-15**	**OUTPUT**
Stream Properties				
Activity (U/ml)	0.00	0.00	0.00	0.00
Temperature (°C)	34.99	35.00	35.00	35.00
Pressure (bar)	1.01	1.01	1.01	1.01
Density (g/L)	1.17	1.18	992.80	1.18
Total Enthalpy (kW-h)	4,794.47	6.73	34.13	66.71
Specific Enthalpy (kcal/kg)	12.25	14.06	35.10	13.95
Heat Capacity (kcal/kg-°C)	0.24	0.24	1.00	0.24
Component Flowrates (kg/batch)				
Ammonium Chlori	0.00	0.00	0.01	0.00
Argon	2,877.62	3.39	0.00	33.93
Biomass	0.00	0.00	25.90	0.00
Carb. Dioxide	24,173.35	43.62	0.00	427.56
NaH2PO4	0.00	0.00	0.00	0.00
Nitrogen	244,254.06	287.57	0.00	2,880.40
Oxygen	65,528.53	77.15	0.00	772.75
Sucrose	0.00	0.00	0.03	0.00
Water	0.00	0.00	810.85	0.00
TOTAL (kg/batch)	336,833.56	411.72	836.79	4,114.64
TOTAL (L/batch)	287,954,947.13	347,739.14	842.86	3,477,741.15

Stream Name	Inoculum to FR-1	Mother Liquor	S-116	S-128
Source	P-15	P-11	P-27	P-4
Destination	P-4	P-4	P-4	OUTPUT
Stream Properties				
Activity (U/ml)	0.00	0.00	0.00	0.00
Temperature (°C)	35.00	9.00	35.31	12.55
Pressure (bar)	1.01	1.01	1.01	1.01
Density (g/L)	992.80	1,003.88	1,012.41	1,004.88
Total Enthalpy (kW-h)	340.93	1,672.35	1,021.96	2,695.62
Specific Enthalpy (kcal/kg)	35.10	9.00	34.38	12.50
Heat Capacity (kcal/kg-°C)	1.00	1.00	0.97	0.99
Component Flowrates (kg/batch)				
Amm. Sulfate	0.00	1.25	0.07	1.32
Ammonium Chlori	0.00	49.53	2.92	52.45
Biomass	260.80	0.00	5,087.44	5,087.44
NaH2PO4	0.00	13.41	0.79	14.20
pHBA (aq)	0.00	318.29	938.30	1,256.59
pHBA (solid)	0.00	311.92	0.00	311.92
Sucrose	0.04	1,015.79	59.89	1,075.68
Water	8,096.96	158,212.71	19,490.26	177,702.97
TOTAL (kg/batch)	8,357.80	159,922.90	25,579.67	185,502.57
TOTAL (L/batch)	8,418.41	159,304.72	25,266.00	184,602.01

Stream Name	S-113	S-105	Vent R-101	S-101
**Source**	**P-4**	**P-27**	**P-28**	**P-28**
**Destination**	**P-27**	**P-28**	**OUTPUT**	**P-11**
Stream Properties				
Activity (U/ml)	0.00	0.00	0.00	0.00
Temperature (°C)	35.00	35.31	5.00	5.00
Pressure (bar)	1.01	1.01	1.01	1.01
Density (g/L)	1,020.10	1,021.31	1.26	1,033.71
Total Enthalpy (kW-h)	6,472.29	5,507.60	0.24	784.34
Specific Enthalpy (kcal/kg)	32.41	32.41	1.23	4.62
Heat Capacity (kcal/kg-°C)	0.92	0.91	0.24	0.92
Component Flowrates (kg/batch)				
Amm. Sulfate	1.32	1.25	0.00	1.25
Ammonium Chlori	52.45	49.53	0.00	49.53
Argon	0.00	0.00	1.52	0.00
Biomass	5,087.44	0.00	0.00	0.00
Carb. Dioxide	0.00	0.00	0.07	0.00
NaH2PO4	14.20	13.41	0.00	13.41
Nitrogen	0.00	0.00	129.10	0.00
Oxygen	0.00	0.00	34.63	0.00
pHBA (aq)	16,852.69	15,914.39	0.00	318.29
pHBA (solid)	0.00	0.00	0.00	15,596.10
Sucrose	1,075.68	1,015.79	0.00	1,015.79
Water	148,724.82	129,234.57	0.00	129,234.57
TOTAL (kg/batch)	171,808.60	146,228.93	165.32	146,228.93
TOTAL (L/batch)	168,423.81	143,178.12	130,789.33	141,460.92

Stream Name	Wash Water	S-102	Humid Air	Final Product
Source	INPUT	P-11	P-14	P-14
Destination	P-11	P-14	OUTPUT	OUTPUT
Stream Properties				
Activity (U/ml)	0.00	0.00	0.00	0.00
Temperature (°C)	25.00	22.93	50.00	50.00
Pressure (bar)	1.01	2.84	1.01	1.01
Density (g/L)	994.70	1,211.06	1.08	1,303.70
Total Enthalpy (kW-h)	1,034.75	282.86	9,216.53	243.00
Specific Enthalpy (kcal/kg)	25.11	11.18	24.28	13.61
Heat Capacity (kcal/kg-°C)	1.00	0.49	0.25	0.27
Component Flowrates (kg/batch)				
Amm. Sulfate	0.00	0.00	0.00	0.00
Ammonium Chlori	0.00	0.00	0.00	0.00
Argon	0.00	0.00	2,945.55	0.00
Carb. Dioxide	0.00	0.00	128.07	0.00
NaH2PO4	0.00	0.00	0.00	0.00
Nitrogen	0.00	0.00	250,019.96	0.00
Oxygen	0.00	0.00	67,075.40	0.00
pHBA (aq)	0.00	0.00	0.00	0.00
pHBA (solid)	0.00	15,284.18	0.00	15,284.18
Sucrose	0.00	0.00	0.00	0.00
Water	35,458.33	6,480.18	6,403.38	76.80
TOTAL (kg/batch)	35,458.33	21,764.36	326,572.37	15,360.98
TOTAL (L/batch)	35,647.11	17,971.29	303,705,773.90	11,782.58

4. OVERALL COMPONENT BALANCE (kg/batch)

COMPONENT	INITIAL	INPUT	OUTPUT	FINAL	IN-OUT
Amm. Sulfate	0.00	57.16	1.32	0.00	55.84
Ammonium Chlori	0.00	2,267.50	52.45	0.00	2,215.05
Argon	4.29	5,856.78	5,862.17	2.76	- 3.86
Biomass	0.00	0.00	5,087.44	0.00	- 5,087.44
Carb. Dioxide	0.19	254.64	24,775.30	0.57	- 24,521.03
NaH2PO4	0.00	614.05	14.20	0.00	599.85
Nitrogen	364.19	497,126.00	497,583.50	234.14	- 327.45
Oxygen	97.71	133,369.06	133,491.80	62.82	- 87.85
pHBA (aq)	0.00	0.00	1,256.59	0.00	- 1,256.59
pHBA (solid)	0.00	0.00	15,596.10	0.00	- 15,596.10
Phosphoric Acid	0.00	182.13	182.13	0.00	0.00
Sodium Hydroxid	0.00	245.99	245.99	0.00	0.00
Sucrose	0.00	44,665.94	1,075.68	0.00	43,590.26
Water	0.00	220,625.00	220,625.00	0.00	0.00
TOTAL	466.38	905,264.23	905,849.65	300.29	419.33
				Overall Error:	0,046%

5. EQUIPMENT CONTENTS

SFR-3				
**Procedure**	**Operation**	**Time (in h)**	**Volume (in L)**	**Vapor (in kg)**
P-15	START	25.61	0.00	12.41(*)
P-15	TRANSFER-IN-SALTS (Transfer In)	26.61	2,560.50	12.41(*)
P-15	TRANSFER-IN-INITIAL-SUGAR (Transfer In)	27.61	7,200.74	12.41(*)
P-15	TRANSFER-IN-INOCULUM (Transfer In)	28.11	8,043.60	12.41(*)
P-15	FERMENT-2 (Batch Stoich. Fermentation)	40.11	8,418.41	2.49(*)
P-15	TRANSFER-OUT-1 (Transfer Out)	41.11	0.00	2.49(*)
P-15	CIP-1 (In-Place-Cleaning)	43.19	0.00	2.49(*)
P-15	SIP-1 (In-Place-Steaming)	45.19	0.00	2.49(*)

(*) Contains material in vapor phase other than Oxygen & Nitrogen

SFR-2				
**Procedure**	**Operation**	**Time (in h)**	**Volume (in L)**	**Vapor (in kg)**
P-1	START	14.11	0.00	1.24(*)
P-1	TRANSFER-IN-SALTS (Transfer In)	14.61	256.03	1.24(*)
P-1	TRANSFER-IN-INITIAL-SUGAR (Transfer In)	15.11	769.61	1.24(*)
P-1	TRANSFER-IN-INOCULUM (Transfer In)	15.61	803.03	1.24(*)
P-1	FERMENT-1 (Batch Stoich. Fermentation)	27.61	842.86	0.25(*)
P-1	TRANSFER-OUT-1 (Transfer Out)	28.11	0.00	0.25(*)
P-1	CIP-1 (In-Place-Cleaning)	30.19	0.00	0.25(*)
P-1	SIP-1 (In-Place-Steaming)	31.19	0.00	0.25(*)

(*) Contains material in vapor phase other than Oxygen & Nitrogen

SFR-1				
**Procedure**	**Operation**	**Time (in h)**	**Volume (in L)**	**Vapor (in kg)**
P-16	START	0.00	0.00	0.05(*)
P-16	TRANSFER-IN-PHOSPHATE (Transfer In)	0.25	3.43	0.05(*)
P-16	TRANSFER-IN-SULFATE (Transfer In)	0.50	6.92	0.05(*)
P-16	TRANSFER-IN-NH4Cl (Transfer In)	0.75	10.27	0.05(*)
P-16	TRANSFER-IN-INITIAL-SUGAR (Transfer In)	1.00	29.50	0.05(*)
P-16	FERMENT (Batch Stoich. Fermentation)	15.11	33.41	0.01(*)
P-16	TRANSFER-OUT (Transfer Out)	15.61	0.00	0.01(*)
P-16	CIP-1 (In-Place-Cleaning)	17.69	0.00	0.01(*)
P-16	SIP-1 (In-Place-Steaming)	18.19	0.00	0.01(*)

(*) Contains material in vapor phase other than Oxygen & Nitrogen

FR-1

Procedure	Operation	Time (in h)	Volume (in L)	Vapor (in kg)
P-4	START	39.11	0.00	232.29(*)
P-4	TRANSFER-IN-SULFATE (Transfer In)	40.11	17,405.84	232.29(*)
P-4	TRANSFER-IN-NH4Cl (Transfer In)	40.11	34,082.25	232.29(*)
P-4	TRANSFER-IN-PHOSPHATE (Transfer In)	40.11	51,210.30	232.29(*)
P-4	TRANSFER-IN-INITIAL-SUGAR (Transfer In)	40.11	104,924.28	232.29(*)
P-4	TRANSFER-IN-INOCULUM (Transfer In)	41.11	113,342.79	232.29(*)
P-4	FERMENT-1 (Batch Stoich. Fermentation)	112.43	168,423.81	33.41(*)
P-4	TRANSFER-OUT-2 (Transfer Out)	76.11	0.00	225.57(*)
P-4	TRANSFER-IN-1 (Transfer In)	76.11	25,265.99	196.44(*)
P-4	TRANSFER-IN-2 (Transfer In)	76.11	184,602.01	15.30(*)
P-4	TRANSFER-OUT-1 (Transfer Out)	114.43	0.00	242.46(*)
P-4	CIP-1 (In-Place-Cleaning)	116.51	0.00	242.46(*)
P-4	SIP-1 (In-Place-Steaming)	118.51	0.00	242.46(*)

(*) Contains material in vapor phase other than Oxygen & Nitrogen

R-102

Procedure	Operation	Time (in h)	Volume (in L)	Vapor (in kg)
P-28	START	40.11	0.00	15.63(*)
	AFTER AUTO-INIT	40.11	11,931.51	15.63(*)
P-28	REACT-1 (Batch Stoich. Reaction)	112.11	11,788.41	1.86(*)
P-28	END	112.11	0.00	1.86(*)

(*) Contains material in vapor phase other than Oxygen & Nitrogen

BCFBD-101

Procedure	Operation	Time (in h)	Volume (in L)	Vapor (in kg)
P-11	START	40.11	0.00	1.37(*)
P-11	FILTER-1 (Cloth Filtration)	111.61	742.65	1.37(*)
P-11	CAKE-WASH-1 (Cake Wash)	111.86	748.80	1.37(*)
P-11	TRANSFER-OUT-1 (Transfer Out)	112.11	0.00	1.37(*)

(*) Contains material in vapor phase other than Oxygen & Nitrogen
