# Supplementary material for: Economic Process Evaluation and Environmental Life-Cycle Assessment of Bio-Aromatics Production
Source: Front Bioeng Biotechnol. 2020 May 13;8:403. doi: 10.3389/fbioe.2020.00403 (PMC7237583; doi:10.3389/fbioe.2020.00403)
Supplement: Supplementary file 1 [file Data_Sheet_1.zip › Sc_16.pdf]

Materials & Streams Report

März 21, 2020

for Supplementary_16_yeast_best_case_cane_sugar_upscaled

1. OVERALL PROCESS DATA

Annual Operating Time	7,912.43	h
Unit Production Ref. Rate	50,000,000.00	kg MP/yr
Batch Size	76,687.12	kg MP
Recipe Batch Time	100.43	h
Recipe Cycle Time	12.00	h
Number of Batches per Year	652.00	

MP = Total Flow of Stream 'Final Product'

2.1 STARTING MATERIAL REQUIREMENTS (per Section)

Section	Starting Material	Active Product	Amount Needed (kg Sin/kg MP)	Molar Yield (%)	Mass Yield (%)	Gross Mass Yield (%)
Fermentation Section	(none)	(none)	0.00	Unknown	Unknown	Unknown
Downstream Section	(none)	(none)	0.00	Unknown	Unknown	Unknown

Sin = Section Starting Material, Aout = Section Active Product

2.2 BULK MATERIALS (Entire Process)

Material	kg/yr	kg/batch	kg/kg MP
Air	1,818,036,382	2,788,399.36	36.36
Amm. Sulfate	186,071	285.38	0.00
Ammonium Chlori	7,381,561	11,321.41	0.15
H3PO4 (2%)	13,558,951	20,795.94	0.27
NaH2PO4	1,998,961	3,065.89	0.04
NaOH (0.5 M)	21,098,890	32,360.26	0.42
Sucrose	145,404,501	223,013.04	2.91
Water	624,067,368	957,158.54	12.48
TOTAL	2,631,732,686	4,036,399.83	52.63

2.3 BULK MATERIALS (per Section)

SECTIONS IN: Main Branch

Fermentation Section

Material	kg/yr	kg/batch	kg/kg MP
Air	775,898,134	1,190,027.81	15.52
Amm. Sulfate	186,071	285.38	0.00
Ammonium Chlori	7,381,561	11,321.41	0.15
H3PO4 (2%)	13,558,951	20,795.94	0.27
NaH2PO4	1,998,961	3,065.89	0.04
NaOH (0.5 M)	21,098,890	32,360.26	0.42
Sucrose	145,404,501	223,013.04	2.91
Water	508,650,906	780,139.43	10.17
TOTAL	1,474,177,976	2,261,009.17	29.48

Downstream Section

Material	kg/yr	kg/batch	kg/kg MP
Air	1,042,138,248	1,598,371.55	20.84
Water	115,416,462	177,019.11	2.31
TOTAL	1,157,554,710	1,775,390.66	23.15

2.4 BULK MATERIALS (per Material)

Air

Procedure	% Total	kg/yr	kg/batch	kg/kg MP
Fermentation Section (Main Branch)				
P-51	42.68	775,898,134	1,190,027.81	15.52
Downstream Section (Main Branch)				
P-3	57.32	1,042,138,248	1,598,371.55	20.84
TOTAL	100.00	1,818,036,382	2,788,399.36	36.36

Amm. Sulfate

Procedure	% Total	kg/yr	kg/batch	kg/kg MP
Fermentation Section (Main Branch)				
P-36	100.00	186,071	285.38	0.00
TOTAL	100.00	186,071	285.38	0.00

Ammonium Chlori

Procedure	% Total	kg/yr	kg/batch	kg/kg MP
Fermentation Section (Main Branch)				
P-38	100.00	7,381,561	11,321.41	0.15
TOTAL	100.00	7,381,561	11,321.41	0.15

H3PO4 (2%)

Procedure	% Total	kg/yr	kg/batch	kg/kg MP
Fermentation Section (Main Branch)				
P-4	54.42	7,379,085	11,317.61	0.15
P-1	10.11	1,371,316	2,103.25	0.03
P-15	32.02	4,340,964	6,657.92	0.09
P-16	3.45	467,586	717.16	0.01
TOTAL	100.00	13,558,951	20,795.94	0.27

NaH2PO4

Procedure	% Total	kg/yr	kg/batch	kg/kg MP
Fermentation Section (Main Branch)				
P-34	100.00	1,998,961	3,065.89	0.04
TOTAL	100.00	1,998,961	3,065.89	0.04

NaOH (0.5 M)

Procedure	% Total	kg/yr	kg/batch	kg/kg MP
Fermentation Section (Main Branch)				
P-4	82.31	17,367,438	26,637.18	0.35
P-1	6.56	1,383,229	2,121.52	0.03
P-15	8.89	1,876,575	2,878.18	0.04
P-16	2.24	471,648	723.39	0.01
TOTAL	100.00	21,098,890	32,360.26	0.42

Sucrose

Procedure	% Total	kg/yr	kg/batch	kg/kg MP
Fermentation Section (Main Branch)				
P-9	100.00	145,404,501	223,013.04	2.91
TOTAL	100.00	145,404,501	223,013.04	2.91

Water

Procedure	% Total	kg/yr	kg/batch	kg/kg MP
Fermentation Section (Main Branch)				
P-4	2.61	16,315,852	25,024.31	0.33
P-34	9.19	57,339,056	87,943.34	1.15
P-36	9.48	59,152,171	90,724.19	1.18
P-38	8.33	51,956,456	79,687.82	1.04
P-9	23.30	145,404,501	223,013.04	2.91
P-18	0.01	58,315	89.44	0.00
P-21	2.25	14,038,063	21,530.77	0.28
P-23	0.25	1,563,717	2,398.34	0.03
P-25	24.78	154,643,240	237,182.88	3.09
P-1	0.49	3,032,110	4,650.48	0.06

P-15	0.66	4,113,549	6,309.12	0.08
P-16	0.17	1,033,877	1,585.70	0.02
Downstream Section (Main Branch)				
P-11	18.49	115,416,462	177,019.11	2.31
TOTAL	100.00	624,067,368	957,158.54	12.48

2.5 BULK MATERIALS: SECTION TOTALS (kg/kg MP)

Raw Material	Fermentation Section	Downstream Section
Air	15.52	20.84
Amm. Sulfate	0.00	0.00
Ammonium Chlori	0.15	0.00
H3PO4 (2%)	0.27	0.00
NaH2PO4	0.04	0.00
NaOH (0.5 M)	0.42	0.00
Sucrose	2.91	0.00
Water	10.17	2.31
TOTAL	29.48	23.15

2.6 BULK MATERIALS: SECTION TOTALS (kg/batch)

Raw Material	Fermentation Section	Downstream Section
Air	1,190,027.81	1,598,371.55
Amm. Sulfate	285.38	0.00
Ammonium Chlori	11,321.41	0.00
H3PO4 (2%)	20,795.94	0.00
NaH2PO4	3,065.89	0.00
NaOH (0.5 M)	32,360.26	0.00
Sucrose	223,013.04	0.00
Water	780,139.43	177,019.11
TOTAL	2,261,009.17	1,775,390.66

2.7 BULK MATERIALS: SECTION TOTALS (kg/yr)

Raw Material	Fermentation Section	Downstream Section
Air	775,898,134	1,042,138,248
Amm. Sulfate	186,071	0
Ammonium Chlori	7,381,561	0
H3PO4 (2%)	13,558,951	0
NaH2PO4	1,998,961	0
NaOH (0.5 M)	21,098,890	0
Sucrose	145,404,501	0
Water	508,650,906	115,416,462
TOTAL	1,474,177,976	1,157,554,710

3. STREAM DETAILS

Stream Name	Air for Drying	S-104	Water for NH4Cl	NH4Cl
Source	INPUT	P-3	INPUT	INPUT
Destination	P-3	P-14	P-38	P-38
Stream Properties				
Activity (U/ml)	0.00	0.00	0.00	0.00
Temperature (°C)	25.00	37.66	10.00	20.00
Pressure (bar)	1.01	1.21	1.01	1.01
Density (g/L)	1.18	1.35	1,000.17	1,519.00
Total Enthalpy (kW-h)	11,257.21	16,938.17	933.08	98.89
Specific Enthalpy (kcal/kg)	6.06	9.12	10.07	7.52
Heat Capacity (kcal/kg-°C)	0.24	0.24	1.01	0.38
Component Flowrates (kg/batch)				
Ammonium Chlori	0.00	0.00	0.00	11,321.41
Argon	14,705.02	14,705.02	0.00	0.00
Carb. Dioxide	639.35	639.35	0.00	0.00
Nitrogen	1,248,168.34	1,248,168.34	0.00	0.00
Oxygen	334,858.84	334,858.84	0.00	0.00
Water	0.00	0.00	79,687.82	0.00
TOTAL (kg/batch)	1,598,371.55	1,598,371.55	79,687.82	11,321.41
TOTAL (L/batch)	1,355,472,259.29	1,180,078,435.38	79,674.13	7,453.20

Stream Name	Cl-Solution	S-129	NH4Cl to SFR-1	NH4Cl to SFR-2
Source	P-38	P-37	P-5	P-5
Destination	P-37	P-5	P-16	P-64
Stream Properties				
Activity (U/ml)	0.00	0.00	0.00	0.00
Temperature (°C)	10.50	35.00	35.00	35.00
Pressure (bar)	1.01	1.01	1.01	1.01
Density (g/L)	1,044.38	1,035.84	1,035.84	1,035.84
Total Enthalpy (kW-h)	1,031.97	3,423.97	0.65	16.22
Specific Enthalpy (kcal/kg)	9.76	32.37	32.37	32.37
Heat Capacity (kcal/kg-°C)	0.93	0.92	0.92	0.92
Component Flowrates (kg/batch)				
Ammonium Chlori	11,321.41	11,321.41	2.15	53.64
Water	79,687.82	79,687.82	15.14	377.56
TOTAL (kg/batch)	91,009.23	91,009.23	17.29	431.20
TOTAL (L/batch)	87,141.96	87,859.91	16.69	416.28

Stream Name	NH4Cl to SFR-3	NH4Cl to FR-1	Water for NH4SO4	NH4SO4
**Source**	**P-5**	**P-5**	**INPUT**	**INPUT**
**Destination**	**P-65**	**P-4**	**P-36**	**P-36**
Stream Properties				
Activity (U/ml)	0.00	0.00	0.00	0.00
Temperature (°C)	35.00	35.00	10.00	20.00
Pressure (bar)	1.01	1.01	1.01	1.01
Density (g/L)	1,035.84	1,035.84	1,000.17	1,769.00
Total Enthalpy (kW-h)	162.24	3,244.86	1,062.31	2.25
Specific Enthalpy (kcal/kg)	32.37	32.37	10.07	6.80
Heat Capacity (kcal/kg-°C)	0.92	0.92	1.01	0.34
Component Flowrates (kg/batch)				
Amm. Sulfate	0.00	0.00	0.00	285.38
Ammonium Chlori	536.45	10,729.17	0.00	0.00
Water	3,775.93	75,519.19	90,724.19	0.00
TOTAL (kg/batch)	4,312.38	86,248.35	90,724.19	285.38
TOTAL (L/batch)	4,163.15	83,263.78	90,708.60	161.33

Stream Name	SO4-Solution	S-138	Sulfate to SFR-1	Sulfate to SFR-2
**Source**	**P-36**	**P-35**	**P-6**	**P-6**
**Destination**	**P-35**	**P-6**	**P-16**	**P-64**
Stream Properties				
Activity (U/ml)	0.00	0.00	0.00	0.00
Temperature (°C)	10.01	35.00	35.00	35.00
Pressure (bar)	1.01	1.01	1.01	1.01
Density (g/L)	1,001.53	992.43	992.43	992.43
Total Enthalpy (kW-h)	1,064.56	3,705.10	0.70	17.55
Specific Enthalpy (kcal/kg)	10.06	35.03	35.03	35.03
Heat Capacity (kcal/kg-°C)	1.00	1.00	1.00	1.00
Component Flowrates (kg/batch)				
Amm. Sulfate	285.38	285.38	0.05	1.35
Water	90,724.19	90,724.19	17.24	429.85
TOTAL (kg/batch)	91,009.57	91,009.57	17.29	431.20
TOTAL (L/batch)	90,870.28	91,703.97	17.42	434.49

Stream Name	Sulfate to SFR-3	Sulfate to FR-1	Water for NaH2PO4	NaH2PO4
**Source**	**P-6**	**P-6**	**INPUT**	**INPUT**
**Destination**	**P-65**	**P-4**	**P-34**	**P-34**
Stream Properties				
Activity (U/ml)	0.00	0.00	0.00	0.00
Temperature (°C)	35.00	35.00	10.00	20.00
Pressure (bar)	1.01	1.01	1.01	1.01
Density (g/L)	992.43	992.43	1,000.17	2,040.00
Total Enthalpy (kW-h)	175.56	3,511.28	1,029.75	10.68
Specific Enthalpy (kcal/kg)	35.03	35.03	10.07	3.00
Heat Capacity (kcal/kg-°C)	1.00	1.00	1.01	0.15
Component Flowrates (kg/batch)				
Amm. Sulfate	13.52	270.46	0.00	0.00
NaH2PO4	0.00	0.00	0.00	3,065.89
Water	4,298.87	85,978.22	87,943.34	0.00
TOTAL (kg/batch)	4,312.40	86,248.68	87,943.34	3,065.89
TOTAL (L/batch)	4,345.30	86,906.75	87,928.23	1,502.89

Stream Name	PO4-Solution	S-108	Phosphate to SFR-1	Phosphate to SFR-2
**Source**	**P-34**	**P-33**	**P-2**	**P-2**
**Destination**	**P-33**	**P-2**	**P-16**	**P-64**
Stream Properties				
Activity (U/ml)	0.00	0.00	0.00	0.00
Temperature (°C)	10.05	35.00	35.00	35.00
Pressure (bar)	1.01	1.01	1.01	1.01
Density (g/L)	1,017.63	1,008.53	1,008.53	1,008.53
Total Enthalpy (kW-h)	1,040.43	3,606.40	0.69	17.09
Specific Enthalpy (kcal/kg)	9.84	34.10	34.10	34.10
Heat Capacity (kcal/kg-°C)	0.98	0.97	0.97	0.97
Component Flowrates (kg/batch)				
NaH2PO4	3,065.89	3,065.89	0.58	14.53
Water	87,943.34	87,943.34	16.71	416.68
TOTAL (kg/batch)	91,009.23	91,009.23	17.29	431.20
TOTAL (L/batch)	89,432.76	90,239.59	17.15	427.56

Stream Name	Phosphate to SFR-3	Phosphate to FR-1	Salts to SFR-3	Salts to SFR-2
**Source**	**P-2**	**P-2**	**P-65**	**P-64**
**Destination**	**P-65**	**P-4**	**P-15**	**P-1**
Stream Properties				
Activity (U/ml)	0.00	0.00	0.00	0.00
Temperature (°C)	35.00	35.00	35.00	35.00
Pressure (bar)	1.01	1.01	1.01	1.01
Density (g/L)	1,008.53	1,008.53	1,011.95	1,011.95
Total Enthalpy (kW-h)	170.89	3,417.75	508.69	50.86
Specific Enthalpy (kcal/kg)	34.10	34.10	33.83	33.83
Heat Capacity (kcal/kg-°C)	0.97	0.97	0.96	0.96
Component Flowrates (kg/batch)				
Amm. Sulfate	0.00	0.00	13.52	1.35
Ammonium Chlori	0.00	0.00	536.45	53.64
NaH2PO4	145.27	2,905.51	145.27	14.53
Water	4,167.11	83,342.84	12,241.91	1,224.09
TOTAL (kg/batch)	4,312.38	86,248.35	12,937.16	1,293.61
TOTAL (L/batch)	4,275.91	85,518.98	12,784.37	1,278.33

Stream Name	S-123	S-125	S-112	S-118
**Source**	**INPUT**	**P-25**	**INPUT**	**P-21**
**Destination**	**P-25**	**P-24**	**P-21**	**P-20**
Stream Properties				
Activity (U/ml)	0.00	0.00	0.00	0.00
Temperature (°C)	25.00	35.00	25.00	35.00
Pressure (bar)	1.01	1.01	1.01	1.01
Density (g/L)	994.70	991.06	994.70	991.06
Total Enthalpy (kW-h)	6,921.50	9,676.04	628.31	878.36
Specific Enthalpy (kcal/kg)	25.11	35.10	25.11	35.10
Heat Capacity (kcal/kg-°C)	1.00	1.00	1.00	1.00
Component Flowrates (kg/batch)				
Water	237,182.88	237,182.88	21,530.77	21,530.77
TOTAL (kg/batch)	237,182.88	237,182.88	21,530.77	21,530.77
TOTAL (L/batch)	238,445.61	239,322.59	21,645.40	21,725.01

Stream Name	S-120	S-122	Water for 50% Sucrose	Process Sucrose
**Source**	**INPUT**	**P-23**	**INPUT**	**INPUT**
**Destination**	**P-23**	**P-22**	**P-9**	**P-9**
Stream Properties				
Activity (U/ml)	0.00	0.00	0.00	0.00
Temperature (°C)	25.00	35.00	25.00	25.00
Pressure (bar)	1.01	1.01	1.01	1.01
Density (g/L)	994.70	991.06	994.70	1,509.84
Total Enthalpy (kW-h)	69.99	97.84	6,507.99	1,940.50
Specific Enthalpy (kcal/kg)	25.11	35.10	25.11	7.49
Heat Capacity (kcal/kg-°C)	1.00	1.00	1.00	0.30
Component Flowrates (kg/batch)				
Sucrose	0.00	0.00	0.00	223,013.04
Water	2,398.34	2,398.34	223,013.04	0.00
TOTAL (kg/batch)	2,398.34	2,398.34	223,013.04	223,013.04
TOTAL (L/batch)	2,411.11	2,419.98	224,200.33	147,706.73

Stream Name	S-144	S-106	Batch Sucrose	Fed-Batch Sucrose
**Source**	**P-9**	**P-8**	**Sucrose Storage**	**Sucrose Storage**
**Destination**	**P-8**	**Sucrose Storage**	**P-7**	**P-10**
Stream Properties				
Activity (U/ml)	0.00	0.00	0.00	0.00
Temperature (°C)	25.00	35.00	35.00	35.00
Pressure (bar)	1.01	1.01	1.01	1.01
Density (g/L)	1,199.29	1,195.13	1,195.13	1,195.13
Total Enthalpy (kW-h)	8,448.49	11,814.67	964.29	10,850.38
Specific Enthalpy (kcal/kg)	16.30	22.79	22.79	22.79
Heat Capacity (kcal/kg-°C)	0.65	0.65	0.65	0.65
Component Flowrates (kg/batch)				
Sucrose	223,013.04	223,013.04	18,201.88	204,811.16
Water	223,013.04	223,013.04	18,201.88	204,811.16
TOTAL (kg/batch)	446,026.08	446,026.08	36,403.76	409,622.32
TOTAL (L/batch)	371,907.06	373,201.98	30,460.00	342,741.98

Stream Name	Fed-batch Sugar > SFR-1	Fed-Batch Sugar > SFR-2	Fed-Batch Sugar > SFR-3	Fed-Batch Sugar > FR-1
**Source**	**P-10**	**P-10**	**P-10**	**P-10**
**Destination**	**P-16**	**P-1**	**P-15**	**P-4**
Stream Properties				
Activity (U/ml)	0.00	0.00	0.00	0.00
Temperature (°C)	35.00	35.00	35.00	35.00
Pressure (bar)	1.01	1.01	1.01	1.01
Density (g/L)	1,195.13	1,195.13	1,195.13	1,195.13
Total Enthalpy (kW-h)	0.81	9.67	92.38	10,747.52
Specific Enthalpy (kcal/kg)	22.79	22.79	22.79	22.79
Heat Capacity (kcal/kg-°C)	0.65	0.65	0.65	0.65
Component Flowrates (kg/batch)				
Sucrose	15.36	182.49	1,743.76	202,869.55
Water	15.36	182.49	1,743.76	202,869.55
TOTAL (kg/batch)	30.72	364.97	3,487.52	405,739.10
TOTAL (L/batch)	25.71	305.38	2,918.11	339,492.79

Stream Name	S-110	S-124	S-121	S-127
**Source**	**P-7**	**P-7**	**P-7**	**P-7**
**Destination**	**P-12**	**P-22**	**P-20**	**P-24**
Stream Properties				
Activity (U/ml)	0.00	0.00	0.00	0.00
Temperature (°C)	35.00	35.00	35.00	35.00
Pressure (bar)	1.01	1.01	1.01	1.01
Density (g/L)	1,195.13	1,195.13	1,195.13	1,195.13
Total Enthalpy (kW-h)	0.18	4.57	45.69	913.85
Specific Enthalpy (kcal/kg)	22.79	22.79	22.79	22.79
Heat Capacity (kcal/kg-°C)	0.65	0.65	0.65	0.65
Component Flowrates (kg/batch)				
Sucrose	3.46	86.24	862.48	17,249.70
Water	3.46	86.24	862.48	17,249.70
TOTAL (kg/batch)	6.92	172.48	1,724.96	34,499.40
TOTAL (L/batch)	5.79	144.32	1,443.32	28,866.58

Stream Name	Initial Sugar to FR-1	Initial Sugar to SFR-3	Initial Sugar to SFR-2	S-114
**Source**	**P-24**	**P-20**	**P-22**	**INPUT**
**Destination**	**P-4**	**P-15**	**P-1**	**P-18**
Stream Properties				
Activity (U/ml)	0.00	0.00	0.00	0.00
Temperature (°C)	35.00	35.00	35.00	25.00
Pressure (bar)	1.01	1.01	1.01	1.01
Density (g/L)	1,013.02	1,003.77	1,002.54	994.70
Total Enthalpy (kW-h)	10,589.89	924.05	102.41	2.61
Specific Enthalpy (kcal/kg)	33.54	34.19	34.28	25.11
Heat Capacity (kcal/kg-°C)	0.95	0.97	0.98	1.00
Component Flowrates (kg/batch)				
Sucrose	17,249.70	862.48	86.24	0.00
Water	254,432.58	22,393.25	2,484.58	89.44
TOTAL (kg/batch)	271,682.29	23,255.73	2,570.82	89.44
TOTAL (L/batch)	268,189.16	23,168.32	2,564.30	89.92

Stream Name	S-115	Initial Sugar to SFR-1	Air input	S-153
**Source**	**P-18**	**P-12**	**INPUT**	**P-51**
**Destination**	**P-12**	**P-16**	**P-51**	**P-50**
Stream Properties				
Activity (U/ml)	0.00	0.00	0.00	0.00
Temperature (°C)	35.00	35.00	20.00	40.00
Pressure (bar)	1.01	1.01	1.01	6.01
Density (g/L)	991.06	1,003.36	1.20	6.66
Total Enthalpy (kW-h)	3.65	3.83	6,710.70	13,394.67
Specific Enthalpy (kcal/kg)	35.10	34.22	4.85	9.68
Heat Capacity (kcal/kg-°C)	1.00	0.97	0.24	0.24
Component Flowrates (kg/batch)				
Argon	0.00	0.00	10,948.26	10,948.26
Carb. Dioxide	0.00	0.00	476.01	476.01
Nitrogen	0.00	0.00	929,292.72	929,292.72
Oxygen	0.00	0.00	249,310.83	249,310.83
Sucrose	0.00	3.46	0.00	0.00
Water	89.44	92.90	0.00	0.00
TOTAL (kg/batch)	89.44	96.36	1,190,027.81	1,190,027.81
TOTAL (L/batch)	90.25	96.03	992,259,100.83	178,605,554.44

Stream Name	S-139	S-148	S-147	S-146
Source	P-50	P-41	P-41	P-41
Destination	P-41	P-16	P-1	P-15
Stream Properties				
Activity (U/ml)	0.00	0.00	0.00	0.00
Temperature (°C)	40.00	40.00	40.00	40.00
Pressure (bar)	6.01	6.01	6.01	6.01
Density (g/L)	6.66	6.66	6.66	6.66
Total Enthalpy (kW-h)	13,394.67	0.89	20.64	206.72
Specific Enthalpy (kcal/kg)	9.68	9.68	9.68	9.68
Heat Capacity (kcal/kg-°C)	0.24	0.24	0.24	0.24
Component Flowrates (kg/batch)				
Argon	10,948.26	0.73	16.87	168.97
Carb. Dioxide	476.01	0.03	0.73	7.35
Nitrogen	929,292.72	61.85	1,431.82	14,341.89
Oxygen	249,310.83	16.59	384.13	3,847.64
TOTAL (kg/batch)	1,190,027.81	79.20	1,833.55	18,365.84
TOTAL (L/batch)	178,605,554.44	11,887.26	275,188.06	2,756,441.27

Stream Name	S-143	Vent SFR-1	Inoculum to SFR-2	Vent FR-1
Source	P-41	P-16	P-16	P-4
Destination	P-4	OUTPUT	P-1	P-49
Stream Properties				
Activity (U/ml)	0.00	0.00	0.00	0.00
Temperature (°C)	40.00	35.00	35.00	34.99
Pressure (bar)	6.01	1.01	1.01	1.01
Density (g/L)	6.66	1.20	993.91	1.18
Total Enthalpy (kW-h)	13,166.42	1.72	6.76	20,096.28
Specific Enthalpy (kcal/kg)	9.68	15.98	35.10	13.38
Heat Capacity (kcal/kg-°C)	0.24	0.24	1.00	0.24
Component Flowrates (kg/batch)				
Amm. Sulfate	0.00	0.00	0.00	0.00
Argon	10,761.69	0.73	0.00	10,780.50
Biomass	0.00	0.00	8.47	0.00
Carb. Dioxide	467.90	13.16	0.00	120,526.63
NaH2PO4	0.00	0.00	0.00	0.00
Nitrogen	913,457.17	62.01	0.00	915,053.19
Oxygen	245,062.46	16.64	0.00	245,490.64
Sucrose	0.00	0.00	0.00	0.00
Water	0.00	0.00	157.35	0.00
TOTAL (kg/batch)	1,169,749.22	92.53	165.82	1,291,850.97
TOTAL (L/batch)	175,562,037.85	77,140.79	166.83	1,095,986,560.73

Stream Name	Emissions	Vent SFR-2	Inoculum to SFR-3	Vent SFR-3
**Source**	**P-49**	**P-1**	**P-1**	**P-15**
**Destination**	**OUTPUT**	**OUTPUT**	**P-15**	**OUTPUT**
Stream Properties				
Activity (U/ml)	0.00	0.00	0.00	0.00
Temperature (°C)	34.99	35.00	35.00	35.00
Pressure (bar)	1.01	1.01	1.01	1.01
Density (g/L)	1.18	1.18	992.80	1.18
Total Enthalpy (kW-h)	20,096.28	33.59	170.42	333.08
Specific Enthalpy (kcal/kg)	13.38	14.06	35.10	13.95
Heat Capacity (kcal/kg-°C)	0.24	0.24	1.00	0.24
Component Flowrates (kg/batch)				
Ammonium Chlori	0.00	0.00	0.03	0.00
Argon	10,780.50	16.92	0.00	169.43
Biomass	0.00	0.00	129.32	0.00
Carb. Dioxide	120,526.63	217.79	0.00	2,134.76
NaH2PO4	0.00	0.00	0.01	0.00
Nitrogen	915,053.19	1,435.79	0.00	14,381.57
Oxygen	245,490.64	385.19	0.00	3,858.29
Sucrose	0.00	0.00	0.17	0.00
Water	0.00	0.00	4,048.50	0.00
TOTAL (kg/batch)	1,291,850.97	2,055.69	4,178.03	20,544.05
TOTAL (L/batch)	1,095,986,560.73	1,736,230.59	4,208.32	17,364,052.05

Stream Name	Inoculum to FR-1	Mother Liquor	S-116	S-128
Source	P-15	P-11	P-27	P-4
Destination	P-4	P-4	P-4	OUTPUT
Stream Properties				
Activity (U/ml)	0.00	0.00	0.00	0.00
Temperature (°C)	35.00	9.00	35.31	12.55
Pressure (bar)	1.01	1.01	1.01	1.01
Density (g/L)	992.80	1,003.89	1,012.42	1,004.89
Total Enthalpy (kW-h)	1,702.21	8,350.29	5,102.56	13,458.72
Specific Enthalpy (kcal/kg)	35.10	9.00	34.38	12.50
Heat Capacity (kcal/kg-°C)	1.00	1.00	0.97	0.99
Component Flowrates (kg/batch)				
Amm. Sulfate	0.00	6.26	0.37	6.63
Ammonium Chlori	0.02	248.32	14.64	262.97
Biomass	1,302.13	0.00	25,398.62	25,398.62
NaH2PO4	0.00	67.23	3.96	71.19
pHBA (aq)	0.00	1,589.00	4,685.30	6,274.30
pHBA (solid)	0.00	1,557.22	0.00	1,557.22
Sucrose	0.18	5,092.81	300.33	5,393.14
Water	40,427.42	789,922.82	97,315.24	887,238.06
TOTAL (kg/batch)	41,729.76	798,483.66	127,718.46	926,202.12
TOTAL (L/batch)	42,032.39	795,389.51	126,151.88	921,697.60

Stream Name	S-113	S-105	Vent R-101	S-101
**Source**	**P-4**	**P-27**	**P-28**	**P-28**
**Destination**	**P-27**	**P-28**	**OUTPUT**	**P-11**
Stream Properties				
Activity (U/ml)	0.00	0.00	0.00	0.00
Temperature (°C)	35.00	35.31	5.00	5.00
Pressure (bar)	1.01	1.01	1.01	1.01
Density (g/L)	1,020.10	1,021.32	1.26	1,033.71
Total Enthalpy (kW-h)	32,315.67	27,499.03	1.18	3,916.15
Specific Enthalpy (kcal/kg)	32.41	32.41	1.23	4.62
Heat Capacity (kcal/kg-°C)	0.92	0.91	0.24	0.92
Component Flowrates (kg/batch)				
Amm. Sulfate	6.63	6.26	0.00	6.26
Ammonium Chlori	262.97	248.32	0.00	248.32
Argon	0.00	0.00	7.59	0.00
Biomass	25,398.62	0.00	0.00	0.00
Carb. Dioxide	0.00	0.00	0.33	0.00
NaH2PO4	71.19	67.23	0.00	67.23
Nitrogen	0.00	0.00	644.57	0.00
Oxygen	0.00	0.00	172.92	0.00
pHBA (aq)	84,135.20	79,449.89	0.00	1,589.00
pHBA (solid)	0.00	0.00	0.00	77,860.90
Sucrose	5,393.14	5,092.81	0.00	5,092.81
Water	742,569.81	645,254.58	0.00	645,254.58
TOTAL (kg/batch)	857,837.55	730,119.09	825.42	730,119.09
TOTAL (L/batch)	840,931.84	714,881.32	653,024.81	706,307.56

Stream Name	Wash Water	S-102	Humid Air	Final Product
**Source**	**INPUT**	**P-11**	**P-14**	**P-14**
**Destination**	**P-11**	**P-14**	**OUTPUT**	**OUTPUT**
Stream Properties				
Activity (U/ml)	0.00	0.00	0.00	0.00
Temperature (°C)	25.00	22.93	50.00	50.00
Pressure (bar)	1.01	1.78	1.01	1.01
Density (g/L)	994.70	1,211.06	1.08	1,303.70
Total Enthalpy (kW-h)	5,165.79	1,412.13	46,011.45	1,213.13
Specific Enthalpy (kcal/kg)	25.11	11.18	24.28	13.61
Heat Capacity (kcal/kg-°C)	1.00	0.49	0.25	0.27
Component Flowrates (kg/batch)				
Amm. Sulfate	0.00	0.00	0.00	0.00
Ammonium Chlori	0.00	0.00	0.00	0.00
Argon	0.00	0.00	14,705.02	0.00
Carb. Dioxide	0.00	0.00	639.35	0.00
NaH2PO4	0.00	0.00	0.00	0.00
Nitrogen	0.00	0.00	1,248,168.34	0.00
Oxygen	0.00	0.00	334,858.84	0.00
pHBA (aq)	0.00	0.00	0.00	0.00
pHBA (solid)	0.00	76,303.68	0.00	76,303.68
Sucrose	0.00	0.00	0.00	0.00
Water	177,019.11	32,350.87	31,967.43	383.44
TOTAL (kg/batch)	177,019.11	108,654.55	1,630,338.98	76,687.12
TOTAL (L/batch)	177,961.54	89,718.27	1,516,182,664.91	58,822.55

4. OVERALL COMPONENT BALANCE (kg/batch)

COMPONENT	INITIAL	INPUT	OUTPUT	FINAL	IN-OUT
Amm. Sulfate	0.00	285.38	6.63	0.00	278.75
Ammonium Chlori	0.00	11,321.41	262.97	0.00	11,058.45
Argon	22.16	25,653.27	25,680.19	14.51	- 19.26
Biomass	0.00	0.00	25,398.62	0.00	- 25,398.62
Carb. Dioxide	0.96	1,115.36	123,532.03	3.12	- 122,418.82
NaH2PO4	0.00	3,065.89	71.19	0.00	2,994.70
Nitrogen	1,881.02	2,177,461.06	2,179,745.46	1,231.55	- 1,634.94
Oxygen	504.64	584,169.67	584,782.53	330.40	- 438.62
pHBA (aq)	0.00	0.00	6,274.30	0.00	- 6,274.30
pHBA (solid)	0.00	0.00	77,860.90	0.00	- 77,860.90
Phosphoric Acid	0.00	415.92	415.92	0.00	0.00
Sodium Hydroxid	0.00	634.26	634.26	0.00	0.00
Sucrose	0.00	223,013.04	5,393.14	0.00	217,619.90
Water	0.00	1,009,264.56	1,009,264.56	0.00	- 0.00
TOTAL	2,408.78	4,036,399.83	4,039,322.69	1,579.58	2,093.66
				Overall Error:	0,052%

5. EQUIPMENT CONTENTS

SFR-3

Procedure	Operation	Time (in h)	Volume (in L)	Vapor (in kg)
P-15	START	25.61	0.00	61.96(*)
P-15	TRANSFER-IN-SALTS (Transfer In)	26.61	12,784.32	61.96(*)
P-15	TRANSFER-IN-INITIAL-SUGAR (Transfer In)	27.61	35,952.63	61.96(*)
P-15	TRANSFER-IN-INOCULUM (Transfer In)	28.11	40,160.98	61.96(*)
P-15	FERMENT-2 (Batch Stoich. Fermentation)	40.11	42,032.39	12.43(*)
P-15	TRANSFER-OUT-1 (Transfer Out)	41.11	0.00	12.43(*)
P-15	CIP-1 (In-Place-Cleaning)	43.19	0.00	12.43(*)
P-15	SIP-1 (In-Place-Steaming)	45.19	0.00	12.43(*)

(*) Contains material in vapor phase other than Oxygen & Nitrogen

SFR-2

Procedure	Operation	Time (in h)	Volume (in L)	Vapor (in kg)
P-1	START	14.11	0.00	6.20(*)
P-1	TRANSFER-IN-SALTS (Transfer In)	14.61	1,278.32	6.20(*)
P-1	TRANSFER-IN-INITIAL-SUGAR (Transfer In)	15.11	3,842.62	6.20(*)
P-1	TRANSFER-IN-INOCULUM (Transfer In)	15.61	4,009.45	6.20(*)
P-1	FERMENT-1 (Batch Stoich. Fermentation)	27.61	4,208.32	1.25(*)
P-1	TRANSFER-OUT-1 (Transfer Out)	28.11	0.00	1.25(*)
P-1	CIP-1 (In-Place-Cleaning)	30.19	0.00	1.25(*)
P-1	SIP-1 (In-Place-Steaming)	31.19	0.00	1.25(*)

(*) Contains material in vapor phase other than Oxygen & Nitrogen

SFR-1

Procedure	Operation	Time (in h)	Volume (in L)	Vapor (in kg)
P-16	START	0.00	0.00	0.25(*)
P-16	TRANSFER-IN-PHOSPHATE (Transfer In)	0.25	17.15	0.25(*)
P-16	TRANSFER-IN-SULFATE (Transfer In)	0.50	34.57	0.25(*)
P-16	TRANSFER-IN-NH4Cl (Transfer In)	0.75	51.26	0.25(*)
P-16	TRANSFER-IN-INITIAL-SUGAR (Transfer In)	1.00	147.30	0.25(*)
P-16	FERMENT (Batch Stoich. Fermentation)	15.11	166.83	0.05(*)
P-16	TRANSFER-OUT (Transfer Out)	15.61	0.00	0.05(*)
P-16	CIP-1 (In-Place-Cleaning)	17.69	0.00	0.05(*)
P-16	SIP-1 (In-Place-Steaming)	18.19	0.00	0.05(*)

(*) Contains material in vapor phase other than Oxygen & Nitrogen

**FR-1**				
**Procedure**	**Operation**	**Time (in h)**	**Volume (in L)**	**Vapor (in kg)**
P-4	START	39.11	0.00	1,159.80(*)
P-4	TRANSFER-IN-SULFATE (Transfer In)	40.11	86,905.82	1,159.80(*)
P-4	TRANSFER-IN-NH4Cl (Transfer In)	40.11	170,169.66	1,159.80(*)
P-4	TRANSFER-IN-PHOSPHATE (Transfer In)	40.11	255,688.45	1,159.80(*)
P-4	TRANSFER-IN-INITIAL-SUGAR (Transfer In)	40.11	523,877.57	1,159.80(*)
P-4	TRANSFER-IN-INOCULUM (Transfer In)	41.11	565,910.47	1,159.80(*)
P-4	FERMENT-1 (Batch Stoich. Fermentation)	94.35	840,931.84	168.10(*)
P-4	TRANSFER-OUT-2 (Transfer Out)	76.11	0.00	1,127.54(*)
P-4	TRANSFER-IN-1 (Transfer In)	76.11	126,151.87	981.93(*)
P-4	TRANSFER-IN-2 (Transfer In)	76.11	921,697.60	76.47(*)
P-4	TRANSFER-OUT-1 (Transfer Out)	96.35	0.00	1,210.69(*)
P-4	CIP-1 (In-Place-Cleaning)	98.43	0.00	1,210.69(*)
P-4	SIP-1 (In-Place-Steaming)	100.43	0.00	1,210.69(*)

(*) Contains material in vapor phase other than Oxygen & Nitrogen

**R-102**				
**Procedure**	**Operation**	**Time (in h)**	**Volume (in L)**	**Vapor (in kg)**
P-28	START	40.11	0.00	104.07(*)
	AFTER AUTO-INIT	40.11	79,431.26	104.07(*)
P-28	REACT-1 (Batch Stoich. Reaction)	94.11	78,478.62	12.36(*)
P-28	END	94.11	0.00	12.36(*)

(*) Contains material in vapor phase other than Oxygen & Nitrogen

**BCFBD-101**				
**Procedure**	**Operation**	**Time (in h)**	**Volume (in L)**	**Vapor (in kg)**
P-11	START	40.11	0.00	13.55(*)
P-11	FILTER-1 (Cloth Filtration)	93.61	4,943.38	13.55(*)
P-11	CAKE-WASH-1 (Cake Wash)	93.86	4,984.35	13.55(*)
P-11	TRANSFER-OUT-1 (Transfer Out)	94.11	0.00	13.55(*)

(*) Contains material in vapor phase other than Oxygen & Nitrogen
