# Supplementary material for: Economic Process Evaluation and Environmental Life-Cycle Assessment of Bio-Aromatics Production
Source: Front Bioeng Biotechnol. 2020 May 13;8:403. doi: 10.3389/fbioe.2020.00403 (PMC7237583; doi:10.3389/fbioe.2020.00403)
Supplement: Supplementary file 1 [file Data_Sheet_1.zip › Sc_17.pdf]

Materials & Streams Report

März 21, 2020

for Supplementary_17_yeast_best_case_cane_sugar_upscaled

1. OVERALL PROCESS DATA

Annual Operating Time	7,918.51	h
Unit Production Ref. Rate	50,000,000.00	kg MP/yr
Batch Size	76,804.92	kg MP
Recipe Batch Time	118.51	h
Recipe Cycle Time	12.00	h
Number of Batches per Year	651.00	

MP = Total Flow of Stream 'Final Product'

2.1 STARTING MATERIAL REQUIREMENTS (per Section)

Section	Starting Material	Active Product	Amount Needed (kg Sin/kg MP)	Molar Yield (%)	Mass Yield (%)	Gross Mass Yield (%)
Fermentation Section	(none)	(none)	0.00	Unknown	Unknown	Unknown
Downstream Section	(none)	(none)	0.00	Unknown	Unknown	Unknown

Sin = Section Starting Material, Aout = Section Active Product

2.2 BULK MATERIALS (Entire Process)

Material	kg/yr	kg/batch	kg/kg MP
Air	2,072,154,314	3,183,032.74	41.44
Amm. Sulfate	186,049	285.79	0.00
Ammonium Chlori	7,380,704	11,337.49	0.15
H3PO4 (2%)	13,544,540	20,805.74	0.27
NaH2PO4	1,998,729	3,070.24	0.04
NaOH (0.5 M)	21,076,449	32,375.50	0.42
Sucrose	145,387,619	223,329.68	2.91
Water	623,985,560	958,503.16	12.48
TOTAL	2,885,713,964	4,432,740.34	57.71

2.3 BULK MATERIALS (per Section)

SECTIONS IN: Main Branch

Fermentation Section

Material	kg/yr	kg/batch	kg/kg MP
Air	1,030,004,028	1,582,187.45	20.60
Amm. Sulfate	186,049	285.79	0.00
Ammonium Chlori	7,380,704	11,337.49	0.15
H3PO4 (2%)	13,544,540	20,805.74	0.27
NaH2PO4	1,998,729	3,070.24	0.04
NaOH (0.5 M)	21,076,449	32,375.50	0.42
Sucrose	145,387,619	223,329.68	2.91
Water	508,568,649	781,211.44	10.17
TOTAL	1,728,146,767	2,654,603.33	34.56

Downstream Section

Material	kg/yr	kg/batch	kg/kg MP
Air	1,042,150,286	1,600,845.29	20.84
Water	115,416,911	177,291.72	2.31
TOTAL	1,157,567,197	1,778,137.01	23.15

2.4 BULK MATERIALS (per Material)

Air

Procedure	% Total	kg/yr	kg/batch	kg/kg MP
Fermentation Section (Main Branch)				
P-51	49.71	1,030,004,028	1,582,187.45	20.60
Downstream Section (Main Branch)				
P-3	50.29	1,042,150,286	1,600,845.29	20.84
TOTAL	100.00	2,072,154,314	3,183,032.74	41.44

Amm. Sulfate

Procedure	% Total	kg/yr	kg/batch	kg/kg MP
Fermentation Section (Main Branch)				
P-36	100.00	186,049	285.79	0.00
TOTAL	100.00	186,049	285.79	0.00

Ammonium Chlori

Procedure	% Total	kg/yr	kg/batch	kg/kg MP
Fermentation Section (Main Branch)				
P-38	100.00	7,380,704	11,337.49	0.15
TOTAL	100.00	7,380,704	11,337.49	0.15

H3PO4 (2%)

Procedure	% Total	kg/yr	kg/batch	kg/kg MP
Fermentation Section (Main Branch)				
P-4	54.42	7,371,233	11,322.94	0.15
P-1	10.11	1,369,861	2,104.24	0.03
P-15	32.02	4,336,356	6,661.07	0.09
P-16	3.45	467,090	717.50	0.01
TOTAL	100.00	13,544,540	20,805.74	0.27

NaH2PO4

Procedure	% Total	kg/yr	kg/batch	kg/kg MP
Fermentation Section (Main Branch)				
P-34	100.00	1,998,729	3,070.24	0.04
TOTAL	100.00	1,998,729	3,070.24	0.04

NaOH (0.5 M)

Procedure	% Total	kg/yr	kg/batch	kg/kg MP
Fermentation Section (Main Branch)				
P-4	82.31	17,348,958	26,649.70	0.35
P-1	6.56	1,381,761	2,122.52	0.03
P-15	8.89	1,874,583	2,879.54	0.04
P-16	2.24	471,148	723.73	0.01
TOTAL	100.00	21,076,449	32,375.50	0.42

Sucrose

Procedure	% Total	kg/yr	kg/batch	kg/kg MP
Fermentation Section (Main Branch)				
P-9	100.00	145,387,619	223,329.68	2.91
TOTAL	100.00	145,387,619	223,329.68	2.91

Water

Procedure	% Total	kg/yr	kg/batch	kg/kg MP
Fermentation Section (Main Branch)				
P-4	2.61	16,298,491	25,036.08	0.33
P-34	9.19	57,332,398	88,068.20	1.15
P-36	9.48	59,145,303	90,853.00	1.18
P-38	8.33	51,950,423	79,800.96	1.04
P-9	23.30	145,387,619	223,329.68	2.91
P-18	0.01	58,308	89.57	0.00
P-21	2.25	14,036,434	21,561.34	0.28
P-23	0.25	1,563,536	2,401.74	0.03
P-25	24.78	154,625,285	237,519.64	3.09
P-1	0.49	3,028,891	4,652.68	0.06

P-15	0.66	4,109,183	6,312.11	0.08
P-16	0.17	1,032,780	1,586.45	0.02
Downstream Section (Main Branch)				
P-11	18.50	115,416,911	177,291.72	2.31
TOTAL	100.00	623,985,560	958,503.16	12.48

2.5 BULK MATERIALS: SECTION TOTALS (kg/kg MP)

Raw Material	Fermentation Section	Downstream Section
Air	20.60	20.84
Amm. Sulfate	0.00	0.00
Ammonium Chlori	0.15	0.00
H3PO4 (2%)	0.27	0.00
NaH2PO4	0.04	0.00
NaOH (0.5 M)	0.42	0.00
Sucrose	2.91	0.00
Water	10.17	2.31
TOTAL	34.56	23.15

2.6 BULK MATERIALS: SECTION TOTALS (kg/batch)

Raw Material	Fermentation Section	Downstream Section
Air	1,582,187.45	1,600,845.29
Amm. Sulfate	285.79	0.00
Ammonium Chlori	11,337.49	0.00
H3PO4 (2%)	20,805.74	0.00
NaH2PO4	3,070.24	0.00
NaOH (0.5 M)	32,375.50	0.00
Sucrose	223,329.68	0.00
Water	781,211.44	177,291.72
TOTAL	2,654,603.33	1,778,137.01

2.7 BULK MATERIALS: SECTION TOTALS (kg/yr)

Raw Material	Fermentation Section	Downstream Section
Air	1,030,004,028	1,042,150,286
Amm. Sulfate	186,049	0
Ammonium Chlori	7,380,704	0
H3PO4 (2%)	13,544,540	0
NaH2PO4	1,998,729	0
NaOH (0.5 M)	21,076,449	0
Sucrose	145,387,619	0
Water	508,568,649	115,416,911
TOTAL	1,728,146,767	1,157,567,197

3. STREAM DETAILS

Stream Name	Air for Drying	S-104	Water for NH4Cl	NH4Cl
**Source**	**INPUT**	**P-3**	**INPUT**	**INPUT**
**Destination**	**P-3**	**P-14**	**P-38**	**P-38**
Stream Properties				
Activity (U/ml)	0.00	0.00	0.00	0.00
Temperature (°C)	25.00	37.66	10.00	20.00
Pressure (bar)	1.01	1.21	1.01	1.01
Density (g/L)	1.18	1.35	1,000.17	1,519.00
Total Enthalpy (kW-h)	11,274.64	16,964.39	934.40	99.03
Specific Enthalpy (kcal/kg)	6.06	9.12	10.07	7.52
Heat Capacity (kcal/kg-°C)	0.24	0.24	1.01	0.38
Component Flowrates (kg/batch)				
Ammonium Chlori	0.00	0.00	0.00	11,337.49
Argon	14,727.78	14,727.78	0.00	0.00
Carb. Dioxide	640.34	640.34	0.00	0.00
Nitrogen	1,250,100.09	1,250,100.09	0.00	0.00
Oxygen	335,377.09	335,377.09	0.00	0.00
Water	0.00	0.00	79,800.96	0.00
TOTAL (kg/batch)	1,600,845.29	1,600,845.29	79,800.96	11,337.49
TOTAL (L/batch)	1,357,570,079.49	1,181,904,804.28	79,787.25	7,463.78

Stream Name	Cl-Solution	S-129	NH4Cl to SFR-1	NH4Cl to SFR-2
**Source**	**P-38**	**P-37**	**P-5**	**P-5**
**Destination**	**P-37**	**P-5**	**P-16**	**P-64**
Stream Properties				
Activity (U/ml)	0.00	0.00	0.00	0.00
Temperature (°C)	10.50	35.00	35.00	35.00
Pressure (bar)	1.01	1.01	1.01	1.01
Density (g/L)	1,044.38	1,035.84	1,035.84	1,035.84
Total Enthalpy (kW-h)	1,033.43	3,428.83	0.65	16.25
Specific Enthalpy (kcal/kg)	9.76	32.37	32.37	32.37
Heat Capacity (kcal/kg-°C)	0.93	0.92	0.92	0.92
Component Flowrates (kg/batch)				
Ammonium Chlori	11,337.49	11,337.49	2.15	53.72
Water	79,800.96	79,800.96	15.16	378.10
TOTAL (kg/batch)	91,138.44	91,138.44	17.32	431.81
TOTAL (L/batch)	87,265.68	87,984.65	16.72	416.87

Stream Name	NH4Cl to SFR-3	NH4Cl to FR-1	Water for NH4SO4	NH4SO4
**Source**	**P-5**	**P-5**	**INPUT**	**INPUT**
**Destination**	**P-65**	**P-4**	**P-36**	**P-36**
Stream Properties				
Activity (U/ml)	0.00	0.00	0.00	0.00
Temperature (°C)	35.00	35.00	10.00	20.00
Pressure (bar)	1.01	1.01	1.01	1.01
Density (g/L)	1,035.84	1,035.84	1,000.17	1,769.00
Total Enthalpy (kW-h)	162.47	3,249.46	1,063.82	2.26
Specific Enthalpy (kcal/kg)	32.37	32.37	10.07	6.80
Heat Capacity (kcal/kg-°C)	0.92	0.92	1.01	0.34
Component Flowrates (kg/batch)				
Amm. Sulfate	0.00	0.00	0.00	285.79
Ammonium Chlori	537.22	10,744.40	0.00	0.00
Water	3,781.29	75,626.41	90,853.00	0.00
TOTAL (kg/batch)	4,318.50	86,370.81	90,853.00	285.79
TOTAL (L/batch)	4,169.06	83,382.00	90,837.39	161.55

Stream Name	SO4-Solution	S-138	Sulfate to SFR-1	Sulfate to SFR-2
**Source**	**P-36**	**P-35**	**P-6**	**P-6**
**Destination**	**P-35**	**P-6**	**P-16**	**P-64**
Stream Properties				
Activity (U/ml)	0.00	0.00	0.00	0.00
Temperature (°C)	10.01	35.00	35.00	35.00
Pressure (bar)	1.01	1.01	1.01	1.01
Density (g/L)	1,001.53	992.43	992.43	992.43
Total Enthalpy (kW-h)	1,066.07	3,710.36	0.70	17.58
Specific Enthalpy (kcal/kg)	10.06	35.03	35.03	35.03
Heat Capacity (kcal/kg-°C)	1.00	1.00	1.00	1.00
Component Flowrates (kg/batch)				
Amm. Sulfate	285.79	285.79	0.05	1.35
Water	90,853.00	90,853.00	17.26	430.46
TOTAL (kg/batch)	91,138.79	91,138.79	17.32	431.82
TOTAL (L/batch)	90,999.30	91,834.17	17.45	435.11

Stream Name	Sulfate to SFR-3	Sulfate to FR-1	Water for NaH2PO4	NaH2PO4
**Source**	**P-6**	**P-6**	**INPUT**	**INPUT**
**Destination**	**P-65**	**P-4**	**P-34**	**P-34**
Stream Properties				
Activity (U/ml)	0.00	0.00	0.00	0.00
Temperature (°C)	35.00	35.00	10.00	20.00
Pressure (bar)	1.01	1.01	1.01	1.01
Density (g/L)	992.43	992.43	1,000.17	2,040.00
Total Enthalpy (kW-h)	175.81	3,516.27	1,031.21	10.70
Specific Enthalpy (kcal/kg)	35.03	35.03	10.07	3.00
Heat Capacity (kcal/kg-°C)	1.00	1.00	1.01	0.15
Component Flowrates (kg/batch)				
Amm. Sulfate	13.54	270.84	0.00	0.00
NaH2PO4	0.00	0.00	0.00	3,070.24
Water	4,304.98	86,100.30	88,068.20	0.00
TOTAL (kg/batch)	4,318.52	86,371.14	88,068.20	3,070.24
TOTAL (L/batch)	4,351.47	87,030.14	88,053.07	1,505.02

Stream Name	PO4-Solution	S-108	Phosphate to SFR-1	Phosphate to SFR-2
**Source**	**P-34**	**P-33**	**P-2**	**P-2**
**Destination**	**P-33**	**P-2**	**P-16**	**P-64**
Stream Properties				
Activity (U/ml)	0.00	0.00	0.00	0.00
Temperature (°C)	10.05	35.00	35.00	35.00
Pressure (bar)	1.01	1.01	1.01	1.01
Density (g/L)	1,017.63	1,008.53	1,008.53	1,008.53
Total Enthalpy (kW-h)	1,041.90	3,611.52	0.69	17.11
Specific Enthalpy (kcal/kg)	9.84	34.10	34.10	34.10
Heat Capacity (kcal/kg-°C)	0.98	0.97	0.97	0.97
Component Flowrates (kg/batch)				
NaH2PO4	3,070.24	3,070.24	0.58	14.55
Water	88,068.20	88,068.20	16.73	417.27
TOTAL (kg/batch)	91,138.44	91,138.44	17.32	431.81
TOTAL (L/batch)	89,559.73	90,367.71	17.17	428.16

Stream Name	Phosphate to SFR-3	Phosphate to FR-1	Salts to SFR-3	Salts to SFR-2
**Source**	**P-2**	**P-2**	**P-65**	**P-64**
**Destination**	**P-65**	**P-4**	**P-15**	**P-1**
Stream Properties				
Activity (U/ml)	0.00	0.00	0.00	0.00
Temperature (°C)	35.00	35.00	35.00	35.00
Pressure (bar)	1.01	1.01	1.01	1.01
Density (g/L)	1,008.53	1,008.53	1,011.95	1,011.95
Total Enthalpy (kW-h)	171.13	3,422.60	509.41	50.94
Specific Enthalpy (kcal/kg)	34.10	34.10	33.83	33.83
Heat Capacity (kcal/kg-°C)	0.97	0.97	0.96	0.96
Component Flowrates (kg/batch)				
Amm. Sulfate	0.00	0.00	13.54	1.35
Ammonium Chlori	0.00	0.00	537.22	53.72
NaH2PO4	145.48	2,909.63	145.48	14.55
Water	4,173.02	83,461.18	12,259.29	1,225.83
TOTAL (kg/batch)	4,318.50	86,370.81	12,955.53	1,295.44
TOTAL (L/batch)	4,281.98	85,640.40	12,802.52	1,280.14

Stream Name	S-123	S-125	S-112	S-118
**Source**	**INPUT**	**P-25**	**INPUT**	**P-21**
**Destination**	**P-25**	**P-24**	**P-21**	**P-20**
Stream Properties				
Activity (U/ml)	0.00	0.00	0.00	0.00
Temperature (°C)	25.00	35.00	25.00	35.00
Pressure (bar)	1.01	1.01	1.01	1.01
Density (g/L)	994.70	991.06	994.70	991.06
Total Enthalpy (kW-h)	6,931.33	9,689.78	629.21	879.61
Specific Enthalpy (kcal/kg)	25.11	35.10	25.11	35.10
Heat Capacity (kcal/kg-°C)	1.00	1.00	1.00	1.00
Component Flowrates (kg/batch)				
Water	237,519.64	237,519.64	21,561.34	21,561.34
TOTAL (kg/batch)	237,519.64	237,519.64	21,561.34	21,561.34
TOTAL (L/batch)	238,784.16	239,662.38	21,676.13	21,755.85

Stream Name	S-120	S-122	Water for 50% Sucrose	Process Sucrose
**Source**	**INPUT**	**P-23**	**INPUT**	**INPUT**
**Destination**	**P-23**	**P-22**	**P-9**	**P-9**
Stream Properties				
Activity (U/ml)	0.00	0.00	0.00	0.00
Temperature (°C)	25.00	35.00	25.00	25.00
Pressure (bar)	1.01	1.01	1.01	1.01
Density (g/L)	994.70	991.06	994.70	1,509.84
Total Enthalpy (kW-h)	70.09	97.98	6,517.23	1,943.25
Specific Enthalpy (kcal/kg)	25.11	35.10	25.11	7.49
Heat Capacity (kcal/kg-°C)	1.00	1.00	1.00	0.30
Component Flowrates (kg/batch)				
Sucrose	0.00	0.00	0.00	223,329.68
Water	2,401.74	2,401.74	223,329.68	0.00
TOTAL (kg/batch)	2,401.74	2,401.74	223,329.68	223,329.68
TOTAL (L/batch)	2,414.53	2,423.41	224,518.65	147,916.45

Stream Name	S-144	S-106	Batch Sucrose	Fed-Batch Sucrose
**Source**	**P-9**	**P-8**	**Sucrose Storage**	**Sucrose Storage**
**Destination**	**P-8**	**Sucrose Storage**	**P-7**	**P-10**
Stream Properties				
Activity (U/ml)	0.00	0.00	0.00	0.00
Temperature (°C)	25.00	35.00	35.00	35.00
Pressure (bar)	1.01	1.01	1.01	1.01
Density (g/L)	1,199.29	1,195.13	1,195.13	1,195.13
Total Enthalpy (kW-h)	8,460.48	11,831.44	965.66	10,865.79
Specific Enthalpy (kcal/kg)	16.30	22.79	22.79	22.79
Heat Capacity (kcal/kg-°C)	0.65	0.65	0.65	0.65
Component Flowrates (kg/batch)				
Sucrose	223,329.68	223,329.68	18,227.72	205,101.95
Water	223,329.68	223,329.68	18,227.72	205,101.95
TOTAL (kg/batch)	446,659.35	446,659.35	36,455.44	410,203.91
TOTAL (L/batch)	372,435.10	373,731.86	30,503.25	343,228.61

Stream Name	Fed-batch Sugar > SFR-1	Fed-Batch Sugar > SFR-2	Fed-Batch Sugar > SFR-3	Fed-Batch Sugar > FR-1
**Source**	**P-10**	**P-10**	**P-10**	**P-10**
**Destination**	**P-16**	**P-1**	**P-15**	**P-4**
Stream Properties				
Activity (U/ml)	0.00	0.00	0.00	0.00
Temperature (°C)	35.00	35.00	35.00	35.00
Pressure (bar)	1.01	1.01	1.01	1.01
Density (g/L)	1,195.13	1,195.13	1,195.13	1,195.13
Total Enthalpy (kW-h)	0.81	9.68	92.51	10,762.78
Specific Enthalpy (kcal/kg)	22.79	22.79	22.79	22.79
Heat Capacity (kcal/kg-°C)	0.65	0.65	0.65	0.65
Component Flowrates (kg/batch)				
Sucrose	15.38	182.75	1,746.24	203,157.59
Water	15.38	182.75	1,746.24	203,157.59
TOTAL (kg/batch)	30.77	365.49	3,492.48	406,315.17
TOTAL (L/batch)	25.74	305.82	2,922.25	339,974.80

Stream Name	S-110	S-124	S-121	S-127
**Source**	**P-7**	**P-7**	**P-7**	**P-7**
**Destination**	**P-12**	**P-22**	**P-20**	**P-24**
Stream Properties				
Activity (U/ml)	0.00	0.00	0.00	0.00
Temperature (°C)	35.00	35.00	35.00	35.00
Pressure (bar)	1.01	1.01	1.01	1.01
Density (g/L)	1,195.13	1,195.13	1,195.13	1,195.13
Total Enthalpy (kW-h)	0.18	4.58	45.76	915.14
Specific Enthalpy (kcal/kg)	22.79	22.79	22.79	22.79
Heat Capacity (kcal/kg-°C)	0.65	0.65	0.65	0.65
Component Flowrates (kg/batch)				
Sucrose	3.46	86.36	863.70	17,274.19
Water	3.46	86.36	863.70	17,274.19
TOTAL (kg/batch)	6.93	172.73	1,727.40	34,548.39
TOTAL (L/batch)	5.80	144.52	1,445.37	28,907.56

Stream Name	Initial Sugar to FR-1	Initial Sugar to SFR-3	Initial Sugar to SFR-2	S-114
**Source**	**P-24**	**P-20**	**P-22**	**INPUT**
**Destination**	**P-4**	**P-15**	**P-1**	**P-18**
Stream Properties				
Activity (U/ml)	0.00	0.00	0.00	0.00
Temperature (°C)	35.00	35.00	35.00	25.00
Pressure (bar)	1.01	1.01	1.01	1.01
Density (g/L)	1,013.02	1,003.77	1,002.54	994.70
Total Enthalpy (kW-h)	10,604.92	925.37	102.56	2.61
Specific Enthalpy (kcal/kg)	33.54	34.19	34.28	25.11
Heat Capacity (kcal/kg-°C)	0.95	0.97	0.98	1.00
Component Flowrates (kg/batch)				
Sucrose	17,274.19	863.70	86.36	0.00
Water	254,793.83	22,425.04	2,488.11	89.57
TOTAL (kg/batch)	272,068.02	23,288.75	2,574.47	89.57
TOTAL (L/batch)	268,569.94	23,201.22	2,567.94	90.04

Stream Name	S-115	Initial Sugar to SFR-1	Air input	S-153
**Source**	**P-18**	**P-12**	**INPUT**	**P-51**
**Destination**	**P-12**	**P-16**	**P-51**	**P-50**
Stream Properties				
Activity (U/ml)	0.00	0.00	0.00	0.00
Temperature (°C)	35.00	35.00	20.00	40.00
Pressure (bar)	1.01	1.01	1.01	6.01
Density (g/L)	991.06	1,003.36	1.20	6.66
Total Enthalpy (kW-h)	3.65	3.84	8,922.14	17,808.73
Specific Enthalpy (kcal/kg)	35.10	34.22	4.85	9.68
Heat Capacity (kcal/kg-°C)	1.00	0.97	0.24	0.24
Component Flowrates (kg/batch)				
Argon	0.00	0.00	14,556.12	14,556.12
Carb. Dioxide	0.00	0.00	632.87	632.87
Nitrogen	0.00	0.00	1,235,530.18	1,235,530.18
Oxygen	0.00	0.00	331,468.27	331,468.27
Sucrose	0.00	3.46	0.00	0.00
Water	89.57	93.03	0.00	0.00
TOTAL (kg/batch)	89.57	96.49	1,582,187.45	1,582,187.45
TOTAL (L/batch)	90.37	96.17	1,319,246,386.83	237,462,908.80

Stream Name	S-139	S-148	S-147	S-146
Source	P-50	P-41	P-41	P-41
Destination	P-41	P-16	P-1	P-15
Stream Properties				
Activity (U/ml)	0.00	0.00	0.00	0.00
Temperature (°C)	40.00	40.00	40.00	40.00
Pressure (bar)	6.01	6.01	6.01	6.01
Density (g/L)	6.66	6.66	6.66	6.66
Total Enthalpy (kW-h)	17,808.73	0.89	20.67	207.02
Specific Enthalpy (kcal/kg)	9.68	9.68	9.68	9.68
Heat Capacity (kcal/kg-°C)	0.24	0.24	0.24	0.24
Component Flowrates (kg/batch)				
Argon	14,556.12	0.73	16.89	169.21
Carb. Dioxide	632.87	0.03	0.73	7.36
Nitrogen	1,235,530.18	61.94	1,433.85	14,362.25
Oxygen	331,468.27	16.62	384.67	3,853.11
TOTAL (kg/batch)	1,582,187.45	79.32	1,836.15	18,391.92
TOTAL (L/batch)	237,462,908.80	11,904.14	275,578.78	2,760,354.90

Stream Name	S-143	Vent SFR-1	Inoculum to SFR-2	Vent FR-1
Source	P-41	P-16	P-16	P-4
Destination	P-4	OUTPUT	P-1	P-49
Stream Properties				
Activity (U/ml)	0.00	0.00	0.00	0.00
Temperature (°C)	40.00	35.00	35.00	34.99
Pressure (bar)	6.01	1.01	1.01	1.01
Density (g/L)	6.66	1.20	993.91	1.17
Total Enthalpy (kW-h)	17,580.15	1.72	6.77	23,972.33
Specific Enthalpy (kcal/kg)	9.68	15.98	35.10	12.25
Heat Capacity (kcal/kg-°C)	0.24	0.24	1.00	0.24
Component Flowrates (kg/batch)				
Amm. Sulfate	0.00	0.00	0.00	0.00
Argon	14,369.30	0.73	0.00	14,388.12
Biomass	0.00	0.00	8.48	0.00
Carb. Dioxide	624.75	13.18	0.00	120,866.73
NaH2PO4	0.00	0.00	0.00	0.00
Nitrogen	1,219,672.14	62.10	0.00	1,221,270.30
Oxygen	327,213.87	16.66	0.00	327,642.63
Sucrose	0.00	0.00	0.00	0.00
Water	0.00	0.00	157.57	0.00
TOTAL (kg/batch)	1,561,880.06	92.67	166.05	1,684,167.79
TOTAL (L/batch)	234,415,070.98	77,250.32	167.07	1,439,774,737.87

Stream Name	Emissions	Vent SFR-2	Inoculum to SFR-3	Vent SFR-3
**Source**	**P-49**	**P-1**	**P-1**	**P-15**
**Destination**	**OUTPUT**	**OUTPUT**	**P-15**	**OUTPUT**
Stream Properties				
Activity (U/ml)	0.00	0.00	0.00	0.00
Temperature (°C)	34.99	35.00	35.00	35.00
Pressure (bar)	1.01	1.01	1.01	1.01
Density (g/L)	1.17	1.18	992.80	1.18
Total Enthalpy (kW-h)	23,972.33	33.63	170.66	333.55
Specific Enthalpy (kcal/kg)	12.25	14.06	35.10	13.95
Heat Capacity (kcal/kg-°C)	0.24	0.24	1.00	0.24
Component Flowrates (kg/batch)				
Ammonium Chlori	0.00	0.00	0.03	0.00
Argon	14,388.12	16.94	0.00	169.67
Biomass	0.00	0.00	129.50	0.00
Carb. Dioxide	120,866.73	218.10	0.00	2,137.79
NaH2PO4	0.00	0.00	0.01	0.00
Nitrogen	1,221,270.30	1,437.83	0.00	14,401.98
Oxygen	327,642.63	385.74	0.00	3,863.77
Sucrose	0.00	0.00	0.17	0.00
Water	0.00	0.00	4,054.25	0.00
TOTAL (kg/batch)	1,684,167.79	2,058.61	4,183.96	20,573.22
TOTAL (L/batch)	1,439,774,737.87	1,738,695.71	4,214.30	17,388,705.77

Stream Name	Inoculum to FR-1	Mother Liquor	S-116	S-128
**Source**	**P-15**	**P-11**	**P-27**	**P-4**
**Destination**	**P-4**	**P-4**	**P-4**	**OUTPUT**
Stream Properties				
Activity (U/ml)	0.00	0.00	0.00	0.00
Temperature (°C)	35.00	9.00	35.31	12.55
Pressure (bar)	1.01	1.01	1.01	1.01
Density (g/L)	992.80	1,003.88	1,012.41	1,004.88
Total Enthalpy (kW-h)	1,704.63	8,362.21	5,109.80	13,478.58
Specific Enthalpy (kcal/kg)	35.10	9.00	34.38	12.50
Heat Capacity (kcal/kg-°C)	1.00	1.00	0.97	0.99
Component Flowrates (kg/batch)				
Amm. Sulfate	0.00	6.24	0.37	6.61
Ammonium Chlori	0.02	247.65	14.60	262.25
Biomass	1,303.98	0.00	25,437.19	25,437.19
NaH2PO4	0.00	67.04	3.95	71.00
pHBA (aq)	0.00	1,591.44	4,691.50	6,282.94
pHBA (solid)	0.00	1,559.61	0.00	1,559.61
Sucrose	0.18	5,078.93	299.45	5,378.38
Water	40,484.82	791,063.63	97,451.28	888,514.91
TOTAL (kg/batch)	41,789.00	799,614.54	127,898.35	927,512.89
TOTAL (L/batch)	42,092.07	796,523.78	126,329.98	923,010.22

Stream Name	S-113	S-105	Vent R-101	S-101
**Source**	**P-4**	**P-27**	**P-28**	**P-28**
**Destination**	**P-27**	**P-28**	**OUTPUT**	**P-11**
Stream Properties				
Activity (U/ml)	0.00	0.00	0.00	0.00
Temperature (°C)	35.00	35.31	5.00	5.00
Pressure (bar)	1.01	1.01	1.01	1.01
Density (g/L)	1,020.10	1,021.31	1.26	1,033.71
Total Enthalpy (kW-h)	32,361.46	27,537.98	1.18	3,921.69
Specific Enthalpy (kcal/kg)	32.41	32.41	1.23	4.62
Heat Capacity (kcal/kg-°C)	0.92	0.91	0.24	0.92
Component Flowrates (kg/batch)				
Amm. Sulfate	6.61	6.24	0.00	6.24
Ammonium Chlori	262.25	247.65	0.00	247.65
Argon	0.00	0.00	7.60	0.00
Biomass	25,437.19	0.00	0.00	0.00
Carb. Dioxide	0.00	0.00	0.33	0.00
NaH2PO4	71.00	67.04	0.00	67.04
Nitrogen	0.00	0.00	645.48	0.00
Oxygen	0.00	0.00	173.17	0.00
pHBA (aq)	84,263.44	79,571.94	0.00	1,591.44
pHBA (solid)	0.00	0.00	0.00	77,980.50
Sucrose	5,378.38	5,078.93	0.00	5,078.93
Water	743,624.12	646,172.84	0.00	646,172.84
TOTAL (kg/batch)	859,042.99	731,144.64	826.58	731,144.64
TOTAL (L/batch)	842,119.07	715,890.60	653,946.64	707,304.61

Stream Name	Wash Water	S-102	Humid Air	Final Product
**Source**	**INPUT**	**P-11**	**P-14**	**P-14**
**Destination**	**P-11**	**P-14**	**OUTPUT**	**OUTPUT**
Stream Properties				
Activity (U/ml)	0.00	0.00	0.00	0.00
Temperature (°C)	25.00	22.93	50.00	50.00
Pressure (bar)	1.01	2.01	1.01	1.01
Density (g/L)	994.70	1,211.06	1.08	1,303.70
Total Enthalpy (kW-h)	5,173.75	1,414.30	46,082.66	1,215.00
Specific Enthalpy (kcal/kg)	25.11	11.18	24.28	13.61
Heat Capacity (kcal/kg-°C)	1.00	0.49	0.25	0.27
Component Flowrates (kg/batch)				
Amm. Sulfate	0.00	0.00	0.00	0.00
Ammonium Chlori	0.00	0.00	0.00	0.00
Argon	0.00	0.00	14,727.78	0.00
Carb. Dioxide	0.00	0.00	640.34	0.00
NaH2PO4	0.00	0.00	0.00	0.00
Nitrogen	0.00	0.00	1,250,100.09	0.00
Oxygen	0.00	0.00	335,377.09	0.00
pHBA (aq)	0.00	0.00	0.00	0.00
pHBA (solid)	0.00	76,420.89	0.00	76,420.89
Sucrose	0.00	0.00	0.00	0.00
Water	177,291.72	32,400.93	32,016.91	384.02
TOTAL (kg/batch)	177,291.72	108,821.82	1,632,862.20	76,804.92
TOTAL (L/batch)	178,235.60	89,856.45	1,518,529,211.37	58,912.90

4. OVERALL COMPONENT BALANCE (kg/batch)

COMPONENT	INITIAL	INPUT	OUTPUT	FINAL	IN-OUT
Amm. Sulfate	0.00	285.79	6.61	0.00	279.18
Ammonium Chlori	0.00	11,337.49	262.25	0.00	11,075.24
Argon	21.89	29,283.90	29,310.85	14.23	- 19.29
Biomass	0.00	0.00	25,437.19	0.00	- 25,437.19
Carb. Dioxide	0.95	1,273.21	123,876.48	2.86	- 122,605.17
NaH2PO4	0.00	3,070.24	71.00	0.00	2,999.25
Nitrogen	1,858.23	2,485,630.27	2,487,917.78	1,207.98	- 1,637.26
Oxygen	498.53	666,845.36	667,459.05	324.08	- 439.24
pHBA (aq)	0.00	0.00	6,282.94	0.00	- 6,282.94
pHBA (solid)	0.00	0.00	77,980.50	0.00	- 77,980.50
Phosphoric Acid	0.00	416.11	416.11	0.00	0.00
Sodium Hydroxid	0.00	634.56	634.56	0.00	0.00
Sucrose	0.00	223,329.68	5,378.38	0.00	217,951.29
Water	0.00	1,010,633.73	1,010,633.73	0.00	0.00
TOTAL	2,379.60	4,432,740.34	4,435,667.43	1,549.14	2,096.63
				Overall Error:	0,047%

5. EQUIPMENT CONTENTS

SFR-3				
**Procedure**	**Operation**	**Time (in h)**	**Volume (in L)**	**Vapor (in kg)**
P-15	START	25.61	0.00	62.04(*)
P-15	TRANSFER-IN-SALTS (Transfer In)	26.61	12,802.48	62.04(*)
P-15	TRANSFER-IN-INITIAL-SUGAR (Transfer In)	27.61	36,003.68	62.04(*)
P-15	TRANSFER-IN-INOCULUM (Transfer In)	28.11	40,218.00	62.04(*)
P-15	FERMENT-2 (Batch Stoich. Fermentation)	40.11	42,092.07	12.45(*)
P-15	TRANSFER-OUT-1 (Transfer Out)	41.11	0.00	12.45(*)
P-15	CIP-1 (In-Place-Cleaning)	43.19	0.00	12.45(*)
P-15	SIP-1 (In-Place-Steaming)	45.19	0.00	12.45(*)

(*) Contains material in vapor phase other than Oxygen & Nitrogen

SFR-2				
**Procedure**	**Operation**	**Time (in h)**	**Volume (in L)**	**Vapor (in kg)**
P-1	START	14.11	0.00	6.21(*)
P-1	TRANSFER-IN-SALTS (Transfer In)	14.61	1,280.14	6.21(*)
P-1	TRANSFER-IN-INITIAL-SUGAR (Transfer In)	15.11	3,848.07	6.21(*)
P-1	TRANSFER-IN-INOCULUM (Transfer In)	15.61	4,015.15	6.21(*)
P-1	FERMENT-1 (Batch Stoich. Fermentation)	27.61	4,214.30	1.25(*)
P-1	TRANSFER-OUT-1 (Transfer Out)	28.11	0.00	1.25(*)
P-1	CIP-1 (In-Place-Cleaning)	30.19	0.00	1.25(*)
P-1	SIP-1 (In-Place-Steaming)	31.19	0.00	1.25(*)

(*) Contains material in vapor phase other than Oxygen & Nitrogen

SFR-1				
**Procedure**	**Operation**	**Time (in h)**	**Volume (in L)**	**Vapor (in kg)**
P-16	START	0.00	0.00	0.25(*)
P-16	TRANSFER-IN-PHOSPHATE (Transfer In)	0.25	17.17	0.25(*)
P-16	TRANSFER-IN-SULFATE (Transfer In)	0.50	34.62	0.25(*)
P-16	TRANSFER-IN-NH4Cl (Transfer In)	0.75	51.34	0.25(*)
P-16	TRANSFER-IN-INITIAL-SUGAR (Transfer In)	1.00	147.51	0.25(*)
P-16	FERMENT (Batch Stoich. Fermentation)	15.11	167.07	0.05(*)
P-16	TRANSFER-OUT (Transfer Out)	15.61	0.00	0.05(*)
P-16	CIP-1 (In-Place-Cleaning)	17.69	0.00	0.05(*)
P-16	SIP-1 (In-Place-Steaming)	18.19	0.00	0.05(*)

(*) Contains material in vapor phase other than Oxygen & Nitrogen

FR-1				
**Procedure**	**Operation**	**Time (in h)**	**Volume (in L)**	**Vapor (in kg)**
P-4	START	39.11	0.00	1,161.43(*)
P-4	TRANSFER-IN-SULFATE (Transfer In)	40.11	87,029.21	1,161.43(*)
P-4	TRANSFER-IN-NH4Cl (Transfer In)	40.11	170,411.26	1,161.43(*)
P-4	TRANSFER-IN-PHOSPHATE (Transfer In)	40.11	256,051.48	1,161.43(*)
P-4	TRANSFER-IN-INITIAL-SUGAR (Transfer In)	40.11	524,621.38	1,161.43(*)
P-4	TRANSFER-IN-INOCULUM (Transfer In)	41.11	566,713.95	1,161.43(*)
P-4	FERMENT-1 (Batch Stoich. Fermentation)	112.43	842,119.07	167.05(*)
P-4	TRANSFER-OUT-2 (Transfer Out)	76.11	0.00	1,127.85(*)
P-4	TRANSFER-IN-1 (Transfer In)	76.11	126,329.97	982.20(*)
P-4	TRANSFER-IN-2 (Transfer In)	76.11	923,010.22	76.48(*)
P-4	TRANSFER-OUT-1 (Transfer Out)	114.43	0.00	1,212.31(*)
P-4	CIP-1 (In-Place-Cleaning)	116.51	0.00	1,212.31(*)
P-4	SIP-1 (In-Place-Steaming)	118.51	0.00	1,212.31(*)

(*) Contains material in vapor phase other than Oxygen & Nitrogen

R-102				
**Procedure**	**Operation**	**Time (in h)**	**Volume (in L)**	**Vapor (in kg)**
P-28	START	40.11	0.00	78.16(*)
	AFTER AUTO-INIT	40.11	59,657.55	78.16(*)
P-28	REACT-1 (Batch Stoich. Reaction)	112.11	58,942.05	9.28(*)
P-28	END	112.11	0.00	9.28(*)

(*) Contains material in vapor phase other than Oxygen & Nitrogen

BCFBD-101				
**Procedure**	**Operation**	**Time (in h)**	**Volume (in L)**	**Vapor (in kg)**
P-11	START	40.11	0.00	8.82(*)
P-11	FILTER-1 (Cloth Filtration)	111.61	3,713.24	8.82(*)
P-11	CAKE-WASH-1 (Cake Wash)	111.86	3,744.02	8.82(*)
P-11	TRANSFER-OUT-1 (Transfer Out)	112.11	0.00	8.82(*)

(*) Contains material in vapor phase other than Oxygen & Nitrogen
