# Supplementary material for: Economic Process Evaluation and Environmental Life-Cycle Assessment of Bio-Aromatics Production
Source: Front Bioeng Biotechnol. 2020 May 13;8:403. doi: 10.3389/fbioe.2020.00403 (PMC7237583; doi:10.3389/fbioe.2020.00403)
Supplement: Supplementary file 1 [file Data_Sheet_1.zip › Sc_5.pdf]

Materials & Streams Report

März 20, 2020

for Supplementary_5_bacterial_best_case_cane_sugar

1. OVERALL PROCESS DATA

Annual Operating Time	7,916.44 h
Unit Production Ref. Rate	10,000,000.00 kg MP/yr
Batch Size	15,337.42 kg MP
Recipe Batch Time	104.44 h
Recipe Cycle Time	12.00 h
Number of Batches per Year	652.00

MP = Total Flow of Stream 'Final Product'

2.1 STARTING MATERIAL REQUIREMENTS (per Section)

Section	Starting Material	Active Product	Amount Needed (kg Sin/kg MP)	Molar Yield (%)	Mass Yield (%)	Gross Mass Yield (%)
Fermentation Section	(none)	(none)	0.00	Unknown	Unknown	Unknown
Downstream Section	(none)	(none)	0.00	Unknown	Unknown	Unknown

Sin = Section Starting Material, Aout = Section Active Product

2.2 BULK MATERIALS (Entire Process)

Material	kg/yr	kg/batch	kg/kg MP
Air	294,222,950	451,262.19	29.42
Amm. Sulfate	35,642	54.67	0.00
Ammonium Chlori	1,413,929	2,168.60	0.14
Ca Hydroxide	2,832,921	4,344.97	0.28
H3PO4 (2%)	5,900,268	9,049.49	0.59
HNO3 (70%)	7,047,516	10,809.07	0.70
NaH2PO4	382,899	587.27	0.04
NaOH (0.5 M)	8,156,393	12,509.81	0.82
Sucrose	27,852,048	42,717.86	2.79
Water	156,190,567	239,556.08	15.62
TOTAL	504,035,133	773,060.02	50.40

2.3 BULK MATERIALS (per Section)

SECTIONS IN: Main Branch

Fermentation Section

Material	kg/yr	kg/batch	kg/kg MP
Air	101,619,756	155,858.52	10.16
Amm. Sulfate	35,642	54.67	0.00
Ammonium Chlori	1,413,929	2,168.60	0.14
Ca Hydroxide	2,832,921	4,344.97	0.28
H3PO4 (2%)	5,900,268	9,049.49	0.59
NaH2PO4	382,899	587.27	0.04
NaOH (0.5 M)	8,156,393	12,509.81	0.82
Sucrose	27,852,048	42,717.86	2.79
Water	102,609,974	157,377.26	10.26
TOTAL	250,803,829	384,668.45	25.08

Downstream Section

Material	kg/yr	kg/batch	kg/kg MP
Air	192,603,194	295,403.67	19.26
HNO3 (70%)	7,047,516	10,809.07	0.70
Water	53,580,593	82,178.82	5.36
TOTAL	253,231,304	388,391.57	25.32

2.4 BULK MATERIALS (per Material)

Air

Procedure	% Total	kg/yr	kg/batch	kg/kg MP
Fermentation Section (Main Branch)				
P-51	34.54	101,619,756	155,858.52	10.16
Downstream Section (Main Branch)				
P-27	65.46	192,603,194	295,403.67	19.26
TOTAL	100.00	294,222,950	451,262.19	29.42

Amm. Sulfate

Procedure	% Total	kg/yr	kg/batch	kg/kg MP
Fermentation Section (Main Branch)				
P-36	100.00	35,642	54.67	0.00
TOTAL	100.00	35,642	54.67	0.00

Ammonium Chlori

Procedure	% Total	kg/yr	kg/batch	kg/kg MP
Fermentation Section (Main Branch)				
P-38	100.00	1,413,929	2,168.60	0.14
TOTAL	100.00	1,413,929	2,168.60	0.14

Ca Hydroxide

Procedure	% Total	kg/yr	kg/batch	kg/kg MP
Fermentation Section (Main Branch)				
P-4	94.38	2,673,691	4,100.75	0.27
P-1	0.51	14,423	22.12	0.00
P-15	5.09	144,231	221.21	0.01
P-16	0.02	576	0.88	0.00
TOTAL	100.00	2,832,921	4,344.97	0.28

H3PO4 (2%)

Procedure	% Total	kg/yr	kg/batch	kg/kg MP
Fermentation Section (Main Branch)				
P-4	46.04	2,716,518	4,166.44	0.27
P-1	8.47	500,023	766.91	0.05
P-15	42.60	2,513,253	3,854.68	0.25
P-16	2.89	170,474	261.46	0.02
TOTAL	100.00	5,900,268	9,049.49	0.59

HNO3 (70%)

Procedure	% Total	kg/yr	kg/batch	kg/kg MP
Downstream Section (Main Branch)				
P-3	100.00	7,047,516	10,809.07	0.70
TOTAL	100.00	7,047,516	10,809.07	0.70

NaH2PO4

Procedure	% Total	kg/yr	kg/batch	kg/kg MP
Fermentation Section (Main Branch)				
P-34	100.00	382,899	587.27	0.04
TOTAL	100.00	382,899	587.27	0.04

NaOH (0.5 M)

Procedure	% Total	kg/yr	kg/batch	kg/kg MP
Fermentation Section (Main Branch)				
P-4	78.39	6,393,606	9,806.14	0.64
P-1	6.18	504,366	773.57	0.05
P-15	13.32	1,086,465	1,666.36	0.11
P-16	2.11	171,955	263.74	0.02
TOTAL	100.00	8,156,393	12,509.81	0.82

Sucrose				
**Procedure**	**% Total**	**kg/yr**	**kg/batch**	**kg/kg MP**
Fermentation Section (Main Branch)				
P-9	100.00	27,852,048	42,717.86	2.79
TOTAL	100.00	27,852,048	42,717.86	2.79

Water				
**Procedure**	**% Total**	**kg/yr**	**kg/batch**	**kg/kg MP**
Fermentation Section (Main Branch)				
P-4	3.85	6,006,478	9,212.39	0.60
P-34	7.03	10,983,223	16,845.43	1.10
P-36	7.25	11,330,523	17,378.10	1.13
P-38	6.37	9,952,193	15,264.10	1.00
P-9	17.83	27,852,048	42,717.86	2.79
P-18	0.01	11,170	17.13	0.00
P-21	1.72	2,688,973	4,124.19	0.27
P-23	0.19	299,528	459.40	0.03
P-25	18.97	29,621,716	45,432.08	2.96
P-1	0.71	1,105,597	1,695.70	0.11
P-15	1.52	2,381,588	3,652.74	0.24
P-16	0.24	376,935	578.12	0.04
Downstream Section (Main Branch)				
P-26	19.93	31,130,267	47,745.81	3.11
P-11	14.37	22,450,327	34,433.02	2.25
TOTAL	100.00	156,190,567	239,556.08	15.62

2.5 BULK MATERIALS: SECTION TOTALS (kg/kg MP)

Raw Material	Fermentation Section	Downstream Section
Air	10.16	19.26
Amm. Sulfate	0.00	0.00
Ammonium Chlori	0.14	0.00
Ca Hydroxide	0.28	0.00
H3PO4 (2%)	0.59	0.00
HNO3 (70%)	0.00	0.70
NaH2PO4	0.04	0.00
NaOH (0.5 M)	0.82	0.00
Sucrose	2.79	0.00
Water	10.26	5.36
TOTAL	25.08	25.32

2.6 BULK MATERIALS: SECTION TOTALS (kg/batch)

Raw Material	Fermentation Section	Downstream Section
Air	155,858.52	295,403.67
Amm. Sulfate	54.67	0.00
Ammonium Chlori	2,168.60	0.00
Ca Hydroxide	4,344.97	0.00
H3PO4 (2%)	9,049.49	0.00
HNO3 (70%)	0.00	10,809.07
NaH2PO4	587.27	0.00
NaOH (0.5 M)	12,509.81	0.00
Sucrose	42,717.86	0.00
Water	157,377.26	82,178.82
TOTAL	384,668.45	388,391.57

2.7 BULK MATERIALS: SECTION TOTALS (kg/yr)

Raw Material	Fermentation Section	Downstream Section
Air	101,619,756	192,603,194
Amm. Sulfate	35,642	0
Ammonium Chlori	1,413,929	0
Ca Hydroxide	2,832,921	0
H3PO4 (2%)	5,900,268	0
HNO3 (70%)	0	7,047,516
NaH2PO4	382,899	0
NaOH (0.5 M)	8,156,393	0
Sucrose	27,852,048	0
Water	102,609,974	53,580,593
TOTAL	250,803,829	253,231,304

3. STREAM DETAILS

Stream Name	Air for Drying	S-116	Water for NH4Cl	NH4Cl
Source	INPUT	P-27	INPUT	INPUT
Destination	P-27	P-14	P-38	P-38
Stream Properties				
Activity (U/ml)	0.00	0.00	0.00	0.00
Temperature (°C)	25.00	37.66	10.00	20.00
Pressure (bar)	1.01	1.21	1.01	1.01
Density (g/L)	1.18	1.35	1,000.17	1,519.00
Total Enthalpy (kW-h)	2,080.51	3,130.44	178.73	18.94
Specific Enthalpy (kcal/kg)	6.06	9.12	10.07	7.52
Heat Capacity (kcal/kg-°C)	0.24	0.24	1.01	0.38
Component Flowrates (kg/batch)				
Ammonium Chlori	0.00	0.00	0.00	2,168.60
Argon	2,717.71	2,717.71	0.00	0.00
Carb. Dioxide	118.16	118.16	0.00	0.00
Nitrogen	230,680.73	230,680.73	0.00	0.00
Oxygen	61,887.07	61,887.07	0.00	0.00
Water	0.00	0.00	15,264.10	0.00
TOTAL (kg/batch)	295,403.67	295,403.67	15,264.10	2,168.60
TOTAL (L/batch)	250,512,144.30	218,096,665.03	15,261.48	1,427.65

Stream Name	Cl-Solution	S-129	NH4Cl to SFR-1	NH4Cl to SFR-2
Source	P-38	P-37	P-5	P-5
Destination	P-37	P-5	P-16	P-64
Stream Properties				
Activity (U/ml)	0.00	0.00	0.00	0.00
Temperature (°C)	10.50	35.00	35.00	35.00
Pressure (bar)	1.01	1.01	1.01	1.01
Density (g/L)	1,044.38	1,035.84	1,035.84	1,035.84
Total Enthalpy (kW-h)	197.67	655.86	0.12	3.11
Specific Enthalpy (kcal/kg)	9.76	32.37	32.37	32.37
Heat Capacity (kcal/kg-°C)	0.93	0.92	0.92	0.92
Component Flowrates (kg/batch)				
Ammonium Chlori	2,168.60	2,168.60	0.41	10.27
Water	15,264.10	15,264.10	2.90	72.32
TOTAL (kg/batch)	17,432.70	17,432.70	3.31	82.60
TOTAL (L/batch)	16,691.93	16,829.45	3.20	79.74

Stream Name	NH4Cl to SFR-3	NH4Cl to FR-1	Water for NH4SO4	NH4SO4
**Source**	**P-5**	**P-5**	**INPUT**	**INPUT**
**Destination**	**P-65**	**P-4**	**P-36**	**P-36**
Stream Properties				
Activity (U/ml)	0.00	0.00	0.00	0.00
Temperature (°C)	35.00	35.00	10.00	20.00
Pressure (bar)	1.01	1.01	1.01	1.01
Density (g/L)	1,035.84	1,035.84	1,000.17	1,769.00
Total Enthalpy (kW-h)	31.08	621.55	203.48	0.43
Specific Enthalpy (kcal/kg)	32.37	32.37	10.07	6.80
Heat Capacity (kcal/kg-°C)	0.92	0.92	1.01	0.34
Component Flowrates (kg/batch)				
Amm. Sulfate	0.00	0.00	0.00	54.67
Ammonium Chlori	102.76	2,055.16	0.00	0.00
Water	723.27	14,465.60	17,378.10	0.00
TOTAL (kg/batch)	826.03	16,520.76	17,378.10	54.67
TOTAL (L/batch)	797.45	15,949.07	17,375.12	30.90

Stream Name	SO4-Solution	S-138	Sulfate to SFR-1	Sulfate to SFR-2
**Source**	**P-36**	**P-35**	**P-6**	**P-6**
**Destination**	**P-35**	**P-6**	**P-16**	**P-64**
Stream Properties				
Activity (U/ml)	0.00	0.00	0.00	0.00
Temperature (°C)	10.01	35.00	35.00	35.00
Pressure (bar)	1.01	1.01	1.01	1.01
Density (g/L)	1,001.53	992.43	992.43	992.43
Total Enthalpy (kW-h)	203.92	709.71	0.13	3.36
Specific Enthalpy (kcal/kg)	10.06	35.03	35.03	35.03
Heat Capacity (kcal/kg-°C)	1.00	1.00	1.00	1.00
Component Flowrates (kg/batch)				
Amm. Sulfate	54.67	54.67	0.01	0.26
Water	17,378.10	17,378.10	3.30	82.34
TOTAL (kg/batch)	17,432.77	17,432.77	3.31	82.60
TOTAL (L/batch)	17,406.09	17,565.78	3.34	83.23

Stream Name	Sulfate to SFR-3	Sulfate to FR-1	Water for NaH2PO4	NaH2PO4
**Source**	**P-6**	**P-6**	**INPUT**	**INPUT**
**Destination**	**P-65**	**P-4**	**P-34**	**P-34**
Stream Properties				
Activity (U/ml)	0.00	0.00	0.00	0.00
Temperature (°C)	35.00	35.00	10.00	20.00
Pressure (bar)	1.01	1.01	1.01	1.01
Density (g/L)	992.43	992.43	1,000.17	2,040.00
Total Enthalpy (kW-h)	33.63	672.58	197.25	2.05
Specific Enthalpy (kcal/kg)	35.03	35.03	10.07	3.00
Heat Capacity (kcal/kg-°C)	1.00	1.00	1.01	0.15
Component Flowrates (kg/batch)				
Amm. Sulfate	2.59	51.81	0.00	0.00
NaH2PO4	0.00	0.00	0.00	587.27
Water	823.44	16,469.02	16,845.43	0.00
TOTAL (kg/batch)	826.03	16,520.83	16,845.43	587.27
TOTAL (L/batch)	832.34	16,646.88	16,842.54	287.88

Stream Name	PO4-Solution	S-108	Phosphate to SFR-1	Phosphate to SFR-2
**Source**	**P-34**	**P-33**	**P-2**	**P-2**
**Destination**	**P-33**	**P-2**	**P-16**	**P-64**
Stream Properties				
Activity (U/ml)	0.00	0.00	0.00	0.00
Temperature (°C)	10.05	35.00	35.00	35.00
Pressure (bar)	1.01	1.01	1.01	1.01
Density (g/L)	1,017.63	1,008.53	1,008.53	1,008.53
Total Enthalpy (kW-h)	199.29	690.80	0.13	3.27
Specific Enthalpy (kcal/kg)	9.84	34.10	34.10	34.10
Heat Capacity (kcal/kg-°C)	0.98	0.97	0.97	0.97
Component Flowrates (kg/batch)				
NaH2PO4	587.27	587.27	0.11	2.78
Water	16,845.43	16,845.43	3.20	79.81
TOTAL (kg/batch)	17,432.70	17,432.70	3.31	82.60
TOTAL (L/batch)	17,130.73	17,285.28	3.28	81.90

Stream Name	Phosphate to SFR-3	Phosphate to FR-1	Salts to SFR-3	Salts to SFR-2
**Source**	**P-2**	**P-2**	**P-65**	**P-64**
**Destination**	**P-65**	**P-4**	**P-15**	**P-1**
Stream Properties				
Activity (U/ml)	0.00	0.00	0.00	0.00
Temperature (°C)	35.00	35.00	35.00	35.00
Pressure (bar)	1.01	1.01	1.01	1.01
Density (g/L)	1,008.53	1,008.53	1,011.95	1,011.95
Total Enthalpy (kW-h)	32.73	654.66	97.44	9.74
Specific Enthalpy (kcal/kg)	34.10	34.10	33.83	33.83
Heat Capacity (kcal/kg-°C)	0.97	0.97	0.96	0.96
Component Flowrates (kg/batch)				
Amm. Sulfate	0.00	0.00	2.59	0.26
Ammonium Chlori	0.00	0.00	102.76	10.27
NaH2PO4	27.83	556.55	27.83	2.78
Water	798.20	15,964.22	2,344.92	234.47
TOTAL (kg/batch)	826.03	16,520.76	2,478.10	247.79
TOTAL (L/batch)	819.05	16,381.05	2,448.83	244.86

Stream Name	S-123	S-125	S-112	S-118
**Source**	**INPUT**	**P-25**	**INPUT**	**P-21**
**Destination**	**P-25**	**P-24**	**P-21**	**P-20**
Stream Properties				
Activity (U/ml)	0.00	0.00	0.00	0.00
Temperature (°C)	25.00	35.00	25.00	35.00
Pressure (bar)	1.01	1.01	1.01	1.01
Density (g/L)	994.70	991.06	994.70	991.06
Total Enthalpy (kW-h)	1,325.80	1,853.43	120.35	168.25
Specific Enthalpy (kcal/kg)	25.11	35.10	25.11	35.10
Heat Capacity (kcal/kg-°C)	1.00	1.00	1.00	1.00
Component Flowrates (kg/batch)				
Water	45,432.08	45,432.08	4,124.19	4,124.19
TOTAL (kg/batch)	45,432.08	45,432.08	4,124.19	4,124.19
TOTAL (L/batch)	45,673.95	45,841.94	4,146.15	4,161.40

Stream Name	S-120	S-122	Water for 50% Sucrose	Process Sucrose
Source	INPUT	P-23	INPUT	INPUT
Destination	P-23	P-22	P-9	P-9
Stream Properties				
Activity (U/ml)	0.00	0.00	0.00	0.00
Temperature (°C)	25.00	35.00	25.00	25.00
Pressure (bar)	1.01	1.01	1.01	1.01
Density (g/L)	994.70	991.06	994.70	1,509.84
Total Enthalpy (kW-h)	13.41	18.74	1,246.60	371.70
Specific Enthalpy (kcal/kg)	25.11	35.10	25.11	7.49
Heat Capacity (kcal/kg-°C)	1.00	1.00	1.00	0.30
Component Flowrates (kg/batch)				
Sucrose	0.00	0.00	0.00	42,717.86
Water	459.40	459.40	42,717.86	0.00
TOTAL (kg/batch)	459.40	459.40	42,717.86	42,717.86
TOTAL (L/batch)	461.84	463.54	42,945.29	28,293.04

Stream Name	S-144	S-106	Batch Sucrose	Fed-Batch Sucrose
Source	P-9	P-8	Sucrose Storage	Sucrose Storage
Destination	P-8	Sucrose Storage	P-7	P-10
Stream Properties				
Activity (U/ml)	0.00	0.00	0.00	0.00
Temperature (°C)	25.00	35.00	35.00	35.00
Pressure (bar)	1.01	1.01	1.01	1.01
Density (g/L)	1,199.29	1,195.13	1,195.13	1,195.13
Total Enthalpy (kW-h)	1,618.30	2,263.08	184.71	2,078.38
Specific Enthalpy (kcal/kg)	16.30	22.79	22.79	22.79
Heat Capacity (kcal/kg-°C)	0.65	0.65	0.65	0.65
Component Flowrates (kg/batch)				
Sucrose	42,717.86	42,717.86	3,486.55	39,231.32
Water	42,717.86	42,717.86	3,486.55	39,231.32
TOTAL (kg/batch)	85,435.73	85,435.73	6,973.09	78,462.64
TOTAL (L/batch)	71,238.33	71,486.37	5,834.57	65,651.79

Stream Name	Fed-batch Sugar > SFR-1	Fed-Batch Sugar > SFR-2	Fed-Batch Sugar > SFR-3	Fed-Batch Sugar > FR-1
**Source**	**P-10**	**P-10**	**P-10**	**P-10**
**Destination**	**P-16**	**P-1**	**P-15**	**P-4**
Stream Properties				
Activity (U/ml)	0.00	0.00	0.00	0.00
Temperature (°C)	35.00	35.00	35.00	35.00
Pressure (bar)	1.01	1.01	1.01	1.01
Density (g/L)	1,195.13	1,195.13	1,195.13	1,195.13
Total Enthalpy (kW-h)	0.16	1.85	17.70	2,058.67
Specific Enthalpy (kcal/kg)	22.79	22.79	22.79	22.79
Heat Capacity (kcal/kg-°C)	0.65	0.65	0.65	0.65
Component Flowrates (kg/batch)				
Sucrose	2.94	34.96	334.02	38,859.41
Water	2.94	34.96	334.02	38,859.41
TOTAL (kg/batch)	5.88	69.91	668.03	77,718.81
TOTAL (L/batch)	4.92	58.50	558.96	65,029.41

Stream Name	S-110	S-124	S-121	S-127
**Source**	**P-7**	**P-7**	**P-7**	**P-7**
**Destination**	**P-12**	**P-22**	**P-20**	**P-24**
Stream Properties				
Activity (U/ml)	0.00	0.00	0.00	0.00
Temperature (°C)	35.00	35.00	35.00	35.00
Pressure (bar)	1.01	1.01	1.01	1.01
Density (g/L)	1,195.13	1,195.13	1,195.13	1,195.13
Total Enthalpy (kW-h)	0.04	0.88	8.75	175.05
Specific Enthalpy (kcal/kg)	22.79	22.79	22.79	22.79
Heat Capacity (kcal/kg-°C)	0.65	0.65	0.65	0.65
Component Flowrates (kg/batch)				
Sucrose	0.66	16.52	165.21	3,304.16
Water	0.66	16.52	165.21	3,304.16
TOTAL (kg/batch)	1.32	33.04	330.41	6,608.32
TOTAL (L/batch)	1.11	27.64	276.47	5,529.36

Stream Name	Initial Sugar to FR-1	Initial Sugar to SFR-3	Initial Sugar to SFR-2	S-114
**Source**	**P-24**	**P-20**	**P-22**	**INPUT**
**Destination**	**P-4**	**P-15**	**P-1**	**P-18**
Stream Properties				
Activity (U/ml)	0.00	0.00	0.00	0.00
Temperature (°C)	35.00	35.00	35.00	25.00
Pressure (bar)	1.01	1.01	1.01	1.01
Density (g/L)	1,013.02	1,003.77	1,002.54	994.70
Total Enthalpy (kW-h)	2,028.48	177.00	19.62	0.50
Specific Enthalpy (kcal/kg)	33.54	34.19	34.28	25.11
Heat Capacity (kcal/kg-°C)	0.95	0.97	0.98	1.00
Component Flowrates (kg/batch)				
Sucrose	3,304.16	165.21	16.52	0.00
Water	48,736.24	4,289.40	475.92	17.13
TOTAL (kg/batch)	52,040.40	4,454.61	492.44	17.13
TOTAL (L/batch)	51,371.29	4,437.86	491.19	17.22

Stream Name	S-115	Initial Sugar to SFR-1	Air input	S-153
**Source**	**P-18**	**P-12**	**INPUT**	**P-51**
**Destination**	**P-12**	**P-16**	**P-51**	**P-50**
Stream Properties				
Activity (U/ml)	0.00	0.00	0.00	0.00
Temperature (°C)	35.00	35.00	20.00	40.00
Pressure (bar)	1.01	1.01	1.01	6.01
Density (g/L)	991.06	1,003.36	1.20	6.66
Total Enthalpy (kW-h)	0.70	0.73	878.90	1,754.31
Specific Enthalpy (kcal/kg)	35.10	34.22	4.85	9.68
Heat Capacity (kcal/kg-°C)	1.00	0.97	0.24	0.24
Component Flowrates (kg/batch)				
Argon	0.00	0.00	1,433.90	1,433.90
Carb. Dioxide	0.00	0.00	62.34	62.34
Nitrogen	0.00	0.00	121,709.92	121,709.92
Oxygen	0.00	0.00	32,652.36	32,652.36
Sucrose	0.00	0.66	0.00	0.00
Water	17.13	17.79	0.00	0.00
TOTAL (kg/batch)	17.13	18.46	155,858.52	155,858.52
TOTAL (L/batch)	17.29	18.40	129,956,656.48	23,392,056.23

Stream Name	S-139	S-148	S-147	S-146
**Source**	**P-50**	**P-41**	**P-41**	**P-41**
**Destination**	**P-41**	**P-16**	**P-1**	**P-15**
Stream Properties				
Activity (U/ml)	0.00	0.00	0.00	0.00
Temperature (°C)	40.00	40.00	40.00	40.00
Pressure (bar)	6.01	6.01	6.01	6.01
Density (g/L)	6.66	6.66	6.66	6.66
Total Enthalpy (kW-h)	1,754.31	0.17	3.96	39.65
Specific Enthalpy (kcal/kg)	9.68	9.68	9.68	9.68
Heat Capacity (kcal/kg-°C)	0.24	0.24	0.24	0.24
Component Flowrates (kg/batch)				
Argon	1,433.90	0.14	3.23	32.41
Carb. Dioxide	62.34	0.01	0.14	1.41
Nitrogen	121,709.92	11.85	274.40	2,750.73
Oxygen	32,652.36	3.18	73.62	737.97
TOTAL (kg/batch)	155,858.52	15.17	351.39	3,522.51
TOTAL (L/batch)	23,392,056.23	2,276.99	52,738.97	528,676.60

Stream Name	S-143	Base to SFR-1	S-133	Inoculum to SFR-2
**Source**	**P-41**	**INPUT**	**P-16**	**P-16**
**Destination**	**P-4**	**P-16**	**P-32**	**P-1**
Stream Properties				
Activity (U/ml)	0.00	0.00	0.00	0.00
Temperature (°C)	40.00	25.00	35.00	34.92
Pressure (bar)	6.01	1.01	1.01	1.06
Density (g/L)	6.66	2,329.54	1.20	1,009.48
Total Enthalpy (kW-h)	1,710.53	0.01	0.33	1.30
Specific Enthalpy (kcal/kg)	9.68	7.12	15.98	34.34
Heat Capacity (kcal/kg-°C)	0.24	0.28	0.24	0.98
Component Flowrates (kg/batch)				
Amm. Sulfate	0.00	0.00	0.00	0.00
Argon	1,398.12	0.00	0.14	0.00
Biomass	0.00	0.00	0.00	1.62
Ca Hydroxide	0.00	0.88	0.00	0.88
Carb. Dioxide	60.79	0.00	2.52	0.00
NaH2PO4	0.00	0.00	0.00	0.00
Nitrogen	118,672.94	0.00	11.88	0.00
Oxygen	31,837.60	0.00	3.19	0.00
Sucrose	0.00	0.00	0.00	0.00
Water	0.00	0.00	0.00	30.14
TOTAL (kg/batch)	151,969.45	0.88	17.72	32.65
TOTAL (L/batch)	22,808,363.68	0.38	14,776.23	32.34

Stream Name	Vent SFR-1	S-131	Vent SFR-2	S-119
**Source**	**P-32**	**P-1**	**P-29**	**P-15**
**Destination**	**OUTPUT**	**P-29**	**OUTPUT**	**P-28**
Stream Properties				
Activity (U/ml)	0.00	0.00	0.00	0.00
Temperature (°C)	35.00	35.00	35.00	35.00
Pressure (bar)	1.01	1.01	1.01	1.01
Density (g/L)	1.20	1.18	1.18	1.18
Total Enthalpy (kW-h)	0.33	6.44	6.44	63.84
Specific Enthalpy (kcal/kg)	15.98	14.05	14.05	13.94
Heat Capacity (kcal/kg-°C)	0.24	0.24	0.24	0.24
Component Flowrates (kg/batch)				
Argon	0.14	3.24	3.24	32.50
Carb. Dioxide	2.52	41.72	41.72	408.90
Nitrogen	11.88	275.17	275.17	2,758.35
Oxygen	3.19	73.82	73.82	740.01
TOTAL (kg/batch)	17.72	393.95	393.95	3,939.75
TOTAL (L/batch)	14,776.23	332,731.50	332,731.50	3,330,069.70

Stream Name	Vent SFR-3	Vent FR-1	Emissions	S-117
**Source**	**P-28**	**P-4**	**P-49**	**P-26**
**Destination**	**OUTPUT**	**P-49**	**OUTPUT**	**P-30**
Stream Properties				
Activity (U/ml)	0.00	0.00	0.00	0.00
Temperature (°C)	35.00	35.00	35.00	35.31
Pressure (bar)	1.01	1.01	1.01	1.01
Density (g/L)	1.18	1.20	1.20	995.45
Total Enthalpy (kW-h)	63.84	3,145.45	3,145.45	4,979.44
Specific Enthalpy (kcal/kg)	13.94	15.44	15.44	35.14
Heat Capacity (kcal/kg-°C)	0.24	0.24	0.24	0.99
Component Flowrates (kg/batch)				
Amm. Sulfate	0.00	0.00	0.00	0.94
Ammonium Chlori	0.00	0.00	0.00	37.33
Argon	32.50	1,399.87	1,399.87	0.00
Ca Hydroxide	0.00	0.00	0.00	2.10
Carb. Dioxide	408.90	23,157.93	23,157.93	0.00
NaH2PO4	0.00	0.00	0.00	10.11
Nitrogen	2,758.35	118,821.88	118,821.88	0.00
Oxygen	740.01	31,877.56	31,877.56	0.00
pHBA Salt	0.00	0.00	0.00	403.39
Sucrose	0.00	0.00	0.00	765.54
Water	0.00	0.00	0.00	120,693.13
TOTAL (kg/batch)	3,939.75	175,257.24	175,257.24	121,912.53
TOTAL (L/batch)	3,330,069.70	146,635,142.97	146,635,142.97	122,469.77

Stream Name	Purge	S-111	Nitric Acid 70%	S-103
**Source**	**P-30**	**P-30**	**INPUT**	**P-31**
**Destination**	**OUTPUT**	**P-17**	**P-3**	**P-3**
Stream Properties				
Activity (U/ml)	0.00	0.00	0.00	0.00
Temperature (°C)	35.31	35.31	25.00	35.31
Pressure (bar)	1.01	1.01	1.01	1.01
Density (g/L)	995.45	995.45	1,355.32	1,192.31
Total Enthalpy (kW-h)	2,811.45	2,167.99	186.35	2,325.31
Specific Enthalpy (kcal/kg)	35.14	35.14	14.83	27.50
Heat Capacity (kcal/kg-°C)	0.99	0.99	0.59	0.78
Component Flowrates (kg/batch)				
Amm. Sulfate	0.53	0.41	0.00	0.42
Ammonium Chlori	21.08	16.25	0.00	16.78
Ca Hydroxide	1.19	0.92	0.00	0.94
NaH2PO4	5.71	4.40	0.00	4.54
Nitric Acid	0.00	0.00	7,566.35	0.00
pHBA Salt	227.76	175.63	0.00	18,144.18
Sucrose	432.23	333.31	0.00	344.12
Water	68,144.79	52,548.34	3,242.72	54,252.69
TOTAL (kg/batch)	68,833.28	53,079.25	10,809.07	72,763.68
TOTAL (L/batch)	69,147.90	53,321.87	7,975.29	61,027.33

Stream Name	S-102	S-104	Base to SFR-2	Inoculum to SFR-3
**Source**	**P-3**	**P-13**	**INPUT**	**P-1**
**Destination**	**P-13**	**P-11**	**P-1**	**P-15**
Stream Properties				
Activity (U/ml)	0.00	0.00	0.00	0.00
Temperature (°C)	33.67	5.00	25.00	34.92
Pressure (bar)	1.01	1.01	1.01	1.06
Density (g/L)	1,114.42	1,128.18	2,329.54	1,008.87
Total Enthalpy (kW-h)	2,517.77	375.77	0.18	32.84
Specific Enthalpy (kcal/kg)	25.92	3.87	7.12	34.32
Heat Capacity (kcal/kg-°C)	0.77	0.77	0.28	0.98
Component Flowrates (kg/batch)				
Amm. Sulfate	0.42	0.42	0.00	0.00
Ammonium Chlori	16.78	16.78	0.00	0.00
Biomass	0.00	0.00	0.00	24.77
Ca Hydroxide	0.94	0.94	22.12	23.01
Calcium Nitrate	9,472.73	9,472.73	0.00	0.00
NaH2PO4	4.54	4.54	0.00	0.00
Nitric Acid	291.01	291.01	0.00	0.00
pHBA (aq)	15,947.14	374.96	0.00	0.00
pHBA (solid)	0.00	15,572.18	0.00	0.00
Sucrose	344.12	344.12	0.00	0.03
Water	57,495.41	57,495.41	0.00	775.49
TOTAL (kg/batch)	83,573.10	83,573.10	22.12	823.30
TOTAL (L/batch)	74,992.19	74,077.89	9.50	816.07

Stream Name	Base to SFR-3	Inoculum to FR-1	Base to FR-1	S-105
**Source**	**INPUT**	**P-15**	**INPUT**	**P-4**
**Destination**	**P-15**	**P-4**	**P-4**	**P-19**
Stream Properties				
Activity (U/ml)	0.00	0.00	0.00	0.00
Temperature (°C)	25.00	34.92	25.00	35.00
Pressure (bar)	1.01	1.06	1.01	1.01
Density (g/L)	2,329.54	1,009.86	2,329.54	1,073.42
Total Enthalpy (kW-h)	1.83	328.15	33.93	6,186.97
Specific Enthalpy (kcal/kg)	7.12	34.28	7.12	31.58
Heat Capacity (kcal/kg-°C)	0.28	0.98	0.28	0.90
Component Flowrates (kg/batch)				
Amm. Sulfate	0.00	0.00	0.00	1.04
Ammonium Chlori	0.00	0.00	0.00	41.12
Biomass	0.00	249.42	0.00	4,886.37
Ca Hydroxide	221.21	244.22	4,100.75	2.32
NaH2PO4	0.00	0.00	0.00	11.13
pHBA Salt	0.00	0.00	0.00	18,420.90
Sucrose	0.00	0.03	0.00	843.27
Water	0.00	7,743.82	0.00	144,350.00
TOTAL (kg/batch)	221.21	8,237.50	4,100.75	168,556.15
TOTAL (L/batch)	94.96	8,157.07	1,760.33	157,027.26

Stream Name	S-113	RVF Cake	S-107	S-126
**Source**	**P-19**	**P-17**	**P-17**	**P-26**
**Destination**	**P-17**	**OUTPUT**	**P-26**	**P-31**
Stream Properties				
Activity (U/ml)	0.00	0.00	0.00	0.00
Temperature (°C)	35.00	35.12	35.07	35.31
Pressure (bar)	10.47	1.01	1.01	1.01
Density (g/L)	1,073.42	1,003.01	1,061.01	1,191.83
Total Enthalpy (kW-h)	6,187.01	1,090.76	7,264.24	2,333.78
Specific Enthalpy (kcal/kg)	31.58	35.10	32.07	27.51
Heat Capacity (kcal/kg-°C)	0.90	1.00	0.91	0.78
Component Flowrates (kg/batch)				
Amm. Sulfate	1.04	0.08	1.36	0.42
Ammonium Chlori	41.12	3.25	54.12	16.79
Biomass	4,886.37	4,837.51	48.86	48.86
Ca Hydroxide	2.32	0.18	3.05	0.95
NaH2PO4	11.13	0.88	14.65	4.55
pHBA Salt	18,420.90	35.13	18,561.41	18,158.02
Sucrose	843.27	66.66	1,109.92	344.38
Water	144,350.00	21,797.13	175,101.20	54,408.07
TOTAL (kg/batch)	168,556.15	26,740.82	194,894.58	72,982.04
TOTAL (L/batch)	157,027.27	26,660.61	183,688.54	61,235.46

Stream Name	DEF Cake	Water for Cake Wash	Wastewater	S-101
**Source**	**P-31**	**INPUT**	**P-11**	**P-11**
**Destination**	**OUTPUT**	**P-11**	**OUTPUT**	**P-14**
Stream Properties				
Activity (U/ml)	0.00	0.00	0.00	0.00
Temperature (°C)	35.31	25.00	11.90	22.88
Pressure (bar)	1.01	1.01	1.01	1.88
Density (g/L)	1,049.17	994.70	1,058.55	1,217.13
Total Enthalpy (kW-h)	8.47	1,004.83	1,232.32	268.82
Specific Enthalpy (kcal/kg)	33.37	25.11	10.96	10.89
Heat Capacity (kcal/kg-°C)	0.94	1.00	0.92	0.47
Component Flowrates (kg/batch)				
Amm. Sulfate	0.00	0.00	0.42	0.00
Ammonium Chlori	0.01	0.00	16.78	0.00
Biomass	48.86	0.00	0.00	0.00
Ca Hydroxide	0.00	0.00	0.94	0.00
Calcium Nitrate	0.00	0.00	9,472.73	0.00
NaH2PO4	0.00	0.00	4.54	0.00
Nitric Acid	0.00	0.00	291.01	0.00
pHBA (aq)	0.00	0.00	374.96	0.00
pHBA (solid)	0.00	0.00	311.44	15,260.73
pHBA Salt	13.84	0.00	0.00	0.00
Sucrose	0.26	0.00	344.12	0.00
Water	155.39	34,433.02	85,943.67	5,984.76
TOTAL (kg/batch)	218.37	34,433.02	96,760.62	21,245.50
TOTAL (L/batch)	208.13	34,616.33	91,408.69	17,455.46

Stream Name	Humid Air	Final Product
**Source**	**P-14**	**P-14**
**Destination**	**OUTPUT**	**OUTPUT**
Stream Properties		
Activity (U/ml)	0.00	0.00
Temperature (°C)	50.00	50.00
Pressure (bar)	1.01	1.01
Density (g/L)	1.08	1,303.70
Total Enthalpy (kW-h)	8,503.62	242.63
Specific Enthalpy (kcal/kg)	24.28	13.61
Heat Capacity (kcal/kg-°C)	0.25	0.27
Component Flowrates (kg/batch)		
Amm. Sulfate	0.00	0.00
Ammonium Chlori	0.00	0.00
Argon	2,717.71	0.00
Ca Hydroxide	0.00	0.00
Calcium Nitrate	0.00	0.00
Carb. Dioxide	118.16	0.00
NaH2PO4	0.00	0.00
Nitric Acid	0.00	0.00
Nitrogen	230,680.73	0.00
Oxygen	61,887.07	0.00
pHBA (aq)	0.00	0.00
pHBA (solid)	0.00	15,260.73
Sucrose	0.00	0.00
Water	5,908.07	76.69
TOTAL (kg/batch)	301,311.75	15,337.42
TOTAL (L/batch)	280,213,901.78	11,764.51

4. OVERALL COMPONENT BALANCE (kg/batch)

COMPONENT	INITIAL	INPUT	OUTPUT	FINAL	IN-OUT
Amm. Sulfate	0.00	54.67	1.04	0.00	53.63
Ammonium Chlori	0.00	2,168.60	41.12	0.00	2,127.48
Argon	4.55	4,151.61	4,153.47	2.70	0.00
Biomass	0.00	0.00	4,886.37	0.00	- 4,886.37
Ca Hydroxide	0.00	4,344.97	2.32	0.00	4,342.66
Calcium Nitrate	0.00	0.00	9,472.73	0.00	- 9,472.73
Carb. Dioxide	0.20	180.50	23,729.23	6.59	- 23,555.12
NaH2PO4	0.00	587.27	11.13	0.00	576.14
Nitric Acid	0.00	7,566.35	291.01	0.00	7,275.34
Nitrogen	386.34	352,390.65	352,548.00	228.99	0.00
Oxygen	103.65	94,539.43	94,581.64	61.43	0.00
pHBA (aq)	0.00	0.00	374.96	0.00	- 374.96
pHBA (solid)	0.00	0.00	15,572.18	0.00	- 15,572.18
pHBA Salt	0.00	0.00	276.72	0.00	- 276.72
Phosphoric Acid	0.00	180.99	180.99	0.00	0.00
Sodium Hydroxid	0.00	245.19	245.19	0.00	0.00
Sucrose	0.00	42,717.86	843.27	0.00	41,874.59
Water	0.00	263,931.92	266,043.61	0.00	- 2,111.69
TOTAL	494.73	773,060.02	773,254.98	299.71	0.06

5. EQUIPMENT CONTENTS

SFR-3

Procedure	Operation	Time (in h)	Volume (in L)	Vapor (in kg)
P-15	START	25.61	0.00	12.02(*)
P-15	TRANSFER-IN-SALTS (Transfer In)	26.61	2,448.82	12.02(*)
P-15	TRANSFER-IN-INITIAL-SUGAR (Transfer In)	27.61	6,886.68	12.02(*)
P-15	TRANSFER-IN-INOCULUM (Transfer In)	28.11	7,702.75	12.02(*)
P-15	FERMENT-2 (Batch Stoich. Fermentation)	40.11	8,061.24	2.53(*)
P-15	CHARGE-1 (Charge)	40.11	8,157.07	2.53(*)
P-15	TRANSFER-OUT-1 (Transfer Out)	41.11	0.00	2.53(*)
P-15	CIP-1 (In-Place-Cleaning)	43.19	0.00	2.53(*)
P-15	SIP-1 (In-Place-Steaming)	45.19	0.00	2.53(*)

(*) Contains material in vapor phase other than Oxygen & Nitrogen

SFR-2

Procedure	Operation	Time (in h)	Volume (in L)	Vapor (in kg)
P-1	START	14.11	0.00	1.20(*)
P-1	TRANSFER-IN-SALTS (Transfer In)	14.61	244.86	1.20(*)
P-1	TRANSFER-IN-INITIAL-SUGAR (Transfer In)	15.11	736.05	1.20(*)
P-1	AGITATE-1 (Agitation)	15.11	736.06	1.20(*)
P-1	TRANSFER-IN-INOCULUM (Transfer In)	15.61	768.40	1.20(*)
P-1	FERMENT-1 (Batch Stoich. Fermentation)	27.61	806.48	0.25(*)
P-1	CHARGE-1 (Charge)	27.61	816.07	0.25(*)
P-1	TRANSFER-OUT-1 (Transfer Out)	28.11	0.00	0.25(*)
P-1	CIP-1 (In-Place-Cleaning)	30.19	0.00	0.25(*)
P-1	SIP-1 (In-Place-Steaming)	31.19	0.00	0.25(*)

(*) Contains material in vapor phase other than Oxygen & Nitrogen

SFR-1

Procedure	Operation	Time (in h)	Volume (in L)	Vapor (in kg)
P-16	START	0.00	0.00	0.05(*)
P-16	TRANSFER-IN-PHOSPHATE (Transfer In)	0.25	3.28	0.05(*)
P-16	TRANSFER-IN-SULFATE (Transfer In)	0.50	6.62	0.05(*)
P-16	TRANSFER-IN-NH4Cl (Transfer In)	0.75	9.82	0.05(*)
P-16	TRANSFER-IN-INITIAL-SUGAR (Transfer In)	1.00	28.21	0.05(*)
P-16	FERMENT (Batch Stoich. Fermentation)	15.11	31.96	0.01(*)
P-16	CHARGE-1 (Charge)	15.11	32.34	0.01(*)
P-16	TRANSFER-OUT (Transfer Out)	15.61	0.00	0.01(*)
P-16	CIP-1 (In-Place-Cleaning)	17.69	0.00	0.01(*)
P-16	SIP-1 (In-Place-Steaming)	18.19	0.00	0.01(*)

(*) Contains material in vapor phase other than Oxygen & Nitrogen

BCFBD-101

Procedure	Operation	Time (in h)	Volume (in L)	Vapor (in kg)
P-11	START	89.44	0.00	11.07(*)
P-11	FILTER-1 (Cloth Filtration)	100.94	4,327.04	11.07(*)
P-11	CAKE-WASH-1 (Cake Wash)	101.19	4,363.87	11.07(*)
P-11	TRANSFER-OUT-1 (Transfer Out)	101.44	0.00	11.07(*)

(*) Contains material in vapor phase other than Oxygen & Nitrogen

V-102

Procedure	Operation	Time (in h)	Volume (in L)	Vapor (in kg)
P-19	START	76.27	0.00	205.74(*)
P-19	TRANSFER-IN-1 (Transfer In)	78.27	157,027.27	205.74(*)
P-19	TRANSFER-OUT-1 (Transfer Out)	100.27	0.00	205.74(*)

(*) Contains material in vapor phase other than Oxygen & Nitrogen

DE-101

Procedure	Operation	Time (in h)	Volume (in L)	Vapor (in kg)
P-31	START	78.44	0.00	0.00
P-31	FILTER-1 (Dead-End Filtration)	89.44	104.07	0.00
P-31	TRANSFER-OUT-1 (Transfer Out)	90.44	0.00	0.00

FR-1

Procedure	Operation	Time (in h)	Volume (in L)	Vapor (in kg)
P-4	START	39.11	0.00	231.46(*)
P-4	TRANSFER-IN-SULFATE (Transfer In)	40.11	16,646.67	231.46(*)
P-4	TRANSFER-IN-NH4Cl (Transfer In)	40.11	32,595.75	231.46(*)
P-4	TRANSFER-IN-PHOSPHATE (Transfer In)	40.11	48,976.85	231.46(*)
P-4	TRANSFER-IN-INITIAL-SUGAR (Transfer In)	40.11	100,348.14	231.46(*)
P-4	TRANSFER-IN-INOCULUM (Transfer In)	41.11	108,505.13	231.46(*)
P-4	CHARGE-1 (Charge)	76.27	110,281.46	231.46(*)
P-4	FERMENT-1 (Batch Stoich. Fermentation)	76.27	157,027.26	46.92(*)
P-4	TRANSFER-OUT-1 (Transfer Out)	78.27	0.00	46.92(*)
P-4	CIP-1 (In-Place-Cleaning)	80.35	0.00	46.92(*)
P-4	SIP-1 (In-Place-Steaming)	82.35	0.00	46.92(*)

(*) Contains material in vapor phase other than Oxygen & Nitrogen
