# Supplementary material for: Economic Process Evaluation and Environmental Life-Cycle Assessment of Bio-Aromatics Production
Source: Front Bioeng Biotechnol. 2020 May 13;8:403. doi: 10.3389/fbioe.2020.00403 (PMC7237583; doi:10.3389/fbioe.2020.00403)
Supplement: Supplementary file 1 [file Data_Sheet_1.zip › Sc_8.pdf]

Materials & Streams Report

for Supplementary_8_yeast_base_case

März 20, 2020

1. OVERALL PROCESS DATA

Annual Operating Time	7,918.35 h
Unit Production Ref. Rate	10,000,029.03 kg MP/yr
Batch Size	15,290.56 kg MP
Recipe Batch Time	82.35 h
Recipe Cycle Time	12.00 h
Number of Batches per Year	654.00

MP = Total Flow of Stream 'Final Product'

2.1 STARTING MATERIAL REQUIREMENTS (per Section)

Section	Starting Material	Active Product	Amount Needed (kg Sin/kg MP)	Molar Yield (%)	Mass Yield (%)	Gross Mass Yield (%)
Fermentation Section	(none)	(none)	0.00	Unknown	Unknown	Unknown
Downstream Section	(none)	(none)	0.00	Unknown	Unknown	Unknown

Sin = Section Starting Material, Aout = Section Active Product

2.2 BULK MATERIALS (Entire Process)

Material	kg/yr	kg/batch	kg/kg MP
Air	312,774,662	478,248.72	31.28
Amm. Sulfate	37,220	56.91	0.00
Ammonium Chlori	1,476,523	2,257.68	0.15
H3PO4 (2%)	5,946,968	9,093.22	0.59
NaH2PO4	399,849	611.39	0.04
NaOH (0.5 M)	8,196,117	12,532.29	0.82
Sucrose	29,085,048	44,472.55	2.91
Water	129,863,254	198,567.67	12.99
TOTAL	487,779,640	745,840.43	48.78

2.3 BULK MATERIALS (per Section)

SECTIONS IN: Main Branch

Fermentation Section

Material	kg/yr	kg/batch	kg/kg MP
Air	104,349,407	159,555.67	10.43
Amm. Sulfate	37,220	56.91	0.00
Ammonium Chlori	1,476,523	2,257.68	0.15
H3PO4 (2%)	5,946,968	9,093.22	0.59
NaH2PO4	399,849	611.39	0.04
NaOH (0.5 M)	8,196,117	12,532.29	0.82
Sucrose	29,085,048	44,472.55	2.91
Water	106,780,021	163,272.20	10.68
TOTAL	256,271,152	391,851.91	25.63

Downstream Section			
**Material**	**kg/yr**	**kg/batch**	**kg/kg MP**
Air	208,425,255	318,693.05	20.84
Water	23,083,233	35,295.46	2.31
TOTAL	231,508,488	353,988.51	23.15

2.4 BULK MATERIALS (per Material)

Air				
**Procedure**	**% Total**	**kg/yr**	**kg/batch**	**kg/kg MP**
Fermentation Section (Main Branch)				
P-51	33.36	104,349,407	159,555.67	10.43
Downstream Section (Main Branch)				
P-3	66.64	208,425,255	318,693.05	20.84
TOTAL	100.00	312,774,662	478,248.72	31.28

Amm. Sulfate				
**Procedure**	**% Total**	**kg/yr**	**kg/batch**	**kg/kg MP**
Fermentation Section (Main Branch)				
P-36	100.00	37,220	56.91	0.00
TOTAL	100.00	37,220	56.91	0.00

Ammonium Chlori				
**Procedure**	**% Total**	**kg/yr**	**kg/batch**	**kg/kg MP**
Fermentation Section (Main Branch)				
P-38	100.00	1,476,523	2,257.68	0.15
TOTAL	100.00	1,476,523	2,257.68	0.15

H3PO4 (2%)				
**Procedure**	**% Total**	**kg/yr**	**kg/batch**	**kg/kg MP**
Fermentation Section (Main Branch)				
P-4	45.81	2,724,173	4,165.40	0.27
P-1	8.51	506,254	774.09	0.05
P-15	42.78	2,543,921	3,889.79	0.25
P-16	2.90	172,621	263.95	0.02
TOTAL	100.00	5,946,968	9,093.22	0.59

NaH2PO4				
**Procedure**	**% Total**	**kg/yr**	**kg/batch**	**kg/kg MP**
Fermentation Section (Main Branch)				
P-34	100.00	399,849	611.39	0.04
TOTAL	100.00	399,849	611.39	0.04

NaOH (0.5 M)				
**Procedure**	**% Total**	**kg/yr**	**kg/batch**	**kg/kg MP**
Fermentation Section (Main Branch)				
P-4	78.23	6,411,622	9,803.70	0.64
P-1	6.23	510,652	780.81	0.05
P-15	13.42	1,099,723	1,681.53	0.11
P-16	2.12	174,120	266.24	0.02
TOTAL	100.00	8,196,117	12,532.29	0.82

Sucrose				
**Procedure**	**% Total**	**kg/yr**	**kg/batch**	**kg/kg MP**
Fermentation Section (Main Branch)				
P-9	100.00	29,085,048	44,472.55	2.91
TOTAL	100.00	29,085,048	44,472.55	2.91

Water				
**Procedure**	**% Total**	**kg/yr**	**kg/batch**	**kg/kg MP**
Fermentation Section (Main Branch)				
P-4	4.64	6,023,403	9,210.10	0.60
P-34	8.83	11,469,447	17,537.38	1.15
P-36	9.11	11,832,121	18,091.93	1.18
P-38	8.00	10,392,773	15,891.09	1.04
P-9	22.40	29,085,048	44,472.55	2.91
P-18	0.01	11,665	17.84	0.00
P-21	2.16	2,808,013	4,293.60	0.28
P-23	0.24	312,788	478.27	0.03
P-25	23.82	30,933,059	47,298.26	3.09
P-1	0.86	1,119,375	1,711.58	0.11
P-15	1.86	2,410,649	3,686.01	0.24
P-16	0.29	381,680	583.61	0.04
Downstream Section (Main Branch)				
P-11	17.78	23,083,233	35,295.46	2.31
TOTAL	100.00	129,863,254	198,567.67	12.99

2.5 BULK MATERIALS: SECTION TOTALS (kg/kg MP)

Raw Material	Fermentation Section	Downstream Section
Air	10.43	20.84
Amm. Sulfate	0.00	0.00
Ammonium Chlori	0.15	0.00
H3PO4 (2%)	0.59	0.00
NaH2PO4	0.04	0.00
NaOH (0.5 M)	0.82	0.00
Sucrose	2.91	0.00
Water	10.68	2.31
TOTAL	25.63	23.15

2.6 BULK MATERIALS: SECTION TOTALS (kg/batch)

Raw Material	Fermentation Section	Downstream Section
Air	159,555.67	318,693.05
Amm. Sulfate	56.91	0.00
Ammonium Chlori	2,257.68	0.00
H3PO4 (2%)	9,093.22	0.00
NaH2PO4	611.39	0.00
NaOH (0.5 M)	12,532.29	0.00
Sucrose	44,472.55	0.00
Water	163,272.20	35,295.46
TOTAL	391,851.91	353,988.51

2.7 BULK MATERIALS: SECTION TOTALS (kg/yr)

Raw Material	Fermentation Section	Downstream Section
Air	104,349,407	208,425,255
Amm. Sulfate	37,220	0
Ammonium Chlori	1,476,523	0
H3PO4 (2%)	5,946,968	0
NaH2PO4	399,849	0
NaOH (0.5 M)	8,196,117	0
Sucrose	29,085,048	0
Water	106,780,021	23,083,233
TOTAL	256,271,152	231,508,488

3. STREAM DETAILS

Stream Name	Air for Drying	S-104	Water for NH4Cl	NH4Cl
**Source**	**INPUT**	**P-3**	**INPUT**	**INPUT**
**Destination**	**P-3**	**P-14**	**P-38**	**P-38**
Stream Properties				
Activity (U/ml)	0.00	0.00	0.00	0.00
Temperature (°C)	25.00	37.66	10.00	20.00
Pressure (bar)	1.01	1.21	1.01	1.01
Density (g/L)	1.18	1.35	1,000.17	1,519.00
Total Enthalpy (kW-h)	2,244.53	3,377.24	186.07	19.72
Specific Enthalpy (kcal/kg)	6.06	9.12	10.07	7.52
Heat Capacity (kcal/kg-°C)	0.24	0.24	1.01	0.38
Component Flowrates (kg/batch)				
Ammonium Chlori	0.00	0.00	0.00	2,257.68
Argon	2,931.98	2,931.98	0.00	0.00
Carb. Dioxide	127.48	127.48	0.00	0.00
Nitrogen	248,867.40	248,867.40	0.00	0.00
Oxygen	66,766.19	66,766.19	0.00	0.00
Water	0.00	0.00	15,891.09	0.00
TOTAL (kg/batch)	318,693.05	318,693.05	15,891.09	2,257.68
TOTAL (L/batch)	270,262,312.00	235,291,223.49	15,888.36	1,486.29

Stream Name	Cl-Solution	S-129	NH4Cl to SFR-1	NH4Cl to SFR-2
**Source**	**P-38**	**P-37**	**P-5**	**P-5**
**Destination**	**P-37**	**P-5**	**P-16**	**P-64**
Stream Properties				
Activity (U/ml)	0.00	0.00	0.00	0.00
Temperature (°C)	10.50	35.00	35.00	35.00
Pressure (bar)	1.01	1.01	1.01	1.01
Density (g/L)	1,044.38	1,035.84	1,035.84	1,035.84
Total Enthalpy (kW-h)	205.79	682.80	0.13	3.24
Specific Enthalpy (kcal/kg)	9.76	32.37	32.37	32.37
Heat Capacity (kcal/kg-°C)	0.93	0.92	0.92	0.92
Component Flowrates (kg/batch)				
Ammonium Chlori	2,257.68	2,257.68	0.43	10.70
Water	15,891.09	15,891.09	3.02	75.29
TOTAL (kg/batch)	18,148.77	18,148.77	3.45	85.99
TOTAL (L/batch)	17,377.57	17,520.74	3.33	83.01

Stream Name	NH4Cl to SFR-3	NH4Cl to FR-1	Water for NH4SO4	NH4SO4
**Source**	**P-5**	**P-5**	**INPUT**	**INPUT**
**Destination**	**P-65**	**P-4**	**P-36**	**P-36**
Stream Properties				
Activity (U/ml)	0.00	0.00	0.00	0.00
Temperature (°C)	35.00	35.00	10.00	20.00
Pressure (bar)	1.01	1.01	1.01	1.01
Density (g/L)	1,035.84	1,035.84	1,000.17	1,769.00
Total Enthalpy (kW-h)	32.35	647.08	211.84	0.45
Specific Enthalpy (kcal/kg)	32.37	32.37	10.07	6.80
Heat Capacity (kcal/kg-°C)	0.92	0.92	1.01	0.34
Component Flowrates (kg/batch)				
Amm. Sulfate	0.00	0.00	0.00	56.91
Ammonium Chlori	106.98	2,139.58	0.00	0.00
Water	752.98	15,059.80	18,091.93	0.00
TOTAL (kg/batch)	859.96	17,199.37	18,091.93	56.91
TOTAL (L/batch)	830.20	16,604.20	18,088.82	32.17

Stream Name	SO4-Solution	S-138	Sulfate to SFR-1	Sulfate to SFR-2
**Source**	**P-36**	**P-35**	**P-6**	**P-6**
**Destination**	**P-35**	**P-6**	**P-16**	**P-64**
Stream Properties				
Activity (U/ml)	0.00	0.00	0.00	0.00
Temperature (°C)	10.01	35.00	35.00	35.00
Pressure (bar)	1.01	1.01	1.01	1.01
Density (g/L)	1,001.53	992.43	992.43	992.43
Total Enthalpy (kW-h)	212.29	738.86	0.14	3.50
Specific Enthalpy (kcal/kg)	10.06	35.03	35.03	35.03
Heat Capacity (kcal/kg-°C)	1.00	1.00	1.00	1.00
Component Flowrates (kg/batch)				
Amm. Sulfate	56.91	56.91	0.01	0.27
Water	18,091.93	18,091.93	3.44	85.72
TOTAL (kg/batch)	18,148.84	18,148.84	3.45	85.99
TOTAL (L/batch)	18,121.06	18,287.31	3.47	86.65

Stream Name	Sulfate to SFR-3	Sulfate to FR-1	Water for NaH2PO4	NaH2PO4
**Source**	**P-6**	**P-6**	**INPUT**	**INPUT**
**Destination**	**P-65**	**P-4**	**P-34**	**P-34**
Stream Properties				
Activity (U/ml)	0.00	0.00	0.00	0.00
Temperature (°C)	35.00	35.00	10.00	20.00
Pressure (bar)	1.01	1.01	1.01	1.01
Density (g/L)	992.43	992.43	1,000.17	2,040.00
Total Enthalpy (kW-h)	35.01	700.21	205.35	2.13
Specific Enthalpy (kcal/kg)	35.03	35.03	10.07	3.00
Heat Capacity (kcal/kg-°C)	1.00	1.00	1.01	0.15
Component Flowrates (kg/batch)				
Amm. Sulfate	2.70	53.93	0.00	0.00
NaH2PO4	0.00	0.00	0.00	611.39
Water	857.27	17,145.50	17,537.38	0.00
TOTAL (kg/batch)	859.96	17,199.44	17,537.38	611.39
TOTAL (L/batch)	866.53	17,330.67	17,534.37	299.70

Stream Name	PO4-Solution	S-108	Phosphate to SFR-1	Phosphate to SFR-2
**Source**	**P-34**	**P-33**	**P-2**	**P-2**
**Destination**	**P-33**	**P-2**	**P-16**	**P-64**
Stream Properties				
Activity (U/ml)	0.00	0.00	0.00	0.00
Temperature (°C)	10.05	35.00	35.00	35.00
Pressure (bar)	1.01	1.01	1.01	1.01
Density (g/L)	1,017.63	1,008.53	1,008.53	1,008.53
Total Enthalpy (kW-h)	207.48	719.18	0.14	3.41
Specific Enthalpy (kcal/kg)	9.84	34.10	34.10	34.10
Heat Capacity (kcal/kg-°C)	0.98	0.97	0.97	0.97
Component Flowrates (kg/batch)				
NaH2PO4	611.39	611.39	0.12	2.90
Water	17,537.38	17,537.38	3.33	83.09
TOTAL (kg/batch)	18,148.77	18,148.77	3.45	85.99
TOTAL (L/batch)	17,834.40	17,995.29	3.42	85.26

Stream Name	Phosphate to SFR-3	Phosphate to FR-1	Salts to SFR-3	Salts to SFR-2
**Source**	**P-2**	**P-2**	**P-65**	**P-64**
**Destination**	**P-65**	**P-4**	**P-15**	**P-1**
Stream Properties				
Activity (U/ml)	0.00	0.00	0.00	0.00
Temperature (°C)	35.00	35.00	35.00	35.00
Pressure (bar)	1.01	1.01	1.01	1.01
Density (g/L)	1,008.53	1,008.53	1,011.95	1,011.95
Total Enthalpy (kW-h)	34.08	681.56	101.44	10.14
Specific Enthalpy (kcal/kg)	34.10	34.10	33.83	33.83
Heat Capacity (kcal/kg-°C)	0.97	0.97	0.96	0.96
Component Flowrates (kg/batch)				
Amm. Sulfate	0.00	0.00	2.70	0.27
Ammonium Chlori	0.00	0.00	106.98	10.70
NaH2PO4	28.97	579.41	28.97	2.90
Water	830.99	16,619.96	2,441.24	244.10
TOTAL (kg/batch)	859.96	17,199.37	2,579.89	257.97
TOTAL (L/batch)	852.69	17,053.92	2,549.42	254.92

Stream Name	S-123	S-125	S-112	S-118
**Source**	**INPUT**	**P-25**	**INPUT**	**P-21**
**Destination**	**P-25**	**P-24**	**P-21**	**P-20**
Stream Properties				
Activity (U/ml)	0.00	0.00	0.00	0.00
Temperature (°C)	25.00	35.00	25.00	35.00
Pressure (bar)	1.01	1.01	1.01	1.01
Density (g/L)	994.70	991.06	994.70	991.06
Total Enthalpy (kW-h)	1,380.26	1,929.57	125.30	175.16
Specific Enthalpy (kcal/kg)	25.11	35.10	25.11	35.10
Heat Capacity (kcal/kg-°C)	1.00	1.00	1.00	1.00
Component Flowrates (kg/batch)				
Water	47,298.26	47,298.26	4,293.60	4,293.60
TOTAL (kg/batch)	47,298.26	47,298.26	4,293.60	4,293.60
TOTAL (L/batch)	47,550.06	47,724.95	4,316.46	4,332.33

Stream Name	S-120	S-122	Water for 50% Sucrose	Process Sucrose
Source	INPUT	P-23	INPUT	INPUT
Destination	P-23	P-22	P-9	P-9
Stream Properties				
Activity (U/ml)	0.00	0.00	0.00	0.00
Temperature (°C)	25.00	35.00	25.00	25.00
Pressure (bar)	1.01	1.01	1.01	1.01
Density (g/L)	994.70	991.06	994.70	1,509.84
Total Enthalpy (kW-h)	13.96	19.51	1,297.80	386.97
Specific Enthalpy (kcal/kg)	25.11	35.10	25.11	7.49
Heat Capacity (kcal/kg-°C)	1.00	1.00	1.00	0.30
Component Flowrates (kg/batch)				
Sucrose	0.00	0.00	0.00	44,472.55
Water	478.27	478.27	44,472.55	0.00
TOTAL (kg/batch)	478.27	478.27	44,472.55	44,472.55
TOTAL (L/batch)	480.82	482.58	44,709.32	29,455.21

Stream Name	S-144	S-106	Batch Sucrose	Fed-Batch Sucrose
Source	P-9	P-8	Sucrose Storage	Sucrose Storage
Destination	P-8	Sucrose Storage	P-7	P-10
Stream Properties				
Activity (U/ml)	0.00	0.00	0.00	0.00
Temperature (°C)	25.00	35.00	35.00	35.00
Pressure (bar)	1.01	1.01	1.01	1.01
Density (g/L)	1,199.29	1,195.13	1,195.13	1,195.13
Total Enthalpy (kW-h)	1,684.77	2,356.04	192.30	2,163.75
Specific Enthalpy (kcal/kg)	16.30	22.79	22.79	22.79
Heat Capacity (kcal/kg-°C)	0.65	0.65	0.65	0.65
Component Flowrates (kg/batch)				
Sucrose	44,472.55	44,472.55	3,629.76	40,842.79
Water	44,472.55	44,472.55	3,629.76	40,842.79
TOTAL (kg/batch)	88,945.10	88,945.10	7,259.52	81,685.58
TOTAL (L/batch)	74,164.52	74,422.75	6,074.24	68,348.51

Stream Name	Fed-batch Sugar > SFR-1	Fed-Batch Sugar > SFR-2	Fed-Batch Sugar > SFR-3	Fed-Batch Sugar > FR-1
**Source**	**P-10**	**P-10**	**P-10**	**P-10**
**Destination**	**P-16**	**P-1**	**P-15**	**P-4**
Stream Properties				
Activity (U/ml)	0.00	0.00	0.00	0.00
Temperature (°C)	35.00	35.00	35.00	35.00
Pressure (bar)	1.01	1.01	1.01	1.01
Density (g/L)	1,195.13	1,195.13	1,195.13	1,195.13
Total Enthalpy (kW-h)	0.16	1.93	18.42	2,143.24
Specific Enthalpy (kcal/kg)	22.79	22.79	22.79	22.79
Heat Capacity (kcal/kg-°C)	0.65	0.65	0.65	0.65
Component Flowrates (kg/batch)				
Sucrose	3.06	36.39	347.74	40,455.60
Water	3.06	36.39	347.74	40,455.60
TOTAL (kg/batch)	6.13	72.78	695.47	80,911.20
TOTAL (L/batch)	5.13	60.90	581.92	67,700.57

Stream Name	S-110	S-124	S-121	S-127
**Source**	**P-7**	**P-7**	**P-7**	**P-7**
**Destination**	**P-12**	**P-22**	**P-20**	**P-24**
Stream Properties				
Activity (U/ml)	0.00	0.00	0.00	0.00
Temperature (°C)	35.00	35.00	35.00	35.00
Pressure (bar)	1.01	1.01	1.01	1.01
Density (g/L)	1,195.13	1,195.13	1,195.13	1,195.13
Total Enthalpy (kW-h)	0.04	0.91	9.11	182.24
Specific Enthalpy (kcal/kg)	22.79	22.79	22.79	22.79
Heat Capacity (kcal/kg-°C)	0.65	0.65	0.65	0.65
Component Flowrates (kg/batch)				
Sucrose	0.69	17.20	171.99	3,439.88
Water	0.69	17.20	171.99	3,439.88
TOTAL (kg/batch)	1.38	34.40	343.99	6,879.76
TOTAL (L/batch)	1.15	28.78	287.82	5,756.48

Stream Name	Initial Sugar to FR-1	Initial Sugar to SFR-3	Initial Sugar to SFR-2	S-114
**Source**	**P-24**	**P-20**	**P-22**	**INPUT**
**Destination**	**P-4**	**P-15**	**P-1**	**P-18**
Stream Properties				
Activity (U/ml)	0.00	0.00	0.00	0.00
Temperature (°C)	35.00	35.00	35.00	25.00
Pressure (bar)	1.01	1.01	1.01	1.01
Density (g/L)	1,013.02	1,003.77	1,002.54	994.70
Total Enthalpy (kW-h)	2,111.80	184.27	20.42	0.52
Specific Enthalpy (kcal/kg)	33.54	34.19	34.28	25.11
Heat Capacity (kcal/kg-°C)	0.95	0.97	0.98	1.00
Component Flowrates (kg/batch)				
Sucrose	3,439.88	171.99	17.20	0.00
Water	50,738.14	4,465.59	495.47	17.84
TOTAL (kg/batch)	54,178.02	4,637.58	512.66	17.84
TOTAL (L/batch)	53,481.43	4,620.15	511.36	17.93

Stream Name	S-115	Initial Sugar to SFR-1	Air input	S-153
**Source**	**P-18**	**P-12**	**INPUT**	**P-51**
**Destination**	**P-12**	**P-16**	**P-51**	**P-50**
Stream Properties				
Activity (U/ml)	0.00	0.00	0.00	0.00
Temperature (°C)	35.00	35.00	20.00	40.00
Pressure (bar)	1.01	1.01	1.01	6.01
Density (g/L)	991.06	1,003.36	1.20	6.66
Total Enthalpy (kW-h)	0.73	0.76	899.75	1,795.92
Specific Enthalpy (kcal/kg)	35.10	34.22	4.85	9.68
Heat Capacity (kcal/kg-°C)	1.00	0.97	0.24	0.24
Component Flowrates (kg/batch)				
Argon	0.00	0.00	1,467.91	1,467.91
Carb. Dioxide	0.00	0.00	63.82	63.82
Nitrogen	0.00	0.00	124,597.02	124,597.02
Oxygen	0.00	0.00	33,426.91	33,426.91
Sucrose	0.00	0.69	0.00	0.00
Water	17.84	18.53	0.00	0.00
TOTAL (kg/batch)	17.84	19.22	159,555.67	159,555.67
TOTAL (L/batch)	18.00	19.15	133,039,381.36	23,946,943.34

Stream Name	S-139	S-148	S-147	S-146
**Source**	**P-50**	**P-41**	**P-41**	**P-41**
**Destination**	**P-41**	**P-16**	**P-1**	**P-15**
Stream Properties				
Activity (U/ml)	0.00	0.00	0.00	0.00
Temperature (°C)	40.00	40.00	40.00	40.00
Pressure (bar)	6.01	6.01	6.01	6.01
Density (g/L)	6.66	6.66	6.66	6.66
Total Enthalpy (kW-h)	1,795.92	0.18	4.12	41.22
Specific Enthalpy (kcal/kg)	9.68	9.68	9.68	9.68
Heat Capacity (kcal/kg-°C)	0.24	0.24	0.24	0.24
Component Flowrates (kg/batch)				
Argon	1,467.91	0.15	3.36	33.69
Carb. Dioxide	63.82	0.01	0.15	1.46
Nitrogen	124,597.02	12.33	285.53	2,860.01
Oxygen	33,426.91	3.31	76.60	767.28
TOTAL (kg/batch)	159,555.67	15.79	365.64	3,662.46
TOTAL (L/batch)	23,946,943.34	2,370.52	54,877.13	549,680.74

Stream Name	S-143	Vent SFR-1	Inoculum to SFR-2	Vent FR-1
**Source**	**P-41**	**P-16**	**P-16**	**P-4**
**Destination**	**P-4**	**OUTPUT**	**P-1**	**P-49**
Stream Properties				
Activity (U/ml)	0.00	0.00	0.00	0.00
Temperature (°C)	40.00	35.00	35.00	34.98
Pressure (bar)	6.01	1.01	1.01	1.01
Density (g/L)	6.66	1.20	993.91	1.20
Total Enthalpy (kW-h)	1,750.40	0.34	1.35	3,241.32
Specific Enthalpy (kcal/kg)	9.68	15.98	35.10	15.51
Heat Capacity (kcal/kg-°C)	0.24	0.24	1.00	0.24
Component Flowrates (kg/batch)				
Amm. Sulfate	0.00	0.00	0.00	0.00
Argon	1,430.71	0.15	0.00	1,434.46
Biomass	0.00	0.00	1.69	0.00
Carb. Dioxide	62.20	2.62	0.00	24,000.84
NaH2PO4	0.00	0.00	0.00	0.00
Nitrogen	121,439.15	12.37	0.00	121,757.47
Oxygen	32,579.72	3.32	0.00	32,665.12
Sucrose	0.00	0.00	0.00	0.00
Water	0.00	0.00	31.38	0.00
TOTAL (kg/batch)	155,511.78	18.45	33.07	179,857.88
TOTAL (L/batch)	23,340,014.96	15,383.17	33.27	150,402,431.54

Stream Name	Emissions	Vent SFR-2	Inoculum to SFR-3	Vent SFR-3
**Source**	**P-49**	**P-1**	**P-1**	**P-15**
**Destination**	**OUTPUT**	**OUTPUT**	**P-15**	**OUTPUT**
Stream Properties				
Activity (U/ml)	0.00	0.00	0.00	0.00
Temperature (°C)	34.98	35.00	35.00	35.00
Pressure (bar)	1.01	1.01	1.01	1.01
Density (g/L)	1.20	1.18	992.80	1.18
Total Enthalpy (kW-h)	3,241.32	6.70	33.98	66.42
Specific Enthalpy (kcal/kg)	15.51	14.06	35.10	13.95
Heat Capacity (kcal/kg-°C)	0.24	0.24	1.00	0.24
Component Flowrates (kg/batch)				
Ammonium Chlori	0.00	0.00	0.01	0.00
Argon	1,434.46	3.37	0.00	33.79
Biomass	0.00	0.00	25.79	0.00
Carb. Dioxide	24,000.84	43.43	0.00	425.71
NaH2PO4	0.00	0.00	0.00	0.00
Nitrogen	121,757.47	286.32	0.00	2,867.93
Oxygen	32,665.12	76.81	0.00	769.41
Sucrose	0.00	0.00	0.03	0.00
Water	0.00	0.00	807.34	0.00
TOTAL (kg/batch)	179,857.88	409.94	833.17	4,096.83
TOTAL (L/batch)	150,402,431.54	346,233.57	839.21	3,462,683.96

Stream Name	Inoculum to FR-1	Mother Liquor	S-116	S-128
**Source**	**P-15**	**P-11**	**P-27**	**P-4**
**Destination**	**P-4**	**P-4**	**P-4**	**OUTPUT**
Stream Properties				
Activity (U/ml)	0.00	0.00	0.00	0.00
Temperature (°C)	35.00	9.00	35.31	12.55
Pressure (bar)	1.01	1.01	1.01	1.01
Density (g/L)	992.80	1,003.90	1,012.42	1,004.90
Total Enthalpy (kW-h)	339.45	1,664.97	1,017.54	2,683.56
Specific Enthalpy (kcal/kg)	35.10	9.00	34.37	12.50
Heat Capacity (kcal/kg-°C)	1.00	1.00	0.97	0.99
Component Flowrates (kg/batch)				
Amm. Sulfate	0.00	1.25	0.07	1.33
Ammonium Chlori	0.00	49.77	2.94	52.70
Biomass	259.67	0.00	5,064.31	5,064.31
NaH2PO4	0.00	13.47	0.79	14.27
pHBA (aq)	0.00	316.83	934.44	1,251.27
pHBA (solid)	0.00	310.49	0.00	310.49
Sucrose	0.04	1,020.65	60.20	1,080.85
Water	8,061.91	157,519.25	19,406.80	176,926.06
TOTAL (kg/batch)	8,321.61	159,231.72	25,469.56	184,701.27
TOTAL (L/batch)	8,381.97	158,612.78	25,157.05	183,800.97

Stream Name	S-113	S-105	Vent R-101	S-101
**Source**	**P-4**	**P-27**	**P-28**	**P-28**
**Destination**	**P-27**	**P-28**	**OUTPUT**	**P-11**
Stream Properties				
Activity (U/ml)	0.00	0.00	0.00	0.00
Temperature (°C)	35.00	35.31	5.00	5.00
Pressure (bar)	1.01	1.01	1.01	1.01
Density (g/L)	1,020.11	1,021.32	1.26	1,033.72
Total Enthalpy (kW-h)	6,444.31	5,483.79	0.24	780.95
Specific Enthalpy (kcal/kg)	32.41	32.41	1.23	4.61
Heat Capacity (kcal/kg-°C)	0.92	0.91	0.24	0.92
Component Flowrates (kg/batch)				
Amm. Sulfate	1.33	1.25	0.00	1.25
Ammonium Chlori	52.70	49.77	0.00	49.77
Argon	0.00	0.00	1.51	0.00
Biomass	5,064.31	0.00	0.00	0.00
Carb. Dioxide	0.00	0.00	0.07	0.00
NaH2PO4	14.27	13.47	0.00	13.47
Nitrogen	0.00	0.00	128.54	0.00
Oxygen	0.00	0.00	34.48	0.00
pHBA (aq)	16,775.87	15,841.43	0.00	316.83
pHBA (solid)	0.00	0.00	0.00	15,524.60
Sucrose	1,080.85	1,020.65	0.00	1,020.65
Water	148,080.91	128,674.10	0.00	128,674.10
TOTAL (kg/batch)	171,070.24	145,600.68	164.60	145,600.68
TOTAL (L/batch)	167,697.56	142,560.73	130,225.41	140,850.99

Stream Name	Wash Water	S-102	Humid Air	Final Product
Source	INPUT	P-11	P-14	P-14
Destination	P-11	P-14	OUTPUT	OUTPUT
Stream Properties				
Activity (U/ml)	0.00	0.00	0.00	0.00
Temperature (°C)	25.00	22.93	50.00	50.00
Pressure (bar)	1.01	1.86	1.01	1.01
Density (g/L)	994.70	1,211.06	1.08	1,303.70
Total Enthalpy (kW-h)	1,030.00	281.56	9,174.04	241.89
Specific Enthalpy (kcal/kg)	25.11	11.18	24.28	13.61
Heat Capacity (kcal/kg-°C)	1.00	0.49	0.25	0.27
Component Flowrates (kg/batch)				
Amm. Sulfate	0.00	0.00	0.00	0.00
Ammonium Chlori	0.00	0.00	0.00	0.00
Argon	0.00	0.00	2,931.98	0.00
Carb. Dioxide	0.00	0.00	127.48	0.00
NaH2PO4	0.00	0.00	0.00	0.00
Nitrogen	0.00	0.00	248,867.40	0.00
Oxygen	0.00	0.00	66,766.19	0.00
pHBA (aq)	0.00	0.00	0.00	0.00
pHBA (solid)	0.00	15,214.11	0.00	15,214.11
Sucrose	0.00	0.00	0.00	0.00
Water	35,295.46	6,450.31	6,373.86	76.45
TOTAL (kg/batch)	35,295.46	21,664.43	325,066.91	15,290.56
TOTAL (L/batch)	35,483.37	17,888.74	302,305,731.18	11,728.57

4. OVERALL COMPONENT BALANCE (kg/batch)

COMPONENT	INITIAL	INPUT	OUTPUT	FINAL	IN-OUT
Amm. Sulfate	0.00	56.91	1.33	0.00	55.58
Ammonium Chlori	0.00	2,257.68	52.70	0.00	2,204.98
Argon	4.40	4,399.89	4,405.26	2.87	- 3.84
Biomass	0.00	0.00	5,064.31	0.00	- 5,064.31
Carb. Dioxide	0.19	191.30	24,600.14	0.72	- 24,409.37
NaH2PO4	0.00	611.39	14.27	0.00	597.12
Nitrogen	373.11	373,464.42	373,920.02	243.55	- 326.04
Oxygen	100.10	100,193.11	100,315.33	65.34	- 87.47
pHBA (aq)	0.00	0.00	1,251.27	0.00	- 1,251.27
pHBA (solid)	0.00	0.00	15,524.60	0.00	- 15,524.60
Phosphoric Acid	0.00	181.86	181.86	0.00	0.00
Sodium Hydroxid	0.00	245.63	245.63	0.00	0.00
Sucrose	0.00	44,472.55	1,080.85	0.00	43,391.70
Water	0.00	219,765.68	219,765.68	0.00	0.00
TOTAL	477.79	745,840.43	746,423.26	312.47	417.51
				Overall Error:	0,056%

5. EQUIPMENT CONTENTS

SFR-3				
**Procedure**	**Operation**	**Time (in h)**	**Volume (in L)**	**Vapor (in kg)**
P-15	START	25.61	0.00	12.36(*)
P-15	TRANSFER-IN-SALTS (Transfer In)	26.61	2,549.41	12.36(*)
P-15	TRANSFER-IN-INITIAL-SUGAR (Transfer In)	27.61	7,169.56	12.36(*)
P-15	TRANSFER-IN-INOCULUM (Transfer In)	28.11	8,008.77	12.36(*)
P-15	FERMENT-2 (Batch Stoich. Fermentation)	40.11	8,381.97	2.48(*)
P-15	TRANSFER-OUT-1 (Transfer Out)	41.11	0.00	2.48(*)
P-15	CIP-1 (In-Place-Cleaning)	43.19	0.00	2.48(*)
P-15	SIP-1 (In-Place-Steaming)	45.19	0.00	2.48(*)

(*) Contains material in vapor phase other than Oxygen & Nitrogen

SFR-2				
**Procedure**	**Operation**	**Time (in h)**	**Volume (in L)**	**Vapor (in kg)**
P-1	START	14.11	0.00	1.24(*)
P-1	TRANSFER-IN-SALTS (Transfer In)	14.61	254.92	1.24(*)
P-1	TRANSFER-IN-INITIAL-SUGAR (Transfer In)	15.11	766.28	1.24(*)
P-1	TRANSFER-IN-INOCULUM (Transfer In)	15.61	799.55	1.24(*)
P-1	FERMENT-1 (Batch Stoich. Fermentation)	27.61	839.21	0.25(*)
P-1	TRANSFER-OUT-1 (Transfer Out)	28.11	0.00	0.25(*)
P-1	CIP-1 (In-Place-Cleaning)	30.19	0.00	0.25(*)
P-1	SIP-1 (In-Place-Steaming)	31.19	0.00	0.25(*)

(*) Contains material in vapor phase other than Oxygen & Nitrogen

SFR-1				
**Procedure**	**Operation**	**Time (in h)**	**Volume (in L)**	**Vapor (in kg)**
P-16	START	0.00	0.00	0.05(*)
P-16	TRANSFER-IN-PHOSPHATE (Transfer In)	0.25	3.42	0.05(*)
P-16	TRANSFER-IN-SULFATE (Transfer In)	0.50	6.89	0.05(*)
P-16	TRANSFER-IN-NH4Cl (Transfer In)	0.75	10.22	0.05(*)
P-16	TRANSFER-IN-INITIAL-SUGAR (Transfer In)	1.00	29.37	0.05(*)
P-16	FERMENT (Batch Stoich. Fermentation)	15.11	33.27	0.01(*)
P-16	TRANSFER-OUT (Transfer Out)	15.61	0.00	0.01(*)
P-16	CIP-1 (In-Place-Cleaning)	17.69	0.00	0.01(*)
P-16	SIP-1 (In-Place-Steaming)	18.19	0.00	0.01(*)

(*) Contains material in vapor phase other than Oxygen & Nitrogen

FR-1

Procedure	Operation	Time (in h)	Volume (in L)	Vapor (in kg)
P-4	START	39.11	0.00	231.29(*)
P-4	TRANSFER-IN-SULFATE (Transfer In)	40.11	17,330.48	231.29(*)
P-4	TRANSFER-IN-NH4Cl (Transfer In)	40.11	33,934.69	231.29(*)
P-4	TRANSFER-IN-PHOSPHATE (Transfer In)	40.11	50,988.58	231.29(*)
P-4	TRANSFER-IN-INITIAL-SUGAR (Transfer In)	40.11	104,470.00	231.29(*)
P-4	TRANSFER-IN-INOCULUM (Transfer In)	41.11	112,852.06	231.29(*)
P-4	FERMENT-1 (Batch Stoich. Fermentation)	76.27	167,697.56	34.01(*)
P-4	TRANSFER-OUT-2 (Transfer Out)	76.11	0.00	225.34(*)
P-4	TRANSFER-IN-1 (Transfer In)	76.11	25,157.05	196.24(*)
P-4	TRANSFER-IN-2 (Transfer In)	76.11	183,800.97	15.29(*)
P-4	TRANSFER-OUT-1 (Transfer Out)	78.27	0.00	241.47(*)
P-4	CIP-1 (In-Place-Cleaning)	80.35	0.00	241.47(*)
P-4	SIP-1 (In-Place-Steaming)	82.35	0.00	241.47(*)

(*) Contains material in vapor phase other than Oxygen & Nitrogen

R-102

Procedure	Operation	Time (in h)	Volume (in L)	Vapor (in kg)
P-28	START	40.11	0.00	31.13(*)
	AFTER AUTO-INIT	40.11	23,760.12	31.13(*)
P-28	REACT-1 (Batch Stoich. Reaction)	76.11	23,475.16	3.70(*)
P-28	END	76.11	0.00	3.70(*)

(*) Contains material in vapor phase other than Oxygen & Nitrogen

BCFBD-101

Procedure	Operation	Time (in h)	Volume (in L)	Vapor (in kg)
P-11	START	40.11	0.00	3.84(*)
P-11	FILTER-1 (Cloth Filtration)	75.61	1,478.47	3.84(*)
P-11	CAKE-WASH-1 (Cake Wash)	75.86	1,490.73	3.84(*)
P-11	TRANSFER-OUT-1 (Transfer Out)	76.11	0.00	3.84(*)

(*) Contains material in vapor phase other than Oxygen & Nitrogen
