# Supplementary material for: Economic Process Evaluation and Environmental Life-Cycle Assessment of Bio-Aromatics Production
Source: Front Bioeng Biotechnol. 2020 May 13;8:403. doi: 10.3389/fbioe.2020.00403 (PMC7237583; doi:10.3389/fbioe.2020.00403)
Supplement: Supplementary file 1 [file Data_Sheet_1.zip › Sc_9.pdf]

# Materials & Streams Report

März 25, 2020

## *for Supplementary_9_yeast_best_case_cane_sugar_upscaled*

## 1. OVERALL PROCESS DATA

| | |
|---|---|
| Annual Operating Time | 7,918.35 h |
| Unit Production Ref. Rate | 50,000,000.00 kg MP/yr |
| Batch Size | 76,452.60 kg MP |
| Recipe Batch Time | 82.35 h |
| Recipe Cycle Time | 12.00 h |
| Number of Batches per Year | 654.00 |

MP = Total Flow of Stream 'Final Product'

## 2.1 STARTING MATERIAL REQUIREMENTS (per Section)

| Section | Starting Material | Active Product | Amount Needed (kg Sin/kg MP) | Molar Yield (%) | Mass Yield (%) | Gross Mass Yield (%) |
|---|---|---|---|---|---|---|
| Fermentation Section | (none) | (none) | 0.00 | Unknown | Unknown | Unknown |
| Downstream Section | (none) | (none) | 0.00 | Unknown | Unknown | Unknown |

Sin = Section Starting Material, Aout = Section Active Product

## 2.2 BULK MATERIALS (Entire Process)

| Material | kg/yr | kg/batch | kg/kg MP |
|---|---|---|---|
| Air | 1,563,868,771 | 2,391,236.65 | 31.28 |
| Amm. Sulfate | 186,097 | 284.55 | 0.00 |
| Ammonium Chlori | 7,382,592 | 11,288.37 | 0.15 |
| H3PO4 (2%) | 13,587,321 | 20,775.72 | 0.27 |
| NaH2PO4 | 1,999,241 | 3,056.94 | 0.04 |
| NaOH (0.5 M) | 21,143,056 | 32,328.83 | 0.42 |
| Sucrose | 145,424,816 | 222,362.10 | 2.91 |
| Water | 624,185,640 | 954,412.29 | 12.48 |
| TOTAL | 2,377,777,534 | 3,635,745.47 | 47.56 |

## 2.3 BULK MATERIALS (per Section)

### SECTIONS IN: Main Branch

#### Fermentation Section

| Material | kg/yr | kg/batch | kg/kg MP |
|---|---|---|---|
| Air | 521,745,521 | 797,776.03 | 10.43 |
| Amm. Sulfate | 186,097 | 284.55 | 0.00 |
| Ammonium Chlori | 7,382,592 | 11,288.37 | 0.15 |
| H3PO4 (2%) | 13,587,321 | 20,775.72 | 0.27 |
| NaH2PO4 | 1,999,241 | 3,056.94 | 0.04 |
| NaOH (0.5 M) | 21,143,056 | 32,328.83 | 0.42 |
| Sucrose | 145,424,816 | 222,362.10 | 2.91 |
| Water | 508,769,811 | 777,935.49 | 10.18 |
| TOTAL | 1,220,238,455 | 1,865,808.03 | 24.40 |

#### Downstream Section

| Material | kg/yr | kg/batch | kg/kg MP |
|---|---|---|---|
| Air | 1,042,123,250 | 1,593,460.63 | 20.84 |
| Water | 115,415,829 | 176,476.80 | 2.31 |
| TOTAL | 1,157,539,080 | 1,769,937.43 | 23.15 |

## 2.4 BULK MATERIALS (per Material)

#### Air

| Procedure | % Total | kg/yr | kg/batch | kg/kg MP |
|---|---|---|---|---|
| Fermentation Section (Main Branch) | | | | |
| P-51 | 33.36 | 521,745,521 | 797,776.03 | 10.43 |
| Downstream Section (Main Branch) | | | | |
| P-3 | 66.64 | 1,042,123,250 | 1,593,460.63 | 20.84 |
| TOTAL | 100.00 | 1,563,868,771 | 2,391,236.65 | 31.28 |

#### Amm. Sulfate

| Procedure | % Total | kg/yr | kg/batch | kg/kg MP |
|---|---|---|---|---|
| Fermentation Section (Main Branch) | | | | |
| P-36 | 100.00 | 186,097 | 284.55 | 0.00 |
| TOTAL | 100.00 | 186,097 | 284.55 | 0.00 |

## Ammonium Chlori

| Procedure | % Total | kg/yr | kg/batch | kg/kg MP |
|---|---|---|---|---|
| Fermentation Section (Main Branch) | | | | |
| P-38 | 100.00 | 7,382,592 | 11,288.37 | 0.15 |
| TOTAL | 100.00 | 7,382,592 | 11,288.37 | 0.15 |

## H3PO4 (2%)

| Procedure | % Total | kg/yr | kg/batch | kg/kg MP |
|---|---|---|---|---|
| Fermentation Section (Main Branch) | | | | |
| P-4 | 54.42 | 7,394,535 | 11,306.63 | 0.15 |
| P-1 | 10.11 | 1,374,183 | 2,101.20 | 0.03 |
| P-15 | 32.02 | 4,350,039 | 6,651.44 | 0.09 |
| P-16 | 3.45 | 468,564 | 716.46 | 0.01 |
| TOTAL | 100.00 | 13,587,321 | 20,775.72 | 0.27 |

## NaH2PO4

| Procedure | % Total | kg/yr | kg/batch | kg/kg MP |
|---|---|---|---|---|
| Fermentation Section (Main Branch) | | | | |
| P-34 | 100.00 | 1,999,241 | 3,056.94 | 0.04 |
| TOTAL | 100.00 | 1,999,241 | 3,056.94 | 0.04 |

## NaOH (0.5 M)

| Procedure | % Total | kg/yr | kg/batch | kg/kg MP |
|---|---|---|---|---|
| Fermentation Section (Main Branch) | | | | |
| P-4 | 82.31 | 17,403,803 | 26,611.32 | 0.35 |
| P-1 | 6.56 | 1,386,121 | 2,119.45 | 0.03 |
| P-15 | 8.89 | 1,880,498 | 2,875.38 | 0.04 |
| P-16 | 2.24 | 472,634 | 722.68 | 0.01 |
| TOTAL | 100.00 | 21,143,056 | 32,328.83 | 0.42 |

## Sucrose

| Procedure | % Total | kg/yr | kg/batch | kg/kg MP |
|---|---|---|---|---|
| Fermentation Section (Main Branch) | | | | |
| P-9 | 100.00 | 145,424,816 | 222,362.10 | 2.91 |
| TOTAL | 100.00 | 145,424,816 | 222,362.10 | 2.91 |

## Water

| Procedure | % Total | kg/yr | kg/batch | kg/kg MP |
|---|---|---|---|---|
| Fermentation Section (Main Branch) | | | | |
| P-4 | 2.62 | 16,350,015 | 25,000.02 | 0.33 |
| P-34 | 9.19 | 57,347,067 | 87,686.65 | 1.15 |
| P-36 | 9.48 | 59,160,435 | 90,459.38 | 1.18 |
| P-38 | 8.33 | 51,963,715 | 79,455.22 | 1.04 |
| P-9 | 23.30 | 145,424,816 | 222,362.10 | 2.91 |
| P-18 | 0.01 | 58,323 | 89.18 | 0.00 |
| P-21 | 2.25 | 14,040,025 | 21,467.93 | 0.28 |
| P-23 | 0.25 | 1,563,936 | 2,391.34 | 0.03 |
| P-25 | 24.78 | 154,664,845 | 236,490.59 | 3.09 |
| P-1 | 0.49 | 3,038,449 | 4,645.95 | 0.06 |

| | | | | |
|---|---|---|---|---|
| P-15 | 0.66 | 4,122,149 | 6,302.98 | 0.08 |
| P-16 | 0.17 | 1,036,039 | 1,584.16 | 0.02 |
| Downstream Section (Main Branch) | | | | |
| P-11 | 18.49 | 115,415,829 | 176,476.80 | 2.31 |
| TOTAL | 100.00 | 624,185,640 | 954,412.29 | 12.48 |

## 2.5 BULK MATERIALS: SECTION TOTALS (kg/kg MP)

| Raw Material | Fermentation Section | Downstream Section |
|---|---|---|
| Air | 10.43 | 20.84 |
| Amm. Sulfate | 0.00 | 0.00 |
| Ammonium Chlori | 0.15 | 0.00 |
| H3PO4 (2%) | 0.27 | 0.00 |
| NaH2PO4 | 0.04 | 0.00 |
| NaOH (0.5 M) | 0.42 | 0.00 |
| Sucrose | 2.91 | 0.00 |
| Water | 10.18 | 2.31 |
| TOTAL | 24.40 | 23.15 |

## 2.6 BULK MATERIALS: SECTION TOTALS (kg/batch)

| Raw Material | Fermentation Section | Downstream Section |
|---|---|---|
| Air | 797,776.03 | 1,593,460.63 |
| Amm. Sulfate | 284.55 | 0.00 |
| Ammonium Chlori | 11,288.37 | 0.00 |
| H3PO4 (2%) | 20,775.72 | 0.00 |
| NaH2PO4 | 3,056.94 | 0.00 |
| NaOH (0.5 M) | 32,328.83 | 0.00 |
| Sucrose | 222,362.10 | 0.00 |
| Water | 777,935.49 | 176,476.80 |
| TOTAL | 1,865,808.03 | 1,769,937.43 |

## 2.7 BULK MATERIALS: SECTION TOTALS (kg/yr)

| Raw Material | Fermentation Section | Downstream Section |
|---|---|---|
| Air | 521,745,521 | 1,042,123,250 |
| Amm. Sulfate | 186,097 | 0 |
| Ammonium Chlori | 7,382,592 | 0 |
| H3PO4 (2%) | 13,587,321 | 0 |
| NaH2PO4 | 1,999,241 | 0 |
| NaOH (0.5 M) | 21,143,056 | 0 |
| Sucrose | 145,424,816 | 0 |
| Water | 508,769,811 | 115,415,829 |
| TOTAL | 1,220,238,455 | 1,157,539,080 |

## 3. STREAM DETAILS

| Stream Name | Air for Drying | S-104 | Water for NH4Cl | NH4Cl |
|---|---|---|---|---|
| Source | INPUT | P-3 | INPUT | INPUT |
| Destination | P-3 | P-14 | P-38 | P-38 |
| Stream Properties | | | | |
| Activity (U/ml) | 0.00 | 0.00 | 0.00 | 0.00 |
| Temperature (°C) | 25.00 | 37.66 | 10.00 | 20.00 |
| Pressure (bar) | 1.01 | 1.21 | 1.01 | 1.01 |
| Density (g/L) | 1.18 | 1.35 | 1,000.17 | 1,519.00 |
| Total Enthalpy (kW-h) | 11,222.63 | 16,886.13 | 930.36 | 98.60 |
| Specific Enthalpy (kcal/kg) | 6.06 | 9.12 | 10.07 | 7.52 |
| Heat Capacity (kcal/kg-°C) | 0.24 | 0.24 | 1.01 | 0.38 |
| Component Flowrates (kg/batch) | | | | |
| Ammonium Chlori | 0.00 | 0.00 | 0.00 | 11,288.37 |
| Argon | 14,659.84 | 14,659.84 | 0.00 | 0.00 |
| Carb. Dioxide | 637.38 | 637.38 | 0.00 | 0.00 |
| Nitrogen | 1,244,333.40 | 1,244,333.40 | 0.00 | 0.00 |
| Oxygen | 333,830.00 | 333,830.00 | 0.00 | 0.00 |
| Water | 0.00 | 0.00 | 79,455.22 | 0.00 |
| TOTAL (kg/batch) | 1,593,460.63 | 1,593,460.63 | 79,455.22 | 11,288.37 |
| TOTAL (L/batch) | 1,351,307,636.87 | 1,176,452,701.93 | 79,441.57 | 7,431.45 |

| Stream Name | Cl-Solution | S-129 | NH4Cl to SFR-1 | NH4Cl to SFR-2 |
|---|---|---|---|---|
| Source | P-38 | P-37 | P-5 | P-5 |
| Destination | P-37 | P-5 | P-16 | P-64 |
| Stream Properties | | | | |
| Activity (U/ml) | 0.00 | 0.00 | 0.00 | 0.00 |
| Temperature (°C) | 10.50 | 35.00 | 35.00 | 35.00 |
| Pressure (bar) | 1.01 | 1.01 | 1.01 | 1.01 |
| Density (g/L) | 1,044.38 | 1,035.84 | 1,035.84 | 1,035.84 |
| Total Enthalpy (kW-h) | 1,028.95 | 3,413.98 | 0.65 | 16.18 |
| Specific Enthalpy (kcal/kg) | 9.76 | 32.37 | 32.37 | 32.37 |
| Heat Capacity (kcal/kg-°C) | 0.93 | 0.92 | 0.92 | 0.92 |
| Component Flowrates (kg/batch) | | | | |
| Ammonium Chlori | 11,288.37 | 11,288.37 | 2.14 | 53.48 |
| Water | 79,455.22 | 79,455.22 | 15.10 | 376.46 |
| TOTAL (kg/batch) | 90,743.59 | 90,743.59 | 17.24 | 429.94 |
| TOTAL (L/batch) | 86,887.60 | 87,603.46 | 16.64 | 415.07 |

| Stream Name | NH4Cl to SFR-3 | NH4Cl to FR-1 | Water for NH4SO4 | NH4SO4 |
|---|---|---|---|---|
| **Source** | **P-5** | **P-5** | **INPUT** | **INPUT** |
| **Destination** | **P-65** | **P-4** | **P-36** | **P-36** |
| Stream Properties | | | | |
| Activity (U/ml) | 0.00 | 0.00 | 0.00 | 0.00 |
| Temperature (°C) | 35.00 | 35.00 | 10.00 | 20.00 |
| Pressure (bar) | 1.01 | 1.01 | 1.01 | 1.01 |
| Density (g/L) | 1,035.84 | 1,035.84 | 1,000.17 | 1,769.00 |
| Total Enthalpy (kW-h) | 161.77 | 3,235.39 | 1,059.21 | 2.25 |
| Specific Enthalpy (kcal/kg) | 32.37 | 32.37 | 10.07 | 6.80 |
| Heat Capacity (kcal/kg-°C) | 0.92 | 0.92 | 1.01 | 0.34 |
| Component Flowrates (kg/batch) | | | | |
| Amm. Sulfate | 0.00 | 0.00 | 0.00 | 284.55 |
| Ammonium Chlori | 534.89 | 10,697.85 | 0.00 | 0.00 |
| Water | 3,764.91 | 75,298.76 | 90,459.38 | 0.00 |
| TOTAL (kg/batch) | 4,299.79 | 85,996.61 | 90,459.38 | 284.55 |
| TOTAL (L/batch) | 4,151.00 | 83,020.75 | 90,443.84 | 160.85 |

| Stream Name | SO4-Solution | S-138 | Sulfate to SFR-1 | Sulfate to SFR-2 |
|---|---|---|---|---|
| **Source** | **P-36** | **P-35** | **P-6** | **P-6** |
| **Destination** | **P-35** | **P-6** | **P-16** | **P-64** |
| Stream Properties | | | | |
| Activity (U/ml) | 0.00 | 0.00 | 0.00 | 0.00 |
| Temperature (°C) | 10.01 | 35.00 | 35.00 | 35.00 |
| Pressure (bar) | 1.01 | 1.01 | 1.01 | 1.01 |
| Density (g/L) | 1,001.53 | 992.43 | 992.43 | 992.43 |
| Total Enthalpy (kW-h) | 1,061.45 | 3,694.29 | 0.70 | 17.50 |
| Specific Enthalpy (kcal/kg) | 10.06 | 35.03 | 35.03 | 35.03 |
| Heat Capacity (kcal/kg-°C) | 1.00 | 1.00 | 1.00 | 1.00 |
| Component Flowrates (kg/batch) | | | | |
| Amm. Sulfate | 284.55 | 284.55 | 0.05 | 1.35 |
| Water | 90,459.38 | 90,459.38 | 17.19 | 428.60 |
| TOTAL (kg/batch) | 90,743.93 | 90,743.93 | 17.24 | 429.94 |
| TOTAL (L/batch) | 90,605.05 | 91,436.30 | 17.37 | 433.23 |

| Stream Name | Sulfate to SFR-3 | Sulfate to FR-1 | Water for NaH2PO4 | NaH2PO4 |
|---|---|---|---|---|
| **Source** | **P-6** | **P-6** | **INPUT** | **INPUT** |
| **Destination** | **P-65** | **P-4** | **P-34** | **P-34** |
| Stream Properties | | | | |
| Activity (U/ml) | 0.00 | 0.00 | 0.00 | 0.00 |
| Temperature (°C) | 35.00 | 35.00 | 10.00 | 20.00 |
| Pressure (bar) | 1.01 | 1.01 | 1.01 | 1.01 |
| Density (g/L) | 992.43 | 992.43 | 1,000.17 | 2,040.00 |
| Total Enthalpy (kW-h) | 175.05 | 3,501.03 | 1,026.74 | 10.65 |
| Specific Enthalpy (kcal/kg) | 35.03 | 35.03 | 10.07 | 3.00 |
| Heat Capacity (kcal/kg-°C) | 1.00 | 1.00 | 1.01 | 0.15 |
| Component Flowrates (kg/batch) | | | | |
| Amm. Sulfate | 13.48 | 269.67 | 0.00 | 0.00 |
| NaH2PO4 | 0.00 | 0.00 | 0.00 | 3,056.94 |
| Water | 4,286.33 | 85,727.27 | 87,686.65 | 0.00 |
| TOTAL (kg/batch) | 4,299.81 | 85,996.94 | 87,686.65 | 3,056.94 |
| TOTAL (L/batch) | 4,332.62 | 86,653.08 | 87,671.58 | 1,498.50 |

| Stream Name | PO4-Solution | S-108 | Phosphate to SFR-1 | Phosphate to SFR-2 |
|---|---|---|---|---|
| **Source** | **P-34** | **P-33** | **P-2** | **P-2** |
| **Destination** | **P-33** | **P-2** | **P-16** | **P-64** |
| Stream Properties | | | | |
| Activity (U/ml) | 0.00 | 0.00 | 0.00 | 0.00 |
| Temperature (°C) | 10.05 | 35.00 | 35.00 | 35.00 |
| Pressure (bar) | 1.01 | 1.01 | 1.01 | 1.01 |
| Density (g/L) | 1,017.63 | 1,008.53 | 1,008.53 | 1,008.53 |
| Total Enthalpy (kW-h) | 1,037.39 | 3,595.88 | 0.68 | 17.04 |
| Specific Enthalpy (kcal/kg) | 9.84 | 34.10 | 34.10 | 34.10 |
| Heat Capacity (kcal/kg-°C) | 0.98 | 0.97 | 0.97 | 0.97 |
| Component Flowrates (kg/batch) | | | | |
| NaH2PO4 | 3,056.94 | 3,056.94 | 0.58 | 14.48 |
| Water | 87,686.65 | 87,686.65 | 16.66 | 415.46 |
| TOTAL (kg/batch) | 90,743.59 | 90,743.59 | 17.24 | 429.94 |
| TOTAL (L/batch) | 89,171.72 | 89,976.20 | 17.10 | 426.31 |

| Stream Name | Phosphate to SFR-3 | Phosphate to FR-1 | Salts to SFR-3 | Salts to SFR-2 |
|---|---|---|---|---|
| **Source** | **P-2** | **P-2** | **P-65** | **P-64** |
| **Destination** | **P-65** | **P-4** | **P-15** | **P-1** |
| Stream Properties | | | | |
| Activity (U/ml) | 0.00 | 0.00 | 0.00 | 0.00 |
| Temperature (°C) | 35.00 | 35.00 | 35.00 | 35.00 |
| Pressure (bar) | 1.01 | 1.01 | 1.01 | 1.01 |
| Density (g/L) | 1,008.53 | 1,008.53 | 1,011.95 | 1,011.95 |
| Total Enthalpy (kW-h) | 170.39 | 3,407.77 | 507.21 | 50.72 |
| Specific Enthalpy (kcal/kg) | 34.10 | 34.10 | 33.83 | 33.83 |
| Heat Capacity (kcal/kg-°C) | 0.97 | 0.97 | 0.96 | 0.96 |
| Component Flowrates (kg/batch) | | | | |
| Amm. Sulfate | 0.00 | 0.00 | 13.48 | 1.35 |
| Ammonium Chlori | 0.00 | 0.00 | 534.89 | 53.48 |
| NaH2PO4 | 144.85 | 2,897.03 | 144.85 | 14.48 |
| Water | 4,154.94 | 83,099.58 | 12,206.18 | 1,220.51 |
| TOTAL (kg/batch) | 4,299.79 | 85,996.61 | 12,899.40 | 1,289.83 |
| TOTAL (L/batch) | 4,263.43 | 85,269.36 | 12,747.05 | 1,274.60 |

| Stream Name | S-123 | S-125 | S-112 | S-118 |
|---|---|---|---|---|
| **Source** | **INPUT** | **P-25** | **INPUT** | **P-21** |
| **Destination** | **P-25** | **P-24** | **P-21** | **P-20** |
| Stream Properties | | | | |
| Activity (U/ml) | 0.00 | 0.00 | 0.00 | 0.00 |
| Temperature (°C) | 25.00 | 35.00 | 25.00 | 35.00 |
| Pressure (bar) | 1.01 | 1.01 | 1.01 | 1.01 |
| Density (g/L) | 994.70 | 991.06 | 994.70 | 991.06 |
| Total Enthalpy (kW-h) | 6,901.30 | 9,647.80 | 626.48 | 875.80 |
| Specific Enthalpy (kcal/kg) | 25.11 | 35.10 | 25.11 | 35.10 |
| Heat Capacity (kcal/kg-°C) | 1.00 | 1.00 | 1.00 | 1.00 |
| Component Flowrates (kg/batch) | | | | |
| Water | 236,490.59 | 236,490.59 | 21,467.93 | 21,467.93 |
| TOTAL (kg/batch) | 236,490.59 | 236,490.59 | 21,467.93 | 21,467.93 |
| TOTAL (L/batch) | 237,749.63 | 238,624.05 | 21,582.22 | 21,661.60 |

| Stream Name | S-120 | S-122 | Water for 50% Sucrose | Process Sucrose |
|---|---|---|---|---|
| **Source** | **INPUT** | **P-23** | **INPUT** | **INPUT** |
| **Destination** | **P-23** | **P-22** | **P-9** | **P-9** |
| Stream Properties | | | | |
| Activity (U/ml) | 0.00 | 0.00 | 0.00 | 0.00 |
| Temperature (°C) | 25.00 | 35.00 | 25.00 | 25.00 |
| Pressure (bar) | 1.01 | 1.01 | 1.01 | 1.01 |
| Density (g/L) | 994.70 | 991.06 | 994.70 | 1,509.84 |
| Total Enthalpy (kW-h) | 69.78 | 97.56 | 6,489.00 | 1,934.83 |
| Specific Enthalpy (kcal/kg) | 25.11 | 35.10 | 25.11 | 7.49 |
| Heat Capacity (kcal/kg-°C) | 1.00 | 1.00 | 1.00 | 0.30 |
| Component Flowrates (kg/batch) | | | | |
| Sucrose | 0.00 | 0.00 | 0.00 | 222,362.10 |
| Water | 2,391.34 | 2,391.34 | 222,362.10 | 0.00 |
| TOTAL (kg/batch) | 2,391.34 | 2,391.34 | 222,362.10 | 222,362.10 |
| TOTAL (L/batch) | 2,404.07 | 2,412.91 | 223,545.93 | 147,275.60 |

| Stream Name | S-144 | S-106 | Batch Sucrose | Fed-Batch Sucrose |
|---|---|---|---|---|
| **Source** | **P-9** | **P-8** | **Sucrose Storage** | **Sucrose Storage** |
| **Destination** | **P-8** | **Sucrose Storage** | **P-7** | **P-10** |
| Stream Properties | | | | |
| Activity (U/ml) | 0.00 | 0.00 | 0.00 | 0.00 |
| Temperature (°C) | 25.00 | 35.00 | 35.00 | 35.00 |
| Pressure (bar) | 1.01 | 1.01 | 1.01 | 1.01 |
| Density (g/L) | 1,199.29 | 1,195.13 | 1,195.13 | 1,195.13 |
| Total Enthalpy (kW-h) | 8,423.83 | 11,780.18 | 961.48 | 10,818.71 |
| Specific Enthalpy (kcal/kg) | 16.30 | 22.79 | 22.79 | 22.79 |
| Heat Capacity (kcal/kg-°C) | 0.65 | 0.65 | 0.65 | 0.65 |
| Component Flowrates (kg/batch) | | | | |
| Sucrose | 222,362.10 | 222,362.10 | 18,148.75 | 204,213.35 |
| Water | 222,362.10 | 222,362.10 | 18,148.75 | 204,213.35 |
| TOTAL (kg/batch) | 444,724.21 | 444,724.21 | 36,297.50 | 408,426.71 |
| TOTAL (L/batch) | 370,821.53 | 372,112.67 | 30,371.09 | 341,741.58 |

| Stream Name | Fed-batch Sugar > SFR-1 | Fed-Batch Sugar > SFR-2 | Fed-Batch Sugar > SFR-3 | Fed-Batch Sugar > FR-1 |
|---|---|---|---|---|
| **Source** | **P-10** | **P-10** | **P-10** | **P-10** |
| **Destination** | **P-16** | **P-1** | **P-15** | **P-4** |
| Stream Properties | | | | |
| Activity (U/ml) | 0.00 | 0.00 | 0.00 | 0.00 |
| Temperature (°C) | 35.00 | 35.00 | 35.00 | 35.00 |
| Pressure (bar) | 1.01 | 1.01 | 1.01 | 1.01 |
| Density (g/L) | 1,195.13 | 1,195.13 | 1,195.13 | 1,195.13 |
| Total Enthalpy (kW-h) | 0.81 | 9.64 | 92.11 | 10,716.15 |
| Specific Enthalpy (kcal/kg) | 22.79 | 22.79 | 22.79 | 22.79 |
| Heat Capacity (kcal/kg-°C) | 0.65 | 0.65 | 0.65 | 0.65 |
| Component Flowrates (kg/batch) | | | | |
| Sucrose | 15.32 | 181.95 | 1,738.67 | 202,277.41 |
| Water | 15.32 | 181.95 | 1,738.67 | 202,277.41 |
| TOTAL (kg/batch) | 30.63 | 363.91 | 3,477.34 | 404,554.82 |
| TOTAL (L/batch) | 25.63 | 304.49 | 2,909.59 | 338,501.87 |

| Stream Name | S-110 | S-124 | S-121 | S-127 |
|---|---|---|---|---|
| **Source** | **P-7** | **P-7** | **P-7** | **P-7** |
| **Destination** | **P-12** | **P-22** | **P-20** | **P-24** |
| Stream Properties | | | | |
| Activity (U/ml) | 0.00 | 0.00 | 0.00 | 0.00 |
| Temperature (°C) | 35.00 | 35.00 | 35.00 | 35.00 |
| Pressure (bar) | 1.01 | 1.01 | 1.01 | 1.01 |
| Density (g/L) | 1,195.13 | 1,195.13 | 1,195.13 | 1,195.13 |
| Total Enthalpy (kW-h) | 0.18 | 4.56 | 45.56 | 911.18 |
| Specific Enthalpy (kcal/kg) | 22.79 | 22.79 | 22.79 | 22.79 |
| Heat Capacity (kcal/kg-°C) | 0.65 | 0.65 | 0.65 | 0.65 |
| Component Flowrates (kg/batch) | | | | |
| Sucrose | 3.45 | 85.99 | 859.96 | 17,199.35 |
| Water | 3.45 | 85.99 | 859.96 | 17,199.35 |
| TOTAL (kg/batch) | 6.90 | 171.98 | 1,719.92 | 34,398.71 |
| TOTAL (L/batch) | 5.77 | 143.90 | 1,439.10 | 28,782.32 |

| Stream Name | Initial Sugar to FR-1 | Initial Sugar to SFR-3 | Initial Sugar to SFR-2 | S-114 |
|---|---|---|---|---|
| **Source** | **P-24** | **P-20** | **P-22** | **INPUT** |
| **Destination** | **P-4** | **P-15** | **P-1** | **P-18** |
| Stream Properties | | | | |
| Activity (U/ml) | 0.00 | 0.00 | 0.00 | 0.00 |
| Temperature (°C) | 35.00 | 35.00 | 35.00 | 25.00 |
| Pressure (bar) | 1.01 | 1.01 | 1.01 | 1.01 |
| Density (g/L) | 1,013.02 | 1,003.77 | 1,002.54 | 994.70 |
| Total Enthalpy (kW-h) | 10,558.98 | 921.36 | 102.11 | 2.60 |
| Specific Enthalpy (kcal/kg) | 33.54 | 34.19 | 34.28 | 25.11 |
| Heat Capacity (kcal/kg-°C) | 0.95 | 0.97 | 0.98 | 1.00 |
| Component Flowrates (kg/batch) | | | | |
| Sucrose | 17,199.35 | 859.96 | 85.99 | 0.00 |
| Water | 253,689.94 | 22,327.89 | 2,477.33 | 89.18 |
| TOTAL (kg/batch) | 270,889.29 | 23,187.85 | 2,563.32 | 89.18 |
| TOTAL (L/batch) | 267,406.37 | 23,100.70 | 2,556.81 | 89.65 |

| Stream Name | S-115 | Initial Sugar to SFR-1 | Air input | S-153 |
|---|---|---|---|---|
| **Source** | **P-18** | **P-12** | **INPUT** | **P-51** |
| **Destination** | **P-12** | **P-16** | **P-51** | **P-50** |
| Stream Properties | | | | |
| Activity (U/ml) | 0.00 | 0.00 | 0.00 | 0.00 |
| Temperature (°C) | 35.00 | 35.00 | 20.00 | 40.00 |
| Pressure (bar) | 1.01 | 1.01 | 1.01 | 6.01 |
| Density (g/L) | 991.06 | 1,003.36 | 1.20 | 6.66 |
| Total Enthalpy (kW-h) | 3.64 | 3.82 | 4,498.75 | 8,979.58 |
| Specific Enthalpy (kcal/kg) | 35.10 | 34.22 | 4.85 | 9.68 |
| Heat Capacity (kcal/kg-°C) | 1.00 | 0.97 | 0.24 | 0.24 |
| Component Flowrates (kg/batch) | | | | |
| Argon | 0.00 | 0.00 | 7,339.54 | 7,339.54 |
| Carb. Dioxide | 0.00 | 0.00 | 319.11 | 319.11 |
| Nitrogen | 0.00 | 0.00 | 622,983.30 | 622,983.30 |
| Oxygen | 0.00 | 0.00 | 167,134.08 | 167,134.08 |
| Sucrose | 0.00 | 3.45 | 0.00 | 0.00 |
| Water | 89.18 | 92.63 | 0.00 | 0.00 |
| TOTAL (kg/batch) | 89.18 | 96.07 | 797,776.03 | 797,776.03 |
| TOTAL (L/batch) | 89.98 | 95.75 | 665,194,975.60 | 119,734,369.11 |

| Stream Name | S-139 | S-148 | S-147 | S-146 |
|---|---|---|---|---|
| **Source** | **P-50** | **P-41** | **P-41** | **P-41** |
| **Destination** | **P-41** | **P-16** | **P-1** | **P-15** |
| Stream Properties | | | | |
| Activity (U/ml) | 0.00 | 0.00 | 0.00 | 0.00 |
| Temperature (°C) | 40.00 | 40.00 | 40.00 | 40.00 |
| Pressure (bar) | 6.01 | 6.01 | 6.01 | 6.01 |
| Density (g/L) | 6.66 | 6.66 | 6.66 | 6.66 |
| Total Enthalpy (kW-h) | 8,979.58 | 0.89 | 20.58 | 206.12 |
| Specific Enthalpy (kcal/kg) | 9.68 | 9.68 | 9.68 | 9.68 |
| Heat Capacity (kcal/kg-°C) | 0.24 | 0.24 | 0.24 | 0.24 |
| Component Flowrates (kg/batch) | | | | |
| Argon | 7,339.54 | 0.73 | 16.82 | 168.47 |
| Carb. Dioxide | 319.11 | 0.03 | 0.73 | 7.32 |
| Nitrogen | 622,983.30 | 61.67 | 1,427.64 | 14,300.03 |
| Oxygen | 167,134.08 | 16.54 | 383.01 | 3,836.41 |
| TOTAL (kg/batch) | 797,776.03 | 78.97 | 1,828.19 | 18,312.24 |
| TOTAL (L/batch) | 119,734,369.11 | 11,852.56 | 274,384.84 | 2,748,395.72 |

| Stream Name | S-143 | Vent SFR-1 | Inoculum to SFR-2 | Vent FR-1 |
|---|---|---|---|---|
| **Source** | **P-41** | **P-16** | **P-16** | **P-4** |
| **Destination** | **P-4** | **OUTPUT** | **P-1** | **P-49** |
| Stream Properties | | | | |
| Activity (U/ml) | 0.00 | 0.00 | 0.00 | 0.00 |
| Temperature (°C) | 40.00 | 35.00 | 35.00 | 34.98 |
| Pressure (bar) | 6.01 | 1.01 | 1.01 | 1.01 |
| Density (g/L) | 6.66 | 1.20 | 993.91 | 1.20 |
| Total Enthalpy (kW-h) | 8,751.99 | 1.71 | 6.74 | 16,206.57 |
| Specific Enthalpy (kcal/kg) | 9.68 | 15.98 | 35.10 | 15.51 |
| Heat Capacity (kcal/kg-°C) | 0.24 | 0.24 | 1.00 | 0.24 |
| Component Flowrates (kg/batch) | | | | |
| Amm. Sulfate | 0.00 | 0.00 | 0.00 | 0.00 |
| Argon | 7,153.52 | 0.73 | 0.00 | 7,172.27 |
| Biomass | 0.00 | 0.00 | 8.44 | 0.00 |
| Carb. Dioxide | 311.02 | 13.12 | 0.00 | 120,003.83 |
| NaH2PO4 | 0.00 | 0.00 | 0.00 | 0.00 |
| Nitrogen | 607,193.97 | 61.83 | 0.00 | 608,785.58 |
| Oxygen | 162,898.11 | 16.59 | 0.00 | 163,325.11 |
| Sucrose | 0.00 | 0.00 | 0.00 | 0.00 |
| Water | 0.00 | 0.00 | 156.89 | 0.00 |
| TOTAL (kg/batch) | 777,556.62 | 92.26 | 165.33 | 899,286.80 |
| TOTAL (L/batch) | 116,699,735.99 | 76,915.63 | 166.35 | 752,009,974.42 |

| Stream Name | Emissions | Vent SFR-2 | Inoculum to SFR-3 | Vent SFR-3 |
|---|---|---|---|---|
| **Source** | **P-49** | **P-1** | **P-1** | **P-15** |
| **Destination** | **OUTPUT** | **OUTPUT** | **P-15** | **OUTPUT** |
| Stream Properties | | | | |
| Activity (U/ml) | 0.00 | 0.00 | 0.00 | 0.00 |
| Temperature (°C) | 34.98 | 35.00 | 35.00 | 35.00 |
| Pressure (bar) | 1.01 | 1.01 | 1.01 | 1.01 |
| Density (g/L) | 1.20 | 1.18 | 992.80 | 1.18 |
| Total Enthalpy (kW-h) | 16,206.57 | 33.49 | 169.92 | 332.11 |
| Specific Enthalpy (kcal/kg) | 15.51 | 14.06 | 35.10 | 13.95 |
| Heat Capacity (kcal/kg-°C) | 0.24 | 0.24 | 1.00 | 0.24 |
| Component Flowrates (kg/batch) | | | | |
| Ammonium Chlori | 0.00 | 0.00 | 0.03 | 0.00 |
| Argon | 7,172.27 | 16.87 | 0.00 | 168.94 |
| Biomass | 0.00 | 0.00 | 128.94 | 0.00 |
| Carb. Dioxide | 120,003.83 | 217.16 | 0.00 | 2,128.53 |
| NaH2PO4 | 0.00 | 0.00 | 0.01 | 0.00 |
| Nitrogen | 608,785.58 | 1,431.60 | 0.00 | 14,339.59 |
| Oxygen | 163,325.11 | 384.07 | 0.00 | 3,847.03 |
| Sucrose | 0.00 | 0.00 | 0.17 | 0.00 |
| Water | 0.00 | 0.00 | 4,036.68 | 0.00 |
| TOTAL (kg/batch) | 899,286.80 | 2,049.69 | 4,165.83 | 20,484.09 |
| TOTAL (L/batch) | 752,009,974.42 | 1,731,162.84 | 4,196.04 | 17,313,369.52 |

| Stream Name | Inoculum to FR-1 | Mother Liquor | S-116 | S-128 |
|---|---|---|---|---|
| Source | P-15 | P-11 | P-27 | P-4 |
| Destination | P-4 | P-4 | P-4 | OUTPUT |
| Stream Properties | | | | |
| Activity (U/ml) | 0.00 | 0.00 | 0.00 | 0.00 |
| Temperature (°C) | 35.00 | 9.00 | 35.31 | 12.55 |
| Pressure (bar) | 1.01 | 1.01 | 1.01 | 1.01 |
| Density (g/L) | 992.80 | 1,003.90 | 1,012.42 | 1,004.90 |
| Total Enthalpy (kW-h) | 1,697.24 | 8,324.82 | 5,087.67 | 13,417.76 |
| Specific Enthalpy (kcal/kg) | 35.10 | 9.00 | 34.37 | 12.50 |
| Heat Capacity (kcal/kg-°C) | 1.00 | 1.00 | 0.97 | 0.99 |
| Component Flowrates (kg/batch) | | | | |
| Amm. Sulfate | 0.00 | 6.27 | 0.37 | 6.64 |
| Ammonium Chlori | 0.02 | 248.83 | 14.68 | 263.51 |
| Biomass | 1,298.33 | 0.00 | 25,321.47 | 25,321.47 |
| NaH2PO4 | 0.00 | 67.36 | 3.97 | 71.34 |
| pHBA (aq) | 0.00 | 1,584.14 | 4,672.17 | 6,256.31 |
| pHBA (solid) | 0.00 | 1,552.46 | 0.00 | 1,552.46 |
| Sucrose | 0.18 | 5,103.23 | 301.02 | 5,404.25 |
| Water | 40,309.42 | 787,593.98 | 97,033.73 | 884,627.71 |
| TOTAL (kg/batch) | 41,607.95 | 796,156.27 | 127,347.41 | 923,503.69 |
| TOTAL (L/batch) | 41,909.70 | 793,061.60 | 125,784.87 | 919,002.18 |

| Stream Name | S-113 | S-105 | Vent R-101 | S-101 |
|---|---|---|---|---|
| **Source** | **P-4** | **P-27** | **P-28** | **P-28** |
| **Destination** | **P-27** | **P-28** | **OUTPUT** | **P-11** |
| Stream Properties | | | | |
| Activity (U/ml) | 0.00 | 0.00 | 0.00 | 0.00 |
| Temperature (°C) | 35.00 | 35.31 | 5.00 | 5.00 |
| Pressure (bar) | 1.01 | 1.01 | 1.01 | 1.01 |
| Density (g/L) | 1,020.11 | 1,021.32 | 1.26 | 1,033.72 |
| Total Enthalpy (kW-h) | 32,221.46 | 27,418.87 | 1.18 | 3,904.73 |
| Specific Enthalpy (kcal/kg) | 32.41 | 32.41 | 1.23 | 4.61 |
| Heat Capacity (kcal/kg-°C) | 0.92 | 0.91 | 0.24 | 0.92 |
| Component Flowrates (kg/batch) | | | | |
| Amm. Sulfate | 6.64 | 6.27 | 0.00 | 6.27 |
| Ammonium Chlori | 263.51 | 248.83 | 0.00 | 248.83 |
| Argon | 0.00 | 0.00 | 7.57 | 0.00 |
| Biomass | 25,321.47 | 0.00 | 0.00 | 0.00 |
| Carb. Dioxide | 0.00 | 0.00 | 0.33 | 0.00 |
| NaH2PO4 | 71.34 | 67.36 | 0.00 | 67.36 |
| Nitrogen | 0.00 | 0.00 | 642.69 | 0.00 |
| Oxygen | 0.00 | 0.00 | 172.42 | 0.00 |
| pHBA (aq) | 83,879.10 | 79,206.93 | 0.00 | 1,584.14 |
| pHBA (solid) | 0.00 | 0.00 | 0.00 | 77,622.79 |
| Sucrose | 5,404.25 | 5,103.23 | 0.00 | 5,103.23 |
| Water | 740,402.39 | 643,368.66 | 0.00 | 643,368.66 |
| TOTAL (kg/batch) | 855,348.69 | 728,001.28 | 823.01 | 728,001.28 |
| TOTAL (L/batch) | 838,485.37 | 712,801.57 | 651,125.15 | 704,252.88 |

| Stream Name | Wash Water | S-102 | Humid Air | Final Product |
|---|---|---|---|---|
| Source | INPUT | P-11 | P-14 | P-14 |
| Destination | P-11 | P-14 | OUTPUT | OUTPUT |
| Stream Properties | | | | |
| Activity (U/ml) | 0.00 | 0.00 | 0.00 | 0.00 |
| Temperature (°C) | 25.00 | 22.93 | 50.00 | 50.00 |
| Pressure (bar) | 1.01 | 1.86 | 1.01 | 1.01 |
| Density (g/L) | 994.70 | 1,211.06 | 1.08 | 1,303.70 |
| Total Enthalpy (kW-h) | 5,149.97 | 1,407.79 | 45,870.09 | 1,209.42 |
| Specific Enthalpy (kcal/kg) | 25.11 | 11.18 | 24.28 | 13.61 |
| Heat Capacity (kcal/kg-°C) | 1.00 | 0.49 | 0.25 | 0.27 |
| Component Flowrates (kg/batch) | | | | |
| Amm. Sulfate | 0.00 | 0.00 | 0.00 | 0.00 |
| Ammonium Chlori | 0.00 | 0.00 | 0.00 | 0.00 |
| Argon | 0.00 | 0.00 | 14,659.84 | 0.00 |
| Carb. Dioxide | 0.00 | 0.00 | 637.38 | 0.00 |
| NaH2PO4 | 0.00 | 0.00 | 0.00 | 0.00 |
| Nitrogen | 0.00 | 0.00 | 1,244,333.40 | 0.00 |
| Oxygen | 0.00 | 0.00 | 333,830.00 | 0.00 |
| pHBA (aq) | 0.00 | 0.00 | 0.00 | 0.00 |
| pHBA (solid) | 0.00 | 76,070.33 | 0.00 | 76,070.33 |
| Sucrose | 0.00 | 0.00 | 0.00 | 0.00 |
| Water | 176,476.80 | 32,251.48 | 31,869.21 | 382.26 |
| TOTAL (kg/batch) | 176,476.80 | 108,321.81 | 1,625,329.84 | 76,452.60 |
| TOTAL (L/batch) | 177,416.34 | 89,443.43 | 1,511,524,267.60 | 58,642.66 |

# 4. OVERALL COMPONENT BALANCE (kg/batch)

| COMPONENT | INITIAL | INPUT | OUTPUT | FINAL | IN-OUT |
|---|---|---|---|---|---|
| Amm. Sulfate | 0.00 | 284.55 | 6.64 | 0.00 | 277.91 |
| Ammonium Chlori | 0.00 | 11,288.37 | 263.51 | 0.00 | 11,024.86 |
| Argon | 21.98 | 21,999.38 | 22,026.21 | 14.35 | - 19.21 |
| Biomass | 0.00 | 0.00 | 25,321.47 | 0.00 | - 25,321.47 |
| Carb. Dioxide | 0.96 | 956.49 | 123,000.36 | 3.58 | - 122,046.49 |
| NaH2PO4 | 0.00 | 3,056.94 | 71.34 | 0.00 | 2,985.61 |
| Nitrogen | 1,865.54 | 1,867,316.70 | 1,869,594.69 | 1,217.73 | - 1,630.18 |
| Oxygen | 500.49 | 500,964.08 | 501,575.22 | 326.69 | - 437.34 |
| pHBA (aq) | 0.00 | 0.00 | 6,256.31 | 0.00 | - 6,256.31 |
| pHBA (solid) | 0.00 | 0.00 | 77,622.79 | 0.00 | - 77,622.79 |
| Phosphoric Acid | 0.00 | 415.51 | 415.51 | 0.00 | 0.00 |
| Sodium Hydroxid | 0.00 | 633.65 | 633.65 | 0.00 | 0.00 |
| Sucrose | 0.00 | 222,362.10 | 5,404.25 | 0.00 | 216,957.85 |
| Water | 0.00 | 1,006,467.69 | 1,006,467.69 | 0.00 | 0.00 |
| TOTAL | 2,388.96 | 3,635,745.47 | 3,638,659.64 | 1,562.35 | 2,087.56 |
| | | | | Overall Error: | 0,057% |

## 5. EQUIPMENT CONTENTS

### SFR-3

| Procedure | Operation | Time (in h) | Volume (in L) | Vapor (in kg) |
|---|---|---|---|---|
| P-15 | START | 25.61 | 0.00 | 61.77(*) |
| P-15 | TRANSFER-IN-SALTS (Transfer In) | 26.61 | 12,747.01 | 61.77(*) |
| P-15 | TRANSFER-IN-INITIAL-SUGAR (Transfer In) | 27.61 | 35,847.69 | 61.77(*) |
| P-15 | TRANSFER-IN-INOCULUM (Transfer In) | 28.11 | 40,043.76 | 61.77(*) |
| P-15 | FERMENT-2 (Batch Stoich. Fermentation) | 40.11 | 41,909.70 | 12.40(*) |
| P-15 | TRANSFER-OUT-1 (Transfer Out) | 41.11 | 0.00 | 12.40(*) |
| P-15 | CIP-1 (In-Place-Cleaning) | 43.19 | 0.00 | 12.40(*) |
| P-15 | SIP-1 (In-Place-Steaming) | 45.19 | 0.00 | 12.40(*) |

(*) Contains material in vapor phase other than Oxygen & Nitrogen

### SFR-2

| Procedure | Operation | Time (in h) | Volume (in L) | Vapor (in kg) |
|---|---|---|---|---|
| P-1 | START | 14.11 | 0.00 | 6.18(*) |
| P-1 | TRANSFER-IN-SALTS (Transfer In) | 14.61 | 1,274.59 | 6.18(*) |
| P-1 | TRANSFER-IN-INITIAL-SUGAR (Transfer In) | 15.11 | 3,831.40 | 6.18(*) |
| P-1 | TRANSFER-IN-INOCULUM (Transfer In) | 15.61 | 3,997.75 | 6.18(*) |
| P-1 | FERMENT-1 (Batch Stoich. Fermentation) | 27.61 | 4,196.04 | 1.24(*) |
| P-1 | TRANSFER-OUT-1 (Transfer Out) | 28.11 | 0.00 | 1.24(*) |
| P-1 | CIP-1 (In-Place-Cleaning) | 30.19 | 0.00 | 1.24(*) |
| P-1 | SIP-1 (In-Place-Steaming) | 31.19 | 0.00 | 1.24(*) |

(*) Contains material in vapor phase other than Oxygen & Nitrogen

### SFR-1

| Procedure | Operation | Time (in h) | Volume (in L) | Vapor (in kg) |
|---|---|---|---|---|
| P-16 | START | 0.00 | 0.00 | 0.25(*) |
| P-16 | TRANSFER-IN-PHOSPHATE (Transfer In) | 0.25 | 17.10 | 0.25(*) |
| P-16 | TRANSFER-IN-SULFATE (Transfer In) | 0.50 | 34.47 | 0.25(*) |
| P-16 | TRANSFER-IN-NH4Cl (Transfer In) | 0.75 | 51.11 | 0.25(*) |
| P-16 | TRANSFER-IN-INITIAL-SUGAR (Transfer In) | 1.00 | 146.87 | 0.25(*) |
| P-16 | FERMENT (Batch Stoich. Fermentation) | 15.11 | 166.35 | 0.05(*) |
| P-16 | TRANSFER-OUT (Transfer Out) | 15.61 | 0.00 | 0.05(*) |
| P-16 | CIP-1 (In-Place-Cleaning) | 17.69 | 0.00 | 0.05(*) |
| P-16 | SIP-1 (In-Place-Steaming) | 18.19 | 0.00 | 0.05(*) |

(*) Contains material in vapor phase other than Oxygen & Nitrogen

## FR-1

| Procedure | Operation | Time (in h) | Volume (in L) | Vapor (in kg) |
|---|---|---|---|---|
| P-4 | START | 39.11 | 0.00 | 1,156.42(*) |
| P-4 | TRANSFER-IN-SULFATE (Transfer In) | 40.11 | 86,652.16 | 1,156.42(*) |
| P-4 | TRANSFER-IN-NH4Cl (Transfer In) | 40.11 | 169,672.96 | 1,156.42(*) |
| P-4 | TRANSFER-IN-PHOSPHATE (Transfer In) | 40.11 | 254,942.14 | 1,156.42(*) |
| P-4 | TRANSFER-IN-INITIAL-SUGAR (Transfer In) | 40.11 | 522,348.47 | 1,156.42(*) |
| P-4 | TRANSFER-IN-INOCULUM (Transfer In) | 41.11 | 564,258.68 | 1,156.42(*) |
| P-4 | FERMENT-1 (Batch Stoich. Fermentation) | 76.27 | 838,485.37 | 170.04(*) |
| P-4 | TRANSFER-OUT-2 (Transfer Out) | 76.11 | 0.00 | 1,126.70(*) |
| P-4 | TRANSFER-IN-1 (Transfer In) | 76.11 | 125,784.86 | 981.20(*) |
| P-4 | TRANSFER-IN-2 (Transfer In) | 76.11 | 919,002.18 | 76.43(*) |
| P-4 | TRANSFER-OUT-1 (Transfer Out) | 78.27 | 0.00 | 1,207.34(*) |
| P-4 | CIP-1 (In-Place-Cleaning) | 80.35 | 0.00 | 1,207.34(*) |
| P-4 | SIP-1 (In-Place-Steaming) | 82.35 | 0.00 | 1,207.34(*) |

(*) Contains material in vapor phase other than Oxygen & Nitrogen

## R-102

| Procedure | Operation | Time (in h) | Volume (in L) | Vapor (in kg) |
|---|---|---|---|---|
| P-28 | START | 40.11 | 0.00 | 155.65(*) |
| | AFTER AUTO-INIT | 40.11 | 118,800.26 | 155.65(*) |
| P-28 | REACT-1 (Batch Stoich. Reaction) | 76.11 | 117,375.48 | 18.49(*) |
| P-28 | END | 76.11 | 0.00 | 18.49(*) |

(*) Contains material in vapor phase other than Oxygen & Nitrogen

## BCFBD-101

| Procedure | Operation | Time (in h) | Volume (in L) | Vapor (in kg) |
|---|---|---|---|---|
| P-11 | START | 40.11 | 0.00 | 19.20(*) |
| P-11 | FILTER-1 (Cloth Filtration) | 75.61 | 7,392.35 | 19.20(*) |
| P-11 | CAKE-WASH-1 (Cake Wash) | 75.86 | 7,453.62 | 19.20(*) |
| P-11 | TRANSFER-OUT-1 (Transfer Out) | 76.11 | 0.00 | 19.20(*) |

(*) Contains material in vapor phase other than Oxygen & Nitrogen
