# Supplementary material for: Economic Process Evaluation and Environmental Life-Cycle Assessment of Bio-Aromatics Production
Source: Front Bioeng Biotechnol. 2020 May 13;8:403. doi: 10.3389/fbioe.2020.00403 (PMC7237583; doi:10.3389/fbioe.2020.00403)
Supplement: Supplementary file 1 [file Data_Sheet_1.zip › Supplementary Information.docx]

Supplementary Information

**Economic Process Evaluation and Environmental Life-Cycle Assessment of bio-aromatics production**

Jens O. Krömer^1*^, Rafael G. Ferreira^2^, Demetri Petrides^3^ & Norbert Kohlheb^4^

^1^Systems Biotechnology, Department of Solar Materials, Helmholtz Centre for Environmental Research – UFZ, Leipzig, Germany.

^2^Intelligen Brasil, Sao Paulo, SP, Brazil.

^3^Intelligen Inc., Scotch Plains, NJ, USA.

^4^Department Environmental and Biotechnology Centre, Helmholtz Centre for Environmental Research – UFZ, Leipzig, Germany.

List of files

Supplementary_Calculations_results.xlsx – Contains modelling calculations, LCA inventory, and results

Supplementary_1_reference_system_chemical_synthesis.spf – The SuperPro model of the reference system: the Kolbe-Schmitt reaction

Supplementary_2_bacterial_base_case.spf – The base scenario of the bacterial process with cane sugar substrate

Supplementary_3_bacterial_base_case_biomass_recycling.spf – The base scenario with biomass recycling with cane sugar substrate

Supplementary_4_bacterial_base_case_water_recycling.spf – The base scenario with water recycling with cane sugar substrate

Supplementary_5_bacterial_best_case_cane_sugar.spf – The base scenario with both water and biomass recycling and with cane sugar substrate

Supplementary_6_bacterial_best_case_cane_sugar_upscaled.spf – The base scenario with both water and biomass recycling and with cane sugar substrate and with fivefold of base production

Supplementary_7_bacterial_best_case_beet_sugar_upscaled.spf – The base scenario with both water and biomass recycling and with beet sugar substrate and with fivefold of base production

Supplementary_8_yeast_base_case.spf – The base scenario with yeast

Supplementary_9_yeast_best_case_cane_sugar_upscaled.spf – The base scenario with yeast with both water and biomass recycling and with cane sugar substrate and with fivefold of base production

Supplementary_10_yeast_best_case_beet_sugar_upscaled.spf – The base scenario with both water and biomass recycling and with beet sugar substrate and with fivefold of base production

Scenarios with different fermentation times:

Supplementary_11_bacterial_base_case.spf

Supplementary_12_bacterial_best_case_cane_sugar_upscaled.spf

Supplementary_13_bacterial_best_case_cane_sugar_upscaled.spf

Supplementary_14_yeast_base_case.spf

Supplementary_15_yeast_base_case.spf

Supplementary_16_yeast_best_case_cane_sugar_upscaled.spf

Supplementary_17_yeast_best_case_cane_sugar_upscaled.spf

Material flows of the 17 scenarios

Sc_1.pdf

Sc_2.pdf

Sc_3.pdf

Sc_4.pdf

Sc_5.pdf

Sc_6.pdf

Sc_7.pdf

Sc_8.pdf

Sc_9.pdf

Sc_10.pdf

Sc_11.pdf

Sc_12.pdf

Sc_13.pdf

Sc_14.pdf

Sc_15.pdf

Sc_16.pdf

Sc_17.pdf
